# Steroidal saponins from the genus *Allium*

**DOI:** 10.1007/s11101-014-9381-1

**Published:** 2014-10-08

**Authors:** Danuta Sobolewska, Klaudia Michalska, Irma Podolak, Karolina Grabowska

**Affiliations:** 1 Department of Pharmacognosy, Jagiellonian University, Medical College, 9 Medyczna Street, Kraków, Poland; 2 Department of Phytochemistry, Institute of Pharmacology, Polish Academy of Sciences, 12 Smętna Street, Kraków, Poland

**Keywords:** *Allium*, Steroidal saponins, Saponins activity

## Abstract

Steroidal saponins are widely distributed among monocots, including the Amaryllidaceae family to which the *Allium* genus is currently classified. Apart from sulfur compounds, these are important biologically active molecules that are considered to be responsible for the observed activity of *Allium* species, including antifungal, cytotoxic, enzyme-inhibitory, and other. In this paper, literature data concerning chemistry and biological activity of steroidal saponins from the *Allium* genus has been reviewed.

**Electronic supplementary material:**

The online version of this article (doi:10.1007/s11101-014-9381-1) contains supplementary material, which is available to authorized users.

## Introduction

The genus *Allium* (Amaryllidaceae) is one of the largest monocot genera comprising more than 800 species (Li et al. 2010; APG 2009). It is widely distributed in nature and has adapted to diverse habitats across the Holarctic region, with the exception of *A*. *dregeanum*, which is native to South Africa (Li et al. 2010). Some *Allium* species, such as garlic, onion and leek, are widely cultivated as vegetable products, spices and for medical purposes. The most characteristic constituents in *Allium* plants are sulfur compounds, which are the most important substances both in terms of chemotaxonomic value and biological activity (Rose et al. 2005). However, various researchers tend to attribute the potential pharmacological benefits of *Allium* plants to constituents other than sulfur compounds, such as steroidal saponins. Also, polyphenolic compounds, especially flavonoids, as well as fructans, *N*-cynnamic amides, and antioxidative enzymes are considered to be equally important (Matsuura 2001; Lanzotti 2005; Štajner et al. 2006; Amagase 2006; Lanzotti 2012).

Apart from the Amaryllidaceae family, steroidal saponins are widely distributed in other monocot families: Asparagaceae (*Agave*, *Asparagus*, *Convallaria*, *Hosta*, *Nolina*, *Ornithogalum*, *Polygonatum*, *Sansevieria*, *Yucca*), Costaceae (*Costus*), Dioscoreaceae (*Dioscorea*), Liliaceae (*Lilium*), Melanthiaceae (*Paris*), Smilacaceae (*Smilax*). Interestingly, these compounds have been reported as well in some dicotyledonous angiosperms: Zygophyllaceae (*Tribulus*, *Zygophyllum*), Solanaceae (*Solanum*, *Lycopersicon*, *Capsicum*), Plantaginaceae (*Digitalis*) and Fabaceae (*Trigonella*).

There are numerous reports referring to pharmacological activities of steroidal saponins. Some of them showed promising antifungal, cytotoxic, anti-inflammatory, antithrombotic, and hypocholesterolemic effects (Sparg et al. 2004; Lanzotti 2005; Güçlü-Üstündağ and Mazza 2007). Most importantly, these compounds are used as substrates in the production of steroid hormones and drugs.

Steroidal sapogenins and saponins have been identified so far in over 40 different *Allium* species. The earliest reports on *Allium* saponins date back to the 1970s and dealt with identification of diosgenin in *A. albidum* (Kereselidze et al. 1970) and alliogenin in the bulbs of *A. giganteum* (Khristulas et al. 1970). Further studies performed worldwide in the following years led to the isolation of a large number of new compounds. The first chemical survey of saponins from the genus *Allium* was published by Kravets in 1990, and this was followed by an update by Lanzotti in 2005 (Kravets et al. 1990; Lanzotti 2005). Since then, a large number of new compounds has been discovered, and there were also some that have not been included in the previous surveys.

A recent review by Lanzotti et al. (2014) compiled data on various compounds identified in *Allium* species with a reported cytotoxic and antimicrobial activity.

The present review is predominantly focused on the chemistry of *Allium* steroidal saponins and their biological activities.

## Chemistry of *Allium* saponins

Steroidal saponins from the genus *Allium* can be divided into three groups on the basis of the sapogenin structure: spirostanols, furostanols, and open-chain saponins. The latter group is often referred to in the literature as “cholestane saponins” (Challinor and De Voss 2013). *Allium* saponins are mostly mono- or bidesmosides, however a tridesmodic cholestane glycoside has been reported in the bulbs of *A. macleanii* (Inoue et al. 1995). The sugar residue in *Allium* saponins consists of linear or branched chains made up most often of glucose (Glc), rhamnose (Rha), galactose (Gal), xylose (Xyl), and arabinose (Ara) units.

### Spirostane-type saponins

A vast structural diversity of *Allium* spirostanols is associated with the differences in the structure of aglycones, especially their oxygenation patterns and stereochemistry (Table 1). In spirostane-type sapogenins, the steroid A/B ring junction is found mostly in a *trans* (5α), or more rarely in a *cis* (5β) fusion (e.g. anzurogenin A [**48**] and C [**58**]). Δ^5(6)^ unsaturation is considered to be a quite common feature (diosgenin [**4**], ruscogenin [**17**], yuccagenin [**19**], lilagenin [**20**], cepagenin [**44**], karatavigenin C [**45**]). However, a double bond located at C25(27) was reported in the aglycones of saponins present in *A*. *macrostemon* and in one of the sapogenins identified in *A*. *ursinum* bulbs (He et al. 2002; Sobolewska et al. 2006; Cheng et al. 2013). The C-25 methyl group is found with either *S* or *R* absolute configuration. In many cases the isolated sapogenins appear to be a mixture of diastereomers *R* and *S*.

**Table 1:** Spirostane-type sapogenins identified in the genus *Allium*

No.	Common name	Structure	Species
[**1**]	Tigogenin	(25*R*)-5α-spirostane-3β-ol	*A. affine*, *A. chinense*, *A. fistulosum*, *A. macleanii*, *A. macrostemon*, *A. rotundum*, *A. sativum*
[**2**]	Neotigogenin	(25*S*)-5α-spirostane-3β-ol	*A. chinense*, *A. tuberosum*
[**3**]	Smilagenin	(25*R*)-5β-spirostane-3β-ol	*A. macrostemon*
[**4**]	Diosgenin	(25*R*)-spirost-5(6)-ene-3β-ol	*A. affine*, *A. albidum*, *A. ampeloprasum*, *A. angulosum*, *A. cepa*, *A. cernuum*, *A. fistulosum*, *A. flavum*, *A. fuscoviolaceum*, *A. giganteum*, *A. gramineum*, *A. karataviense*, *A. narcissiflorum*, *A. nutans*, *A. porrum*, *A. rotundum*, *A. schoenoprasum*, *A. senescens*, *A. ursinum*, *A. vineale*, *A. waldsteinii*
[**5**]		(25*R*)-spirost-5(6),25(27)-diene-3β-ol	*A. ursinum*
[**6**]	Laxogenin	(25*R*)-5α-spirostane-3β-ol-6-one	*A. chinense*, *A. schoenoprasum*
[**7**]	Hecogenin	(25*R*)-5α-spirostane-3β-ol-12-one	*A. albidum*, *A. rotundum*
[**8**]		(25*S*)-5β-spirostane-1β,3β-diol	*A. tuberosum*
[**9**]	Gitogenin	(25*R*)-5α-spirostane-2α,3β-diol	*A. aflatunense*, *A. chinense*, *A. cyrillii*, *A. elburzense*, *A. fistulosum*, *A. hirtifolium*, *A. jesdianum*, *A. macrostemon*, *A. porrum*, *A. rotundum*, *A. sativum*, *A. sativum* L. var. Voghiera, *A. victorialis* var*. platyphyllum*
[**10**]	Neogitogenin	(25*S*)-5α-spirostane-2α,3β-diol	*A. chinense*, *A. tuberosum*
[**11**]		(25*S*)-5β-spirostane-2β,3β-diol	*A. tuberosum*
[**12**]	β-Chlorogenin	(25*R*)-5α-Spirostane-3β,6β-diol	*A. erubescens*, *A. giganteum*, *A. gramineum*, *A. leucanthum*, *A. porrum*, *A. rotundum*, *A. sativum*, *A. waldsteinii*
[**13**]	25-Epi-ruizgenin	(25*S*)-5β-spirostane-3β,6α-diol	*A. tuberosum*
[**14**]		(25*R*)-5α-Spirostane-3β,11α-diol	*A. schoenoprasum*
[**15**]		(25*R*)-5β-spirostane-3β,12β-diol	*A. macrostemon*
[**16**]		(25*S*)-5α-spirostane-3β,24β-diol	*A. chinense*
[**17**]	Ruscogenin	(25*R*)-spirost-5(6)-ene-1β,3β-diol	*A. affine*, *A. albidum*, *A. nutans*
[**18**]	(25*S*)-ruscogenin	(25*S*)-spirost-5(6)-ene-1β,3β-diol	*A. cepa*
[**19**]	Yuccagenin	(25*R*)-spirost-5(6)-ene-2α,3β-diol	*A. ampeloprasum*, *A. fistulosum*, *A. flavum*, *A. giganteum*, *A. karataviense*, *A. rotundum*, *A. turcomanicum*
[**20**]	Lilagenin	(25*S*)-spirost-5(6)-ene-2α,3β-diol	*A. tuberosum*
[**21**]		5β-Spirost-25(27)-ene-2β,3β-diol	*A. macrostemon*
[**22**]		5β-spirost-25(27)-ene-3β,12β-diol	*A. macrostemon*
[**23**]	Porrigenin B	(25R)-5α-spirostane-3β,6β-diol-2-one	*A. ampeloprasum, A. porrum*
[**24**]	Neoporrigenin B	(25*S*)-5α-spirostane-3β,6β-diol-2-one	*A. porrum*
[**25**]	Neoagigenone	(25*S*)-5α-spirostane-2α,3α-diol-6-one	*A. turcomanicum*
[**26**]	Anzurogenin B	(25*R*)-5α-spirostane-2α,5α-epoxy-3β,6β-diol	*A. stipitatum/A. suvorovii*
[**27**]	Nuatigenin	(22*S*,25*S*)-22,25-epoxy-furost-5(6)-ene-3β,26-diol	*A. vineale*
[**28**]	Izonuatigenin	(25*R*)-spirost-5(6)-ene-3β,25β-diol	*A. vineale*
[**29**]	12-Ketoporrigenin	(25*R*)-5α-spirostane-3β,6β-diol-12-one	*A. porrum*
[**30**]	Porrigenin C	(25*R*)-5α-spirostane-3β,6β-diol-2,12-dione	*A. porrum*
[**31**]	Agapanthagenin	(25*R*)-5α-spirostane-2α,3β,5α-triol	*A. aflatunense*, *A. elburzense*, *A. hirtifolium*
[**32**]		(25*S*)-5β-spirostane-2β,3β,5β-triol	*A. tuberosum*
[**33**]	Gantogenin	(25*R*)-5α-spirostane-2α,3β,6α-triol	*A. giganteum*, *A. jesdianum*
[**34**]	Agigenin	(25R)-5α-spirostane-2α,3β,6β-triol	*A. albopilosum, A. ampeloprasum, A. atroviolaceum, A. giganteum, A. gramineum, A. hirtifolium, A. leucanthum, A. macleanii, A. ostrowskianum, A. porrum, A. rotundum, A. sativum var*. Voghiera, *A. schubertii*
[**35**]	2-O-[(S)-3-hydroxy-3-methylglutaryl]-agigenin	2-O-[(S)-3-hydroxy-3-methylglutaryl]-(25R)-5α-spirostane-2α,3β,6β-triol	*A. albopilosum*
[**36**]	Neoagigenin	(25*S*)-5α-spirostane-2α,3β,6β-triol	*A. albopilosum*, *A. ampeloprasum* ssp. *persicum*, *A. giganteum*, *A. minutiflorum*, *A. nigrum*, *A. porrum*, *A. schubertii*, *A. turcomanicum*
[**37**]	6-*O*-benzoyl-neoagigenin	6-*O*-benzoyl-(25*S*)-5α-spirostane-2α,3β,6β-triol	*A. turcomanicum*
[**38**]	Porrigenin A	(25*R*)-5α-spirostane-2β,3β,6β-triol	*A. porrum*
[**39**]	Neoporrigenin A	(25*S*)-5α-spirostane-2β,3β,6β-triol	*A. porrum*
[**40**]		(25*R*)-5α-spirostane-2α,3β,27-triol	*A. tuberosum*
[**41**]	Anzurogenin D	(25*R*)-5α-spirostane-3β,5α,6β-triol	*A. stipitatum/A. suvorovii*
[**42**]		(25*S*)-5α-spirostane-2α,3β,27-triol	*A. tuberosum*
[**43**]		(25*S*)-5β-spirostane-3β,5β,6α-triol	*A. tuberosum*
[**44**]	Cepagenin	(24*S*,25*R*)-spirost-5(6)-ene-1β,3β,24-triol	*A. cepa*
[**45**]	Karatavigenin C	(24*S*,25*S*)-spirost-5(6)-ene-2α,3β,24-triol	*A. karataviense*
[**46**]		(20S,25S)-spirost-5(6)-ene-3β,11α,21-triol	*A. schoenoprasum*
[**47**]		(20S,25S)-spirost-5(6)-ene-3β,12β,21-triol	*A. schoenoprasum*
[**48**]	Anzurogenin A	(25*R*)-5β-spirostane-2α,3β,5β-triol-6-one	*A. stipitatum*/*A. suvorovii*
[**49**]	Alliogenin	(25*R*)-5α-spirostane-2α,3β,5α,6β-tetrol	*A. aflatunense*, *A. albopilosum*, *A. elburzense*, *A. giganteum,* *A. hirtifolium*, *A. karataviense*, *A. macleanii*, *A. minutiflorum*, *A. turcomanicum*
[**50**]	Neoalliogenin	(25*S*)-5α-spirostane-2α,3β,5α,6β-tetrol	*A. turcomanicum*
[**51**]	3-O-acetyl-alliogenin	3-O-acetyl-(25R)-5α-spirostane-2α,3β,5α,6β-tetrol	*A. albopilosum, A. giganteum, A. karataviense*
[**52**]	Karatavigenin (3-*O*-benzoyl-alliogenin)	3-*O*-benzoyl-(25*R*)-5α-spirostane-2α,3β,5α,6β-tetrol	*A. giganteum*, *A. karataviense*, *A. macleanii*
[**53**]	Karatavigenin B (2-*O*-benzoyl-alliogenin)	2-*O*-benzoyl-(25*R*)-5α-spirostane-2α,3β,5α,6β-tetrol	*A. karataviense*
[**54**]	3-*O*-(2-hydroxybutyryl)-alliogenin	3-*O*-(2-hydroxybutyryl)-(25*R*)-5α-spirostane-2α,3β,5α,6β-tetrol	*A. karataviense*
[**55**]		(24*S*,25*S*)-5β-spirostane-2α,3β,5β,24-tetrol	*A. tuberosum*
[**56**]		(24*S*,25*S*)-5β-spirostane-2β,3β,5β,24-tetrol	*A. tuberosum*
[**57**]	Atroviolacegenin	(25*R*)-5α-spirostane-2α,3β,6β,27-tetrol	*A. atroviolaceum*
[**58**]	Anzurogenin C	(24*S*,25*S*)-5β-spirostane-2α,3β,5,24-tetrol-6-one	*A. stipitatum*/*A. suvorovii*
[**59**]	Luvigenin	(25R)-4-methyl-19-norspirosta-l,3,5(10)-triene	*A. giganteum*
[**60**]		(24S,25R)-5α-spirostane-2α,3β,5α,6β,24-pentaol	*A. giganteum*
[**61**]		(24S,25S)-5α-spirostane-2α,3β,5α,6β,24-pentaol	*A. karataviense*
[**62**]		3-O-acetyl-(24S,25S)-5α-spirostane-2α,3β,5α,6β,24-pentaol	*A. giganteum*
[**63**]		3-O-benzoyl-(24S,25S)-5α-spirostane-2α,3β,5α,6β,24-pentaol	*A. karataviense*
[**64**]	2,3-Seco-porrigenin	(25*R*)-5α-2,3-secospirostane-2,3-dioic acid-6β-hydroxy-3,6-γ-lactone	*A. porrum*

The most common spirostanol sapogenins identified in *Allium* plants are: diosgenin [**4**], tigogenin [**1**], gitogenin [**9**], agigenin [**34**], alliogenin [**49**], and β-chlorogenin [**12**]. It was claimed that β-chlorogenin, a genin present in common garlic *A. sativum*, could be considered as a chemical marker for its identification in various food products, as the characteristic garlic sulfur compounds are very unstable (Itakura et al. 2001).

Until now, over 130 spirostanol glycosides have been identified in various *Allium* species. It should be mentioned however that some of these compounds were obtained as a result of enzymatic hydrolysis of furostanol saponin fraction by β-glucosidase (Ikeda et al. 2000).

*Allium* spirostane-type saponins are typically monodesmodic with the sugar residue usually at C-3 position. In rare cases, the sugar moiety was reported to be linked at other positions, such as C-1 (e.g. alliospirosides A-D [**169**, **170**, **178**, **179**]) (Kravets et al. 1986a, b, 1987), C-2 (compounds from *A. giganteum* and *A. albopilosum*) (Sashida et al. 1991), C-24 (chinenoside VI [**116**], karatavioside F [**181**], and anzuroside [**190**]), or C-27 (tuberoside L [**104**]) (Jiang et al. 1998; Vollerner et al. 1984; Vollerner et al. 1989; Sang et al. 2001a).

Table 3 of ESM summarizes chemical structures of spirostane-type saponins that were reported in *Allium* species.

### Furostane-type saponins

Furostanol aglycones possess either a *cis* or a *trans* fusion between ring A and B, or a double bond between C-5 and C-6 leading to 5α, 5β or Δ^5(6)^ series. In the case of furostane-type sapogenins a double bond may also be located at 20(22) (e.g. ascalonicoside B [**220**], ceparoside C [**230**], chinenoside II [**234**]) or 22(23) (four furostanols from *A. tuberosum*) (Fattorusso et al. 2002; Yuan et al. 2009; Peng et al. 1996b; Sang et al. 2001b). The 27-Me group may be in either *R* or *S* configuration. Furostane-type compounds isolated from *Allium* species usually possess an OH or OMe group at C-22. However, sapogenins with a C-22 methyl ether are considered to be artifacts resulting from the use of methanol in the extraction/isolation procedures.

From among 140 furostanol glycosides identified in the *Allium* genus, sixteen compounds were found to be such methoxy-derivatives.

Furostanol saponins in *Allium* plants are bidesmodic glycosides with sugar chains attached usually at C-3 and C-26 positions. A rare glycosylation at C-1 with a galactose unit was reported in ascalonicosides A1/A2 [**217**, **218**] (Fattorusso et al. 2002). A vast majority of furostanol saponins possess an O-linked glucose residue attached at position C-26. In compounds such as ceposides, persicoside C [**205**, **206**], ascalonicosides A1/A2 [**217**, **218**] a disaccharide chain was reported at C-26 (Lanzotti 2012; Sadeghi et al. 2013; Fattorusso et al. 2002).

### Cholestane-type (open-chain) saponins

A review of available literature data shows that as much as 18 cholestane-type compounds have been identified in ten different *Allium* species.

**Fig. 1:**
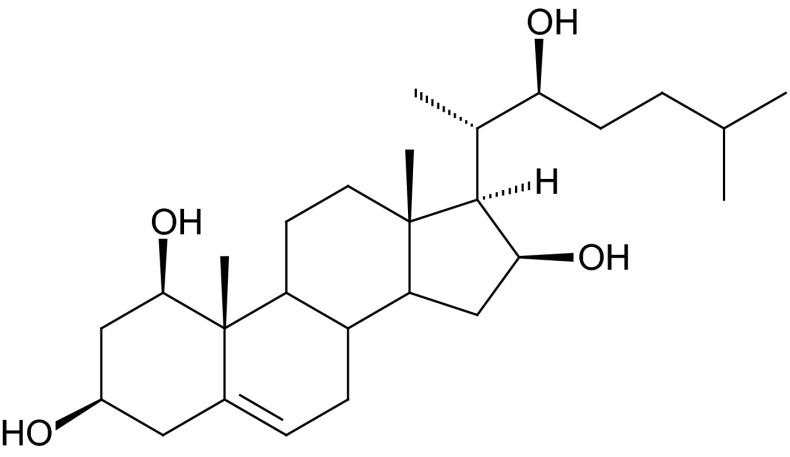
Alliosterol—(22*S*)-cholest-5(6)-ene-1β,3β,16β,22-tetrol [**196**]

*Allium* open-chain aglycones possess Δ^5(6)^ unsaturation with an exception of schubertoside A [**329**]**—**Δ^4(5)^, and one of the glycosides found in *A. albopilosum* with a saturated aglycone (Kawashima et al. 1991b; Mimaki et al. 1993). Glycosides based on alliosterol—(22*S*)-cholest-5(6)-ene-1β,3β,16β,22-tetrol (Fig. 1 [**196**]**)**, or related sapogenins showing the same oxygenation pattern at C-1, C-3, C-16 and C-22 are most common (Challinor and De Voss 2013). Sugar units are attached at one, two or, more seldom, at three separate positions (in *A. macleanii*) (Inoue et al. 1995). Most of these compounds are glycosylated at C-16, whereas in contrast to spirostanol and furostanol saponins, the attachment of sugar chain at position C-3 is almost unique (tuberoside U [**353**]) (Sang et al. 2003).

Table 2 lists steroidal saponins/sapogenins identified in *Allium* species. Plant names are cited exactly as they were referred to in the original report. It is almost certain that some of them are synonyms but as the authors of the present review are not specialists in plant taxonomy no amendments have been made.

**Table 2:** List of steroidal saponins/sapogenins reported in *Allium* species

Species	Glycoside common name [no.]	Sapogenin [no.]	Sugar residue	References
*A. affine* Ledeb.		Tigogenin [**1**]		Kravets et al. (1990)
		Diosgenin [**4**]		Kravets et al. (1990)
		Ruscogenin [**17**]		Kravets et al. (1990)
*A. aflatunense* B. Fedtsch.	[**70**]	Gitogenin [**9**]	3-*O*-β-d-Glc-(1 → 4)-*O*-β-d-Gal	Mimaki et al. (1999c)
	[**75**]	Gitogenin [**9**]	3-*O*-β-d-Glc-(1 → 2)-[4-*O*-(*S*)-3-hydroxy-3-methylglutaryl-β-d-Xyl-(1 → 3)]-*O*-β-d-Glc-(1 → 4)-*O*-β-d-Gal	Mimaki et al. (1999c)
	[**84**]	Agapanthagenin [**31**]	2-*O*-β-d-Glc	Mimaki et al. (1999c)
	[**105**]	Alliogenin [**49**]	2-*O*-β-d-Glc	Kawashima et al. (1991a)
*A. albanum* Grossh			Saponins present	Ismailov et al. (1976)
*A. albidum* Fisch. Ex M. Bieb.		Diosgenin [**4**]		Kereselidze et al. (1970)
		Hecogenin [**7**]		Kravets et al. (1990)
		Ruscogenin [**17**]		Pkheidze et al. (1971)
*A. albiflorus*			Saponins present	Ismailov and Aliev (1976)
*A. albopilosum* C.H. Wright		Agigenin [**34**]		Mimaki et al. (1993)
		Neoagigenin [**36**]		Mimaki et al. (1993)
		Alliogenin [**49**]		Mimaki et al. (1993)
	AGINOSIDE [**93**]	Agigenin [**34**]	3-*O*-β-d-Glc-(1 → 2)-[β-d-Xyl-(1 → 3)]-*O*-β-d-Glc-(1 → 4)-*O*-β-d-Gal	Mimaki et al. (1993)
	[**94**, **123**]	(25*R*,*S*)-5α-spirostane-2α,3β,6β-triol [**34**, **36**]	3-*O*-β-d-Glc-(1 → 2)-[3-*O*-acetyl-β-d-Xyl-(1 → 3)]-*O*-β-d-Glc-(1 → 4)-*O*-β-d-Gal	Mimaki et al. (1993)
	[**101**]	2-*O*-[(*S*)-3-hydroxy-3-methylglutaryl]-agigenin [**35**]	3-*O*-β-d-Glc-(1 → 2)-[β-d-Xyl-(1 → 3)]-*O*-β-d-Glc-(1 → 4)-*O*-β-d-Gal	Mimaki et al. (1993)
	[**105**]	Alliogenin [**49**]	2-*O*-β-d-Glc	Mimaki et al. (1993)
	[**184**]	3-*O*-acetyl-alliogenin [**51**]	2-*O*-β-d-Glc	Mimaki et al. (1993)
	[**197**, **198**]	(25*R*,*S*)-5α-furostane-2α,3β,6β,22,26-pentaol	26-*O*-β-d-Glc 3-*O*-β-d-Glc-(1 → 2)-[β-d-Xyl-(1 → 3)]-*O*-β-d-Glc-(1 → 4)-*O*-β-d-Gal	Mimaki et al. (1993)
	[**199**]	Alliosterol [(22*S*)-cholest-5(6)-ene-1β,3β,16β,22-tetrol] [**196**]	1-*O*-α-l-Rha 16-*O*-α-l-Rha-(1 → 3)-*O*-β-d-Glc	Mimaki et al. (1993)
	[**200**]	Cholest-5(6)-ene-1β,3β,16β-triol-22-one	1-*O*-α-l-Rha 16-*O*-α-l-Rha-(1 → 3)-*O*-β-d-Glc	Mimaki et al. (1993)
	[**201**]	5α-Cholestane-1β,3β,16β-triol-22-one	1-*O*-α-l-Rha 16-*O*-α-l-Rha-(1 → 3)-*O*-β-d-Glc	Mimaki et al. (1993)
*A. ampeloprasum* L.		Agigenin [**34**]		Morita et al. (1988)
	[**87**]	Agigenin [**34**]	3-*O*-β-d-Glc-(1 → 4)-*O*-β-d-Gal	Morita et al. (1988)
	AMPELOSIDE Bs_1_ [**90**]	Agigenin [**34**]	3-*O*-β-d-Glc-(1 → 3)-*O*-β-d-Glc-(1 → 4)-*O*-β-d-Gal	Morita et al. (1988)
	AGINOSIDE [**93**]			Sata et al. (1998)
	YAYOISAPONIN C [**95**]	Agigenin [**34**]	3-*O*-β-d-Glc-(1 → 2)-[β-d-Glc-(1 → 3)]-*O*-β-d-Glc-(1 → 4)-*O*-β-d-Gal	Sata et al. (1998)
	YAYOISAPONIN A [**96**]	Agigenin [**34**]	3-*O*-β-d-Glc-(1 → 3)-*O*-β-d-Glc-(1 → 2)-[β-d-Xyl-(1 → 2)]-*O*-β-d-Glc-(1 → 4)-*O*-β-d-Gal	Sata et al. (1998)
	DIOSCIN [**135**]	Diosgenin [**4**]	3-*O*-α-l-Rha-(1 → 2)-[α-l-Rha-(1 → 4)]-*O*-β-d-Glc	Sata et al. (1998)
	KARATAVIOSIDE A [**151**]	Yuccagenin [**19**]	3-*O*-β-d-Glc-(1 → 2)-[β-d-Xyl-(1 → 3)]-*O*-β-d-Glc-(1 → 4)-*O*-β-d-Gal	Uchida et al. (2009)
	YAYOISAPONIN B [**174**]	Porrigenin B [**23**]	3-*O*-β-d-Glc-(1 → 3)-*O*-β-d-Glc-(1 → 2)-[β-d-Xyl-(1 → 3)]-*O*-β-d-Glc-(1 → 4)-*O*-β-d-Gal	Sata et al. (1998)
	AMPELOSIDE Bf_1_ [**202**]	(25*R*)-5α-furostane-2α,3β,6β,22,26-pentaol	26-*O*-β-d-Glc 3-*O*-β-d-Glc-(1 → 3)-*O*-β-d-Glc-(1 → 4)-*O*-β-d-Gal	Morita et al. (1988)
	AMPELOSIDE Bf_2_ [**203**]	(25*R*)-5α-furostane-2α,3β,6β,22,26-pentaol	26-*O*-β-d-Glc 3-*O*-β-d-Glc-(1 → 4)-*O*-β-d-Gal	Morita et al. (1988)
	[**204**]	(25*R*)-5α-furostane-3β,26-diol	26-*O*-β-d-Glc 3-*O*-β-d-Glc-(1 → 2)-*O*-β-d-Xyl-(1 → 3)-*O*-β-d-Glc-(1 → 4)-*O*-β-d-Gal	Mimaki et al. (1999b)
*A.* *ampeloprasum* L. ssp. *persicum*	PERSICOSIDE A [**120**]	Neoagigenin [**36**]	3-*O*-β-d-Glc-(1 → 3)-[β-d-Xyl-(1 → 2)]-*O*-β-d-Glc-(1 → 4)-*O*-β-d-Gal	Sadeghi et al. (2013)
	PERSICOSIDE B [**121**]	Neoagigenin [**36**]	3-*O*-β-d-Xyl-(1 → 3)-[α-l-Rha-(1 → 2)]-*O*-β-d-Glc-(1 → 4)-*O*-β-d-Gal	Sadeghi et al. (2013)
	PERSICOSIDE C (C1/C2) [**205**, **206**]	Furost-5(6)-ene-1β,3β,22ξ,26-tetrol	26-*O*-α-l-Rha-(1 → 2)-β-d-Gal 1-*O*-β-d-Glc-(1 → 3)-*O*-β-d-Glc-(1 → 2)-*O*-β-d-Gal	Sadeghi et al. (2013)
	PERSICOSIDE D (D1/D2) [**207**, **208**]	Furostane-2α,3β,22ξ,26-tetrol	26-*O*-β-d-Glc 3-*O*-β-d-Glc-(1 → 3)-*O*-β-d-Glc-(1 → 2)-*O*-β-d-Gal	Sadeghi et al. (2013)
	CEPOSIDES A1/A2 [**209**, **210**]	(25*R*)-furost-5(6)-ene-1β,3β,22ξ,26-tetrol	26-*O*-α-l-Rha-(1 → 2)-*O*-β-d-Gal 1-*O*-β-d-Xyl	Sadeghi et al. (2013)
	CEPOSIDES C1/C2 [**211**, **212**]	(25*R*)-furost-5(6)-ene-1β,3β,22ξ,26-tetrol	26-*O*-α-l-Rha-(1 → 2)-*O*-β-d-Gal 1-*O*-β-d-Gal	Sadeghi et al. (2013)
	TROPEOSIDES A1/A2 [**213**, **214**]	Furost-5(6)-ene-3β,22ξ-diol	26-*O*-α-l-Rha 1-*O*-β-d-Gal	Sadeghi et al. (2013)
	TROPEOSIDES B1/B2 [**215**, **216**]	Furost-5(6)-ene-3β,22ξ-diol	26-*O*-α-l-Rha 1-*O*-β-d-Xyl	Sadeghi et al. (2013)
	ASCALONICOSIDES A1/A2 [**217**, **218**]	Furost-5(6)-ene-3β,22ξ-diol	26-*O*-α-l-Rha-(1 → 2)-*O*-β-d-Glc 1-*O*-β-d-Gal	Sadeghi et al. (2013)
	PERSICOSIDE E [**219**]	Alliosterol [**196**]	1-*O*-α-l-Rha 16-*O*-α-l-Rha (1 → 2)-*O*-β-d-Gal	Sadeghi et al. (2013)
*A. angulosum* Lour.		Diosgenin [**4**]		Azarkova et al. (1974)
*A. ascalonicum* L.	ASCALONICOSIDE A1 [**217**]	Furost-5(6)-ene-3β,22α-diol	26-*O*-α-l-Rha-(1 → 2)-*O*-β-d-Glc 1-*O*-β-d-Gal	Fattorusso et al. (2002)
	ASCALONICOSIDE A2 [**218**]	Furost-5(6)-ene-3β,22β-diol	26-*O*-α-l-Rha-(1 → 2)-*O*-β-d-Glc 1-*O*-β-d-Gal	Fattorusso et al. (2002)
	ASCALONICOSIDE B [**220**]	Furost-5(6),20(22)-diene-3β-ol	26-*O*-α-l-Rha-(1 → 2)-*O*-β-d-Glc 1-*O*-β-d-Gal	Fattorusso et al. (2002)
	ASCALONICOSIDE C [**221**]	(25*R*)-5α-furostane-3β,5α,6β,22,26-pentaol-2-one	26-*O*-β-d-Glc 3-*O*-α-l-Rha-(1 → 2)-*O*-β-d-Glc	Kang et al. (2007)
	ASCALONICOSIDE D [**222**]	(25*R*)-22-methoxy-5α-furostane-3β,5α,6β,26-tetrol-2-one	26-*O*-β-d-Glc 3-*O*-α-l-Rha-(1 → 2)-*O*-β-d-Glc	Kang et al. (2007)
	DICHOTOMIN [**223**]	(25*R*)-furost-5(6)-ene-3β,22α,26-triol	26-*O*-β-d-Glc 3-*O*-α-l-Rha-(1 → 4)-*O*-α-l-Rha-(1 → 4)-[α-l-Rha-(1 → 2)]-*O*-β-d-Glc	Kang et al. (2007)
	PARISAPONIN I [**224**]	(25*R*)-furost-5(6)-ene-3β,22,26-triol	26-*O*-β-d-Glc 3-*O*-α-l-Rha-(1 → 2)-[α-l-Ara-(1 → 4)]-*O*-β-d-Glc	Kang et al. (2007)
*A. atroviolaceum* Boiss.		Atroviolacegenin [**57**]		Zolfaghari et al. (2006)
	[**87**]	Agigenin [**34**]	3-*O*-β-d-Glc-(1 → 4)-*O*-β-d-Gal	Zolfaghari et al. (2006)
	ATROVIOLACEOSIDE [**108**]	Atroviolacegenin [**57**]	3-*O*-β-d-Glc-(1 → 4)-*O*-β-d-Gal	Zolfaghari et al. (2006)
	[**225**]	Furostane-2α,3β,6β,22α-tetrol	26-*O*-β-d-Glc 3-*O*-β-d-Glc-(1 → 4)-*O*-β-d-Gal	Zolfaghari et al. (2006)
	[**226**]	Furostane-2α,3β,6β,22α-tetrol	26-*O*-β-d-Glc 3-*O*-β-d-Xyl-(1 → 3)-*O*-β-d-Glc-(1 → 4)-*O*-β-d-Gal	Zolfaghari et al. (2006)
*A. cepa* L.		Diosgenin [**4**]		Kravets et al. (1990)
		(25*S*)-ruscogenin [**18**]		Kravets et al. (1986a, b)
		Cepagenin [**44**]		Kravets et al. (1987)
	[**132**]	Diosgenin [**4**]	3-*O*-α-l-Rha-(1 → 2)-*O*-α-l-Ara	Kravets et al. (1990)
	[**133**]	Diosgenin [**4**]	3-*O*-β-d-Gal-(1 → 4)-*O*-α-l-Rha-(1 → 2)-*O*-α-l-Ara	Kravets et al. (1990)
	[**143**]	Diosgenin [**4**]	3-*O*-β-d-Glc-(1 → 2)-[β-d-Glc-(1 → 3)]-*O*-β-d-Gal-(1 → 4)-*O*-α-l-Rha-(1 → 2)-*O*-α-l-Ara	Kintya and Degtyareva (1989)
	ALLIOSPIROSIDE A [**169**]	(25*S*)-ruscogenin [**18**]	1-*O*-α-l-Rha-(1 → 2)-*O*-α-l-Ara	Kravets et al. (1986a)
	ALLIOSPIROSIDE B [**170**]	(25*S*)-ruscogenin [**18**]	1-*O*-α-l-Rha-(1 → 2)-*O*-α-d-Gal	Kravets et al. (1986b)
	ALLIOSPIROSIDE C [**178**]	Cepagenin [**44**]	1-*O*-α-l-Rha-(1 → 2)-*O*-α-l-Ara	Kravets et al. (1987)
	ALLIOSPIROSIDE D [**179**]	Cepagenin [**44**]	1-*O*-α-l-Rha-(1 → 2)-*O*-α-d-Gal	Kravets et al. (1987)
	ALLIOFUROSIDE A [**227**]	(25*S*)-furost-5(6)-ene-1β,3β,22α,26-tetrol	26-*O*-β-d-Glc 1-*O*-α-l-Rha-(1 → 2)-*O*-α-l-Ara	Kravets et al. (1986a)
	CEPAROSIDE A [**228**]	(25*R*)-22-methoxy-furost-5(6)-ene-1β,3β,22α,26-tetrol	26-O-β-d-Glc 1-*O*-α-l-Rha-(1 → 2)-*O*-α-l-Ara	Yuan et al. (2008)
	CEPAROSIDE B [**229**]	(25*R*)-furost-5(6)-ene-1β,3β,22α,26-tetrol	26-O-β-d-Glc 1-*O*-α-l-Rha-(1 → 2)-*O*-α-l-Ara	Yuan et al. (2008)
	CEPAROSIDE C [**230**]	(25*R*)-furost-5(6),20(22)-diene-3β,26-diol	26-*O*-β-d-Glc 3-*O*-β-d-Glc-(1 → 4)-[α-l-Rha-(1 → 2)]-*O*-β-d-Gal	Yuan et al. (2009)
	CEPAROSIDE D [**231**]	(25*S*)-furost-5(6),20(22)-diene-3β,26-diol	26-*O*-β-d-Glc 3-*O*-β-d-Glc-(1 → 4)-[α-l-Rha-(1 → 2)]-*O*-β-d-Gal	Yuan et al. (2009)
	CEPOSIDE A1 [**209**]	(25*R*)-furost-5(6)-ene-1β,3β,22α,26-tetrol	26-*O*-α-l-Rha-(1 → 2)-*O*-β-d-Gal 1-*O*-β-d-Xyl	Lanzotti et al. (2012b)
	CEPOSIDE B [**232**]	(25*R*)-furost-5(6)-ene-1β,3β,22α,26-tetrol	26-*O*-α-l-Rha-(1 → 2)-*O*-β-d-Glc 1-*O*-β-d-Xyl	Lanzotti et al. (2012b)
	CEPOSIDE C1 [**211**]	(25*R*)-furost-5(6)-ene-1β,3β,22α,26-tetrol	26-*O*-α-l-Rha-(1 → 2)-*O*-β-d-Gal 1-*O*-β-d-Gal	Lanzotti et al. (2012b)
*A. cepa* L. var. *tropea*	TROPEOSIDE A1 [**213**]	Furost-5(6)-ene-3β,22α-diol	26-*O*-α-l-Rha 1-*O*-β-d-Gal	Corea et al. (2005)
	TROPEOSIDE A2 [**214**]	Furost-5(6)-ene-3β,22β-diol	26-*O*-α-l-Rha 1-*O*-β-d-Gal	Corea et al. (2005)
	TROPEOSIDE B1 [**215**]	Furost-5(6)-ene-3β,22α-diol	26-*O*-α-l-Rha 1-*O*-β-d-Xyl	Corea et al. (2005)
	TROPEOSIDE B2 [**216**]	Furost-5(6)-ene-3β,22β-diol	26-*O*-α-l-Rha 1-*O*-β-d-Xyl	Corea et al. (2005)
	ASCALONICOSIDES A1/A2 [**217**, **218**]			Corea et al. (2005)
	ASCALONICOSIDE B [**220**]			Corea et al. (2005)
*A. cepa* L. var. *aggregatum* (Aggregatum group)	ALLIOSPIROSIDE A [**169**]			Teshima et al. (2013)
	ALLIOSPIROSIDE B [**170**]			Teshima et al. (2013)
*A. cernuum* Roth.		Diosgenin [**4**]		Azarkova et al. (1983)
*A. chinense* G. Don		Tigogenin [**1**]		Matsuura et al. (1989b)
		Neotigogenin [**2**]	Sapogenins [1,2,6,9] obtained on acid hydrolysis of the crude saponin fraction	Matsuura et al. (1989b)
		Laxogenin [**6**]		Matsuura et al. (1989b)
		Gitogenin [**9**]		Matsuura et al. (1989b)
	[**65**, **110**]	(25*R*,*S*)-5α-spirostane-3β-ol [**1**, **2**]	3-*O*-β-d-Glc-(1 → 2)-[β-d-Glc-(1 → 3)]-*O*-β-d-Glc-(1 → 4)-*O*-β-d-Gal	Kuroda et al. (1995), Jiang et al. (1998)
	[**66**, **111**]	(25*R*,*S*)-5α-spirostane-3β-ol [**1**, **2**]	3-*O*-β-d-Glc-(1 → 2)-[β-d-Glc-(1 → 3)-(6-*O*-acetyl-β-d-Glc)]-(1 → 4)-*O*-β-d-Gal	Jiang et al. (1998)
	[**71**, **113**]	(25*R*,*S*)-5α-spirostane-2α,3β-diol [**9**, **10**]	3-*O*-β-d-Glc-(1 → 2)-*O*-β-d-Glc-(1 → 4)-*O*-β-d-Gal	Jiang et al. (1998)
	[**73**, **115**]	(25*R*,*S*)-5α-spirostane-2α,3β-diol [**9**, **10**]	3-*O*-β-d-Glc-(1 → 2)-[β-d-Glc-(1 → 3)]-*O*-β-d-Glc-(1 → 4)-*O*-β-d-Gal	Kuroda et al. (1995)
	NEOMACROSTEMONOSIDE D [**111**]	Neotigogenin [**2**]	3-*O*-β-d-Glc-(1 → 2)-[β-d-Glc-(1 → 3)-(6-*O*-acetyl-β-d-Glc)]-(1 → 4)-*O*-β-d-Gal	Jiang et al. (1999)
	CHINENOSIDE VI [**116**]	(25*S*)-5α-spirostane-3β,24β-diol [**16**]	3-*O*-α-l-Ara-(1 → 6)-*O*-β-d-Glc 24-*O*-β-d-Glc	Jiang et al. (1998)
	[**157**]	Laxogenin [**6**]	3-*O*-α-l-Ara-(1 → 6)-*O*-β-d-Glc	Kuroda et al. (1995), Peng et al. (1996b), Baba et al. (2000)
	[**159**]	Laxogenin [**6**]	3-*O*-(2-*O*-acetyl-α-l-Ara)-(1 → 6)-*O*-β-d-Glc	Kuroda et al. (1995)
	[**161**]	Laxogenin [**6**]	3-*O*-β-d-Xyl-(1 → 4)-[α-l-Ara-(1 → 6)]-*O*-β-d-Glc	Peng et al. (1995, 1996b); Baba et al. (2000)
	CHINENOSIDE I [**233**]	(25*R*)-5α-furostane-3β,22,26-triol-6-one	26-*O*-β-d-Glc 3-*O*-β-d-Xyl-(1 → 4)-[α-l-Ara-(1 → 6)]-*O*-β-d-Glc	Matsuura et al. (1989b)
	CHINENOSIDE II [**234**]	(25*R*)-5α-furost-20(22)-ene-3β,26-diol-6-one	26-*O*-β-d-Glc 3-*O*-β-d-Xyl-(1 → 4)-[α-l-Ara-(1 → 6)]-*O*-β-d-Glc	Peng et al. (1996b)
	CHINENOSIDE III [**235**]	(25*R*)-5α-furost-20(22)-ene-3β,26-diol-6-one	26-*O*-β-d-Glc 3-*O*-α-l-Ara-(1 → 6)-*O*-β-d-Glc	Peng et al. (1996b)
	CHINENOSIDE IV [**236**]	(25*R*)-23-methoxy-5α-furost-20(22)-ene-3β,26-diol-6-one	26-*O*-β-d-Glc 3-*O*-β-d-Xyl-(1 → 4)-[α-l-Ara-(1 → 6)]-*O*-β-d-Glc	Peng et al. (1996c)
	CHINENOSIDE V [**237**]	(25*R*)-23-methoxy-5α-furost-20(22)-ene-3β,26-diol-6-one	26-*O*-β-d-Glc 3-*O*-α-l-Ara-(1 → 6)-*O*-β-d-Glc	Peng et al. (1996c)
*A. cyrillii* Ten.	F-GITONIN [**72**]	Gitogenin [**9**]	3-*O*-β-d-Glc-(1 → 2)-[β-d-Xyl-(1 → 3)]-*O*-β-d-Glc-(1 → 4)-*O*-β-d-Gal	Tolkacheva et al. (2012)
	[**75**]	Gitogenin [**9**]	3-*O*-β-d-Glc-(1 → 2)-[4-*O*-(*S*)-3-hydroxy-3-methylglutaryl-*O*-β-d-Xyl-(1 → 3)]-*O*-β-d-Glc-(1 → 4)-*O*-β-d-Gal	Tolkacheva et al. (2012)
*A. elburzense* Wendelbo		Agapanthagenin [**31**]		Barile et al. (2004)
		Alliogenin [**49**]		Barile et al. (2004)
	[**85**]	Agapanthagenin [**31**]	3-*O*-β-d-Glc	Barile et al. (2004)
	[**69**]	Gitogenin [**9**]	3-*O*-β-d-Glc-(1 → 4)-*O*-β-d-Glc	Barile et al. (2004)
	[**106**]	Alliogenin [**49**]	3-*O*-β-d-Glc	Barile et al. (2004)
	ELBURZENSOSIDE A1 [**238**]	Furostane-2α,3β,5α,6β,22α-pentaol	26-*O*-β-d-Glc 3-*O*-β-d-Glc	Barile et al. (2004)
	ELBURZENSOSIDE A2 [**239**]	Furostane-2α,3β,5α,6β,22β-pentaol	26-*O*-β-d-Glc 3-*O*-β-d-Glc	Barile et al. (2004)
	ELBURZENSOSIDE B1 [**240**]	Furostane-2α,3β,5α,6β,22α-pentaol	26-*O*-β-d-Glc 3-*O*-β-d-Glc-(1 → 4)-*O*-β-d-Glc	Barile et al. (2004)
	ELBURZENSOSIDE B2 [**241**]	Furostane-2α,3β,5α,6β,22β-pentaol	26-*O*-β-d-Glc 3-*O*-β-d-Glc-(1 → 4)-*O*-β-d-Glc	Barile et al. (2004)
	ELBURZENSOSIDE C1 [**242**]	Furostane-2α,3β,5α,22α-tetrol	26-*O*-β-d-Glc 3-*O*-β-d-Glc	Barile et al. (2004)
	ELBURZENSOSIDE C2 [**243**]	Furostane-2α,3β,5α,22β-tetrol	26-*O*-β-d-Glc 3-*O*-β-d-Glc	Barile et al. (2004)
	ELBURZENSOSIDE D1 [**244**]	Furostane-2α,3β,5α,22α-tetrol	26-*O*-β-d-Glc 3-*O*-β-d-Xyl-(1 → 3)-*O*-β-d-Glc-(1 → 4)-*O*-β-d-Gal	Barile et al. (2004)
	ELBURZENSOSIDE D2 [**245**]	Furostane-2α,3β,5α,22β-tetrol	26-*O*-β-d-Glc 3-*O*-β-d-Xyl-(1 → 3)-*O*-β-d-Glc-(1 → 4)-*O*-β-d-Gal	Barile et al. (2004)
*A. erubescens* C. Koh.		β-Chlorogenin [**12**]		Chincharadze et al. (1979)
	ERUBOSIDE B [**79**]	β-Chlorogenin [**12**]	3-*O*-β-Glc-(1 → 3)-[β-d-Glc-(1 → 2)]-*O*-β-d-Glc-(1 → 4)-*O*-β-d-Gal	Chincharadze et al. (1979)
*A. fistulosum* L.		Yuccagenin [**19**]		Kim et al. (1991)
		Tigogenin [**1**]		Lai et al. (2012)
		Gitogenin [**9**]		Lai et al. (2012)
		(25*R*)-19-norspirosta-1,3,5(10)-triene-4-methyl-2-ol [**246**]		Lai et al. (2012)
		(25*R*)-spirost-1(2),4(5)-diene-2,6-diol-3-one [**247**]	Sapogenins [1, 9, 246, 247, 248, 249] obtained from the acid hydrolysis product of the whole glycoside mixture of Welsh onion seeds	Lai et al. (2012)
		(25*R*)-spirost-1(2),4(5)-diene-2-ol-3-one [**248**]		Lai et al. (2012)
		(25*R*)-spirost-4(5)-ene-2-ol-3-one [**249**]		Lai et al. (2012)
	DIOSCIN [**135**]			Jung et al. (1993)
	[**141**]	Diosgenin [**4**]	3-*O*-α-l-Rha-(1 → 4)-*O*-α-l-Rha-(1 → 4)-[α-l-Rha-(1 → 2)]-*O*-β-d-Glc	Jung et al. (1993)
	FISTULOSIDE A [**148**]	Yuccagenin [**19**]	3-*O*-α-l-Rha-(1 → 2)-*O*-β-d-Gal	Do et al. (1992)
	FISTULOSIDE B [**149**]	Yuccagenin [**19**]	3-*O*-α-l-Rha-(1 → 2)-[β-d-Glc-(1 → 3)]-*O*-β-d-Gal	Do et al. (1992)
	FISTULOSIDE C [**150**]	Yuccagenin [**19**]	3-*O*-β-d-Glc-(1 → 3)-[β-d-Glc-(1 → 4)]-*O*-β-d-Gal	Do et al. (1992)
	FISTULOSAPONIN A [**250**]	(25*R*)-furost-5(6),20(22)-diene-3β,26-diol-2-one	26-*O*-β-d-Glc 3-*O*-α-l-Rha-(1 → 2)-[α-l-Rha-(1 → 4)]-*O*-β-d-Glc	Lai et al. (2010)
	FISTULOSAPONIN B [**251**]	(25*R*)-furost-5(6)-ene-3β,22α,26-triol-2-one	26-*O*-β-d-Glc 3-*O*-α-l-Rha-(1 → 2)-[α-l-Rha-(1 → 4)]-*O*-β-d-Glc	Lai et al. (2010)
	FISTULOSAPONIN C [**252**]	(25*R*)-furost-5(6)-ene-3α,22α,26-triol-2-one	26-*O*-β-d-Glc 3-*O*-α-l-Rha-(1 → 4)-*O*-α-l-Rha-(1 → 2)-*O*-β-d-Glc	Lai et al. (2010)
	FISTULOSAPONIN D [**253**]	(25*R*)-furost-5(6)-ene-3β,22α,26-triol-2-one	26-*O*-β-d-Glc 3-*O*-β-d-Glc-(1 → 2)-*O*-β-d-Glc-(1 → 2)-*O*-β-d-Glc	Lai et al. (2010)
	FISTULOSAPONIN E [**254**]	(25*R*)-furost-5(6),20(22)-diene-2α,3β,26-triol	26-*O*-β-d-Glc 3-*O*-β-d-Glc-(1 → 2)-*O*-β-d-Glc-(1 → 2)-*O*-β-d-Glc	Lai et al. (2010)
	FISTULOSAPONIN F [**255**]	(25*R*)-furost-5(6)-ene-2α,3β,22α,26-tetrol	26-*O*-β-d-Glc 3-*O*-β-d-Glc-(1 → 2)-*O*-β-d-Glc-(1 → 2)-*O*-β-d-Glc	Lai et al. (2010)
	PROTOGRACILLIN [**256**]	(25*R*)-furost-5(6)-ene-3β,22α,26-triol	26-*O*-β-d-Glc 3-*O*-α-l-Rha-(1 → 2)-[β-d-Glc-(1 → 3)]-*O*-β-d-Glc	Lai et al. (2010)
	[**257**]	(25*R*)-furost-5(6)-ene-3β,22α,26-triol	26-*O*-β-d-Glc 3-*O*-α-l-Rha-(1 → 2)-[α-l-Rha-(1 → 4)]-*O*-β-d-Glc	Lai et al. (2010)
	DICHOTOMIN [**223**]			Lai et al. (2010)
*A. flavum* L.	[**137**]	Diosgenin [**4**]	3-*O*-α-l-Rha-(1 → 4)-[β-d-Glc-(1 → 2)]-*O*-β-d-Glc	Rezgui et al. (2014)
	[**153**]	Yuccagenin [**19**]	3-*O*-β-d-Xyl-(1 → 3)-[β-d-Gal-(1 → 2)]-*O*-β-d-Gal-(1 → 4)-*O*-β-d-Gal	Rezgui et al. (2014)
	[**154**]	Yuccagenin [**19**]	3-*O*-β-d-Xyl-(1 → 3)-[β-d-Glc-(1 → 2)]-*O*-β-d-Gal-(1 → 4)-*O*-β-d-Gal	Rezgui et al. (2014)
*A. fuscoviolaceum* L.		Diosgenin [**4**]		Kravets et al. (1990)
*A. giganteum* Regel		Diosgenin [**4**]		Kravets et al. (1990)
		β-Chlorogenin [**12**]		Kelginbaev et al. (1974)
		Yuccagenin [**19**]		Kelginbaev et al. (1974)
		Gantogenin [**33**]		Kelginbaev et al. (1975)
		Agigenin [**34**]		Kelginbaev et al. (1974)
		Neoagigenin [**36**]		Kelginbaev et al. (1973, 1974)
		Alliogenin [**49**]		Khristulas et al. (1970), Gorovits et al. (1971)
		Luvigenin [**59**]		Kravets et al. (1990)
	AGINOSIDE [**93**]			Kelginbaev et al. (1976), Kawashima et al. (1991a)
	[**98**]	Agigenin [**34**]	3-*O*-β-d-Glc-(1 → 2)-[4-*O*-(*S*)-3-hydroxy-3-methylglutaryl-β-d-Xyl-(1 → 3)]-*O*-β-d-Glc-(1 → 4)-*O*-β-d-Gal	Mimaki et al. (1994)
	[**105**]	Alliogenin [**49**]	2-*O*-β-d-Glc	Sashida et al. (1991)
	[**106**]	Alliogenin [**49**]	3-*O*-β-d-Glc	Gorovits et al. (1971)
	[**184**]	3-*O*-acetyl-alliogenin [**51**]	2-*O*-β-d-Glc	Sashida et al. (1991)
	[**186**]	Karatavigenin [**52**]	2-*O*-β-d-Glc	Sashida et al. (1991)
	[**191**]	(24*S*,25*R*)-5α-spirostane-2α,3β,5α,6β,24-pentaol [**60**]	24-*O*-β-d-Glc	Kawashima et al. (1991a)
	[**193**]	3-*O*-acetyl-(24*S*,25*S*)-5α-spirostane-2α,3β, 5α,6β,24-pentaol [**62**]	2-*O*-β-d-Glc	Mimaki et al. (1994)
	[**258**]	(25*R*)-22-methoxy-5α-furostane-2α,3β,6β,22ξ,26-pentaol	26-*O*-β-d-Glc 3-*O*-β-Glc-(1 → 2)-[β-d-Xyl-(1 → 3)]-*O*-β-d-Glc-(1 → 4)-*O*-β-d-Gal	Mimaki et al. (1994)
	[**259**]	(25*R*)-3-*O*-benzoyl-22-methoxy-5α-furostane-2α,3β,5α,6β,22ξ,26-hexol	26-*O*-β-d-Glc 2-*O*-β-d-Glc	Mimaki et al. (1994)
	[**260**]	(25*R*)-3-*O*-acetyl-22-methoxy-5α-furostane-2α,3β,5α,6β,22ξ,26-hexol	26-*O*-β-d-Glc 2-*O*-β-d-Glc	Mimaki et al. (1994)
*A. gramineum* C. Koch.		Diosgenin [**4**]		Kravets et al. (1990)
		β-Chlorogenin [**12**]		Kravets et al. (1990)
		Agigenin [**34**]		Kravets et al. (1990)
	ERUBOSIDE B [**79**]			Kravets et al. (1990)
*A. hirtifolium* Boiss.	[**69**]	Gitogenin [**9**]	3-*O*-β-d-Glc-(1 → 4)-*O*-β-d-Glc	Barile et al. (2005)
	[**85**]	Agapanthagenin [**31**]	3-*O*-β-d-Glc	Barile et al. (2005)
	HIRTIFOLIOSIDE D [**92**]	Agigenin [**34**]	3-*O*-β-d-Xyl-(1 → 3)-*O*-β-d-Glc-(1 → 4)-*O*-β-d-Gal	Barile et al. (2005)
	[**106**]	Alliogenin [**49**]	3-*O*-β-d-Glc	Barile et al. (2005)
	HIRTIFOLIOSIDE A1 [**261**]	Furostane-2α,3β,22α-triol	26-*O*-β-d-Glc 3-*O*-β-d-Xyl-(1 → 3)-*O*-β-d-Glc-(1 → 4)-*O*-β-d-Gal	Barile et al. (2005)
	HIRTIFOLIOSIDE A2 [**262**]	Furostane-2α,3β,22β-triol	26-*O*-β-d-Glc 3-*O*-β-d-Xyl-(1 → 3)-*O*-β-d-Glc-(1 → 4)-*O*-β-d-Gal	Barile et al. (2005)
	HIRTIFOLIOSIDE B [**263**]	Furost-20(22)-ene-2α,3β-diol	26-*O*-β-d-Glc 3-*O*-β-d-Xyl-(1 → 3)-*O*-β-d-Glc-(1 → 4)-*O*-β-d-Gal	Barile et al. (2005)
	HIRTIFOLIOSIDE C1 [**264**]	Furostane-2α,3β,22α-triol	26-*O*-β-d-Glc	Barile et al. (2005)
	HIRTIFOLIOSIDE C2 [**265**]	Furostane-2α,3β,22β-triol	26-*O*-β-d-Glc	Barile et al. (2005)
*A. jesdianum* Boiss.	F-GITONIN [**72**]			Mimaki et al. (1999c)
	[**86**]	Gantogenin [**33**]	3-*O*-β-d-Glc-(1 → 2)-[β-d-Xyl-(1 → 3)]-*O*-β-d-Glc-(1 → 4)-*O*-β-d-Gal	Mimaki et al. (1999c)
	[**266**]	Alliosterol [**196**]	1-*O*-β-d-Glc 16-*O*-β-d-Glc	Mimaki et al. (1999c)
	[**267**]	Alliosterol [**196**]	1-*O*-α-l-Rha 16-*O*-β-d-Glc	Mimaki et al. (1999c)
*A. karataviense* Regel		Diosgenin [**4**]		Gorovits et al. (1973)
		Yuccagenin [**19**]		Gorovits et al. (1973)
		Karatavigenin c [**45**]		Vollerner et al. (1983b)
		Alliogenin [**49**]		Gorovits et al. (1973), Mimaki et al. (1999c)
		Karatavigenin [**52**]		Gorovits et al. (1973)
		Karatavigenin B [**53**]		Khristulas et al. (1974)
	[**105**]	Alliogenin [**49**]	2-*O*-β-d-Glc	Mimaki et al. (1999c)
	[**106**]	Alliogenin [**49**]	3-*O*-β-d-Glc	Gorovits et al. (1973)
	[**107**]	Alliogenin [**49**]	3-*O*-β-d-Glc-(1 → 2)-[β-d-Xyl-(1 → 3)]-*O*-β-d--d-Glc-(1 → 4)-*O*-β-d-Gal	Mimaki et al. (1999c)
	KARATAVIOSIDE A [**151**]			Vollerner et al. (1978), Mimaki et al. (1999c)
	KARATAVIOSIDE B [**152**]	Yuccagenin [**19**]	3-*O*-β-d-Glc-(1 → 2)-[4-*O*-β-hydroxy-β-methylglutaryl-β-d-Xyl-(1 → 3)]-*O*-β-d-Glc-(1 → 4)-*O*-β-d-Gal	Vollerner et al. (1983a)
	KARATAVIOSIDE E [**180**]	Karatavigenin C [**45**]	3-*O*-β-d-Xyl-(1 → 3)-[β-d-Glc-(1 → 2)]-*O*-β-d-Glc-(1 → 4)-*O*-β-d-Gal	Vollerner et al. (1984)
	KARATAVIOSIDE F [**181**]	Karatavigenin C [**45**]	3-*O*-β-d-Xyl-(1 → 3)-[β-d-Glc-(1 → 2)]-*O*-β-d-Glc-(1 → 4)-*O*-β-d-Gal 24-*O*-β-d-Glc	Vollerner et al. (1984)
	[**184**]	3-*O*-acetyl-alliogenin [**51**]	2-*O*-β-d-Glc	
	[**185**]	3-*O*-(2-hydroxybutyryl)-alliogenin [**54**]	2-*O*-β-d-Glc	Mimaki et al. (1999c)
	[**186**]	Karatavigenin [**52**]	2-*O*-β-d-Glc	Mimaki et al. (1999c)
	[**187**]	Karatavigenin B [**53**]	3-*O*-β-d-Glc	Khristulas et al. (1974)
	[**192**]	(24*S*,25*S*)-5α-spirostane-2α,3β,5α,6β,24-pentaol [**61**]	2-*O*-β-d-Glc 24-*O*-β-d-Glc-(1 → 2)-*O*-β-d-Glc	Mimaki et al. (1999c)
	[**194**]	3-*O*-benzoyl-(24*S*,25*S*)-5α-spirostane-2α,3β,5α,6β,24-pentaol [**63**]	2-*O*-β-d-Glc	Mimaki et al. (1999c)
	[**195**]	3-*O*-benzoyl-(24*S*,25*S*)-5α-spirostane-2α,3β,5α,6β,24-pentaol [**63**]	2-*O*-β-d-Glc 24-*O*-β-d-Glc	Mimaki et al. (1999c)
	KARATAVIOSIDE C [**268**]	(25*R*)-furost-5(6)-ene-2α,3β,22α,26-tetrol	26-*O*-β-d-Glc 3-*O*-β-d-Glc-(1 → 2)-[β-d-Xyl-(1 → 3)]-*O*-β-d-Glc-(1 → 4)-*O*-β-d-Gal	Vollerner et al. (1980)
	[**269**]	(25*R*)-22-methoxy-5α-furostane-2α,3β,5,6β,22ξ-pentaol	26-*O*-β-d-Glc 2-*O*-β-d-Glc	Mimaki et al. (1999c)
*A. leucanthum* C. Koch	ERUBOSIDE B [**79**]			Mskhiladze et al. (2008b)
	[**80**]	β-Chlorogenin [**12**]	3-*O*-β-d-Glc-(1 → 2)-[β-d-Xyl-(1 → 3)]-*O*-β-d-Glc-(1 → 4)-*O*-β-d-Gal	Mskhiladze et al. (2008b)
	[**81**]	β-Chlorogenin [**12**]	3-*O*-β-d-Glc-(1 → 3)-*O*-β-d-Glc-(1 → 2)-[β-d-Glc-(1 → 3)]-*O*-β-d-Glc-(1 → 4)-*O*-β-d-Gal	Mskhiladze et al. (2008b)
	[**91**]	Agigenin [**34**]	3-*O*-β-d-Glc-(1 → 2)-*O*-β-d-Glc-(1 → 4)-*O*-β-d-Gal	Mskhiladze et al. (2008b)
	AGINOSIDE [**93**]			Mskhiladze et al. (2008b)
	YAYOISAPONIN C [**95**]			Mskhiladze et al. (2008b)
	LEUCOSPIROSIDE A [**97**]	Agigenin [**34**]	3-*O*-β-d-Glc-(1 → 3)-*O*-β-d-Glc-(1 → 2)-[β-d-Glc-(1 → 3)]-*O*-β-d-Glc-(1 → 4)-*O*-β-d-Gal	Mskhiladze et al. (2008b)
*A. macleanii* Baker	[**67**]	Tigogenin [**1**]	3-*O*-α-l-Rha-(1 → 2)-*O*-β-d-Xyl-(1 → 2)-[β-d-Xyl-(1 → 3)]-*O*-β-d-Glc-(1 → 4)-*O*-β-d-Gal	Inoue et al. (1995)
	AGINOSIDE [**93**]			Inoue et al. (1995)
	[**98**]	Agigenin [**34**]	3-*O*-β-d-Glc-(1 → 2)-[4-*O*-(*S*)-3-hydroxy-3-methylglutaryl-β-d-Xyl-(1 → 3)]-*O*-β-d-Glc-(1 → 4)-*O*-β-d-Gal	Inoue et al. (1995)
	[**100**]	Agigenin [**34**]	3-*O*-β-d-Glc-(1 → 2)-[4-*O*-benzoyl-β-d-Xyl-(1 → 3)]-*O*-β-d-Glc-(1 → 4)-*O*-β-d-Gal	Inoue et al. (1995)
	[**105**]	Alliogenin [**49**]	2-*O*-β-d-Glc	Inoue et al. (1995)
	[**186**]	Karatavigenin [**52**]	2-*O*-β-d-Glc	Inoue et al. (1995)
	[**270**]	Alliosterol [**196**]	1-*O*-α-l-Rha 3-*O*-α-l-Rha 16-*O*-β-d-Glc	Inoue et al. (1995)
*A. macrostemon* Bunge (*A. grayi* Regel)		Tigogenin [**1**]		Okanishi et al.(1975), He et al. (2002)
		Smilagenin [**3**]		Okanishi et al.(1975)
		Gitogenin [**9**]		Okanishi et al.(1975)
	MACROSTEMONOSIDE A [**65**]	Tigogenin [**1**]	3-*O*-β-d-Glc-(1 → 2)-[β-d-Glc-(1 → 3)]-*O*-β-d-Glc-(1 → 4)-*O*-β-d-Gal	Peng et al. (1992)
	MACROSTEMONOSIDE D [**66**]	Tigogenin [**1**]	3-*O*-β-d-Glc-(1 → 2)-[β-d-Glc-(1 → 3)-(6-*O*-acetyl-β-d-Glc)]-(1 → 4)-*O*-β-d-Gal	Peng et al.(1992)
	[**129**]	(25*R*)-5β-spirostane-3β,12β-diol [**15**]	3-*O*-β-d-Glc-(1 → 2)-*O*-β-d-Gal	Cheng et al. (2013)
	[**172**]	5β-Spirost-25(27)-ene-2β,3β-diol [**21**]	3-*O*-β-d-Glc-(1 → 2)-*O*-β-d-Gal	He et al. (2002), Cheng et al. (2013)
	MACROSTEMONOSIDE S [**173**]	5β-Spirost-25(27)-ene-3β,12β-diol [**22**]	3-*O*-β-d-Glc-(1 → 2)-*O*-β-d-Gal	Cheng et al. (2013)
	MACROSTEMONOSIDE B [**271**]	(25*R*)-5β-furostane-3β,22,26-triol	26-*O*-β-d-Glc 3-*O*-β-d-Glc-(1 → 2)-[β-d-Glc-(1 → 3)]-*O*-β-d-Glc-(1 → 4)-*O*-β-d-Gal	Chen et al. (2007)
	MACROSTEMONOSIDE E [**272**]	5α-Furost-20(22)-ene-3β,26-diol	26-*O*-β-d-Glc 3-*O*-β-d-Glc-(1 → 2)-[β-d-Glc-(1 → 3)]-*O*-β-d-Glc-(1 → 4)-*O*-β-d-Gal	Peng et al. (1993)
	MACROSTEMONOSIDE F [**273**]	5β-Furost-20(22)-ene-3β,26-diol	26-*O*-β-d-Glc 3-*O*-β-d-Glc-(1 → 2)-*O*-β-d-Gal	Peng et al.(1993)
	MACROSTEMONOSIDE G [**274**]	5β-Furost-25(27)-ene-3β,12β,22,26-tetrol	26-*O*-β-d-Glc 3-*O*-β-d-Glc-(1 → 2)-*O*-β-d-Gal	Peng et al. (1995)
	MACROSTEMONOSIDE H [**275**]	22-Methoxy-5β-furost-25(27)-ene-3β,12β,22,26-tetrol	26-*O*-β-d-Glc 3-*O*-β-d-Glc-(1 → 2)-*O*-β-d-Gal	Peng et al. (1995)
	MACROSTEMONOSIDE I [**276**]	5β-Furost-25(27)-ene-3β,22,26-triol-12-one	26-*O*-β-d-Glc 3-*O*-β-d-Glc-(1 → 2)-*O*-β-d-Gal	Peng et al. (1995)
	MACROSTEMONOSIDE J [**277**]	(25*R*)-5β-furostane-2β,3β,22,26-tetrol	26-*O*-β-d-Glc 3-*O*-β-d-Glc-(1 → 2)-*O*-β-d-Gal	Peng et al. (1994)
	MACROSTEMONOSIDE K [**278**]	(25*R*)-22-methoxy-5β-furostane-2β,3β,22,26-tetrol	26-*O*-β-d-Glc 3-*O*-β-d-Glc-(1 → 2)-*O*-β-d-Gal	Peng et al. (1994)
	MACROSTEMONOSIDE L [**279**]	(25*R*)-5β-furost-20(22)-ene-2β,3β,26-triol	26-*O*-β-d-Glc 3-*O*-β-d-Glc-(1 → 2)-*O*-β-d-Gal	Peng et al. (1994)
	[**280**]	5β-Furost-25(27)-ene-1β,3β,3β,22α,26-pentaol	26-*O*-β-d-Glc 3-*O*-β-d-Gal	He et al. (2002)
	MACROSTEMONOSIDE M [**281**]	(25*R*)-5β-furostane-1β,2β,3β,6α,22-pentaol	26-*O*-β-d-Glc	Chen et al. (2006)
	MACROSTEMONOSIDE N [**282**]	5β-Furost-25(27)-ene-1β,2β,3β,6α,22-pentaol	26-*O*-β-d-Glc	Chen et al. (2006)
	MACROSTEMONOSIDE *O* [**283**]	5β-Furost-25(27)-ene-3β,22,26-triol	26-*O*-β-d-Glc 3-*O*-β-d-Glc-(1 → 2)-*O*-β-d-Gal	Chen et al. (2007)
	MACROSTEMONOSIDE P [**284**]	(25*R*)-5β-furostane-1β,3β,22,26-tetrol	26-*O*-β-d-Glc 3-*O*-β-d-Glc-(1 → 2)-*O*-β-d-Gal	Chen et al. (2007)
	MACROSTEMONOSIDE Q [**285**]	(25*R*)-5β-furostane-1α,2β,3β,22,26-pentaol	26-*O*-β-d-Glc 3-*O*-β-d-Glc-(1 → 2)-*O*-β-d-Gal	Chen et al. (2007)
	MACROSTEMONOSIDE R [**286**]	(25*R*)-5β-furostane-2α,3β,22,26-tetrol	26-*O*-β-d-Glc 3-*O*-β-d-Glc-(1 → 2)-[β-d-Glc-(1 → 3)]-*O*-β-d-Glc-(1 → 4)-*O*-β-d-Gal	Chen et al. (2007)
	[**287**]	(25*R*)-furostane-3β,22,26β-triol	26-*O*-β-d-Glc 3-*O*-β-d-Glc-(1 → 2)-*O*-β-d-Gal	Chen et al. (2006)
	[**288**]	(25*S*)-furostane-3β,22,26β-triol	26-*O*-β-d-Glc 3-*O*-β-d-Glc-(1 → 2)-*O*-β-d-Gal	Chen et al. (2006)
	[**289**]	(25*R*)-5α-furostane-3β,12β,22,26-tetrol	26-*O*-β-d-Glc 3-*O*-β-d-Glc-(1 → 2)-[β-d-Glc-(1 → 3)]-*O*-β-d-Glc-(1 → 4)-*O*-β-d-Gal	Chen et al. (2010)
	[**290**]	(25*R*)-5α-furostane-3β,12α,22,26-tetrol	26-*O*-β-d-bGlc 3-*O*-β-d-Glc-(1 → 2)-[β-d-Glc-(1 → 3)]-*O*-β-d-Glc-(1 → 4)-*O*-β-d-Gal	Chen et al. (2010)
	[**291**]	(25*R*)-5β-furostane-3β,12α,22,26-tetrol	26-*O*-β-d-Glc 3-*O*-β-d-Glc-(1 → 2)-*O*-β-d-Gal	Chen et al. (2010)
	[**292**]	5α-Furost-25(27)-ene-3β,12β,22,26-tetrol	26-*O*-β-d-Glc 3-*O*-β-d-Glc-(1 → 2)-[β-d-Glc-(1 → 3)]-*O*-β-d-Glc-(1 → 4)-*O*-β-d-Gal	Chen et al. (2009)
	[**293**]	5β-Furost-20(22),25(27)-diene-3β,12β,26-triol	26-*O*-β-d-Glc 3-*O*-β-d-Glc-(1 → 2)-*O*-β-d-Gal	Chen et al. (2009)
	[**294**]	5β-Furostane-3β,12α,22,26-tetrol	26-*O*-β-d-Glc 3-*O*-β-d-Glc-(1 → 2)-[β-d-Glc-(1 → 3)]-*O*-β-d-Glc-(1 → 2)-*O*-β-d-Gal	Ou et al. (2012)
*A. minutiflorum* Regel		Neoagigenin [**36**]		Barile et al. (2007)
		Alliogenin [**49**]		Barile et al. (2007)
	MINUTOSIDE B [**119**]	Neoagigenin [**36**]	3-*O*-β-d-Xyl-(1 → 3)-*O*-β-d-Glc-(1 → 4)-*O*-β-d-Gal	Barile et al. (2007)
	MINUTOSIDE A [**295**]	(25*R*)-furostane-2α,3β,6β,22α,26-pentaol	26-*O*-β-d-Glc 3-*O*-β-d-Xyl-(1 → 3)-*O*-β-d-Glc-(1 → 4)-*O*-β-d-Gal	Barile et al. (2007)
	MINUTOSIDE C [**296**]	(25*R*)-furostane-2α,3β,5α,6β,22α,26-hexol	26-*O*-β-d-Glc 3-*O*-β-d-Xyl-(1 → 3)-*O*-β-d-Glc-(1 → 4)-*O*-β-d-Gal	Barile et al. (2007)
*A. narcissiflorum* Vill.	TRILLIN (ALLIUMOSIDE A) [**130**]	Diosgenin [**4**]	3-*O*-β-d-Glc	Krokhmalyuk and Kintya (1976b)
	DELTONIN [**134**]	Diosgenin [**4**]	3-*O*-α-l-Rha-(1 → 2)-[β-d-Glc-(1 → 4)]-*O*-β-d-Glc	Mimaki et al. (1996)
	[**139**]	Diosgenin [**4**]	3-*O*-α-l-Rha-(1 → 2)-[β-d-Xyl-(1 → 4)]-*O*-β-d-Glc	Mimaki et al. (1996)
	[**141**]	Diosgenin [**4**]	3-*O*-α-l-Rha-(1 → 4)-*O*-α-l-Rha-(1 → 4)-[α-l-Rha-(1 → 2)]-*O*-β-d-Glc	Mimaki et al. (1996)
	ALLIUMOSIDE B [**297**]	(25*R*)-furost-5(6)-ene-3β,22α,26-triol	26-*O*-β-d-Glc 3-*O*-β-d-Glc-(1 → 3)-*O*-β-d-Glc-(1 → 6)-*O*-β-d-Glc	Krokhmalyuk and Kintya (1976b)
	ALLIUMOSIDE C [**298**]	(25*R*)-furostane-3β,22α,26-triol	26-*O*-β-d-Glc 3-*O*-α-l-Rha-(1 → 4)-*O*-α-l-Rha-(1 → 4)-*O*-α-l-Rha-(1 → 6)-*O*-β-d-Gal-(1 → 6)-*O*-β-d-Glc	Lazurevski et al. (1975)
	ALLIUMOSIDE D [**299**]	(25*S*)-furost-5(6)-ene-3β,22α,26-triol	26-*O*-β-d-Glc 3-*O*-α-l-Rha-(1 → 4)-*O*-α-l-Rha-(1 → 6)-*O*-β-d-Glc-(1 → 2)-[β-d-Glc-(1 → 3)]-*O*-β-d-Glc	Krokhmalyuk and Kintya (1976a)
	ALLIUMOSIDE E [**300**]	(25*S*)-furost-5(6)-ene-3β,22α,26-triol	26-*O*-β-d-Glc 3-*O*-β-d-Glc-(1 → 4)-*O*-α-l-Rha-(1 → 4)-*O*-α-l-Rha-(1 → 6)-*O*-β-d-Glc-(1 → 2)-[β-d-Glc-(1 → 3)]-*O*-β-d-Glc	Krokhmalyuk and Kintya (1976a)
	[**301**]	(25*R*)-22-methoxy-furost-5(6)-ene-3β,22ξ,26-triol	26-*O*-β-d-Glc 3-*O*-α-l-Rha-(1 → 2)-*O*-β-d-Glc	Mimaki et al. (1996)
	[**302**]	(25*R*)-22-methoxy-furost-5(6)-ene-3β,22ξ,26-triol	26-*O*-β-d-Glc 3-*O*-α-l-Rha-(1 → 2)-*O*-[β-d-Glc-(1 → 4)]-*O*-β-d-Glc	Mimaki et al. (1996)
*A. nigrum* L.	NIGROSIDES A1/A2 [**89**, **117**]	(25*R,S*)-5α-spirostane-2α,3β,6β-triol [**34**, **36**]	3-*O*-α-l-Rha-(1 → 2)-*O*-β-d-Glc	Jabrane et al. (2011)
	NIGROSIDES B1/B2 [**88**, **118**]	(25*R,S*)-5α-spirostane-2α,3β,6β-triol [**34**, **36**]	2-*O*-β-d-Glc 3-*O*-β-d-Gal	Jabrane et al. (2011)
	AGINOSIDE [**93**]			Mostafa et al. (2013)
	AGINOSIDE/TUROSIDE A [**93**, **122**]	(25*R*,*S*)-5α-spirostane-2α,3β,6β-triol [**34**, **36**]	3-*O*-β-d-Xyl-(1 → 3)-[β-d-Glc-(1 → 2)]-*O*-β-d-Glc-(1 → 4)-*O*-β-d-Gal	Jabrane et al. (2011)
	[**98**, **124**]	(25*R*,*S*)-5α-spirostane-2α,3β,6β-triol [**34**, **36**]	3-*O*-β-d-Glc-(1 → 2)-[4-*O*-(*S*)-3-hydroxy-3-methylglutaryl-β-d-Xyl-(1 → 3)]-*O*-β-d-Glc-(1 → 4)-*O*-β-d-Gal	Jabrane et al. (2011)
	NIGROSIDE C (SCHUBERTOSIDE D) [**303**]	Alliosterol [**196**]	1-O-α-l-Rha 16-O-α-l-Rha-(1 → 3)-*O*-β-d-Gal	Jabrane et al. (2011)
	NIGROSIDE D [**304**]	Alliosterol [**196**]	16-O-α-l-Rha-(1 → 3)-*O*-β-d-Gal	Jabrane et al. (2011)
*A. nutans* L.		Diosgenin [**4**]		Azarkova et al. (1983)
	[**138**]	Diosgenin [**4**]	3-*O*-α-l-Rha-(1 → 2)-[β-d-Glc-(1 → 4)]-*O*-β-d-Gal	Akhov et al. (1999)
	[**147**]	Ruscogenin [**17**]	1-*O*-β-d-Gal	Akhov et al. (1999)
	NOLINOFUROSIDE D [**305**]	(25S)-furost-5(6)-ene-1β,3β,22α,26-tetrol	26-*O*-β-d-Glc 1-*O*-β-d-Gal	Akhov et al. (1999)
	DELTOSIDE [**306**]	(25*R*)-furost-5(6)-ene-3β,22α,26-triol	26-*O*-β-d-Glc 3-*O*-α-l-Rha-(1 → 2)-[β-d-Glc-(1 → 4)]-*O*-β-d-Glc	Akhov et al. (1999)
*A. ostrowskianum* Regel		Agigenin [**34**]		Mimaki et al. (1993)
	F-GITONIN [**72**]			Mimaki et al. (1993)
	AGINOSIDE [**93**]			Mimaki et al. (1993)
	[**196**, **197**]	(25*R*,*S*)-5α-furostane-2α,3β,6β,22,26-pentaol	26-*O*-β-d-Glc 3-*O*-β-d-Glc-(1 → 2)-[β-d-Xyl-(1 → 3)]-*O*-β-d-Glc-(1 → 4)-*O*-β-d-Gal	Mimaki et al. (1993)
	[**307**]	Alliosterol [**196**]	16-*O*-β-d-Glc-(1 → 3)-*O*-β-d-Glc	Mimaki et al. (1993)
*A. porrum* L. (*A. ampeloprasum* L. var. *porrum*)		Diosgenin [**4**]		Fattorusso et al. (1998)
		β-Chlorogenin [**12**]		Fattorusso et al. (1998)
		Porrigenin B [**23**]		Carotenuto et al. (1997b)
		Neoporrigenin B [**24**]		Carotenuto et al. (1997b)
		12-Ketoporrigenin [**29**]		Carotenuto et al. (1997b)
		Porrigenin C [**30**]		Fattorusso et al. (2000)
		Agigenin [**34**]		Carotenuto et al. (1997b)
		Neoagigenin [**36**]		Carotenuto et al. (1997b)
		Porrigenin A [**38**]		Carotenuto et al. (1997b)
		Neoporrigenin A [**39**]		Carotenuto et al. (1997b)
		2,3-Seco-porrigenin [**64**]		Carotenuto et al. (1997b)
	F-GITONIN [**72**]			Carotenuto et al. (1999)
	[**74**]	Gitogenin [**9**]	3-*O*-β-d-Glc-(1 → 3)-*O*-β-d-Glc-(1 → 2)-[β-d-Xyl-(1 → 3)]-*O*-β-d-Glc-(1 → 4)-*O*-β-d-Gal	Carotenuto et al. (1999)
	[**78**]	β-Chlorogenin [**12**]	3-*O*-β-d-Glc-(1 → 2)-[β-d-Glc-(1 → 3)]-*O*-β-d-Gal 6-*O*-β-d-Glc	Adão et al. (2011a)
	[**80**]	β-Chlorogenin [**12**]	3-*O*-β-d-Glc-(1 → 2)-[β-d-Xyl-(1 → 3)]-*O*-β-d-Glc-(1 → 4)-*O*-β-d-Gal	Carotenuto et al. (1999)
	[**82**]	β-Chlorogenin [**12**]	3-*O*-β-d-Glc-(1 → 3)-*O*-β-d-Glc-(1 → 2)-[β-d-Xyl-(1 → 3)]-*O*-β-d-Glc-(1 → 4)-*O*-β-d-Gal	Carotenuto et al. (1999)
	AGINOSIDE [**93**]			Harmatha et al. (1987)
	LEUCOSPIROSIDE A [**97**]			Adão et al. (2011b)
	[**162**]	12-Ketoporrigenin [**29**]	3-*O*-β-d-Glc-(1 → 2)-[β-d-Xyl-(1 → 3)]-*O*-β-d-Glc-(1 → 4)-*O*-β-d-Gal	Fattorusso et al. (2000)
	[**175**]	Porrigenin B [**23**]	3-*O*-β-d-Glc-(1 → 3)-*O*-β-d-Glc-(1 → 2)-[β-d-Glc-(1 → 3)]-*O*-β-d-Glc-(1 → 4)-*O*-β-d-Gal	Adão et al. (2012)
	[**177**]	Porrigenin C [**30**]	3-*O*-β-d-Glc-(1 → 2)-[β-d-Xyl-(1 → 3)]-*O*-β-d-Glc-(1 → 4)-*O*-β-d-Gal	Fattorusso et al. (2000)
	[**267**]	Alliosterol [**196**]	1-*O*-α-l-Rha 16-*O*-β-d-Glc	Fattorusso et al. (2000)
	[**308**]	Alliosterol [**196**]	1-*O*-β-d-Glc-(1 → 4)-*O*-α-l-Rha 16-*O*-β-d-Gal	Fattorusso et al. (2000)
*A. rotundum* L.		Tigogenin [**1**]		Maisashvili et al. (2007)
		Diosgenin [**4**]	Sapogenins [**1**, **4**, **7**, **9**, **12**, **19**, **34**] were isolated by hydrolyzing saponins directly in the raw material	Maisashvili et al. (2007)
		Hecogenin [**7**]		Maisashvili et al. (2007)
		Gitogenin [**9**]		Maisashvili et al. (2007)
		β-Chlorogenin [**12**]		Maisashvili et al. (2007)
		Yuccagenin [**19**]		Maisashvili et al. (2007)
		Agigenin [**34**]		Maisashvili et al. (2007)
	[**74**]	Gitogenin [**9**]	3-*O*-β-d-Glc-(1 → 3)-*O*-β-d-Glc-(1 → 2)-[β-d-Xyl-(1 → 3)]-*O*-β-d-Glc-(1 → 4)-*O*-β-d-Gal	Maisashvili et al. (2012)
	DIDEGLUCOERUBOSIDE B [**77**]	β-Chlorogenin [**12**]	3-*O*-β-d-Glc-(1 → 4)-*O*-β-d-Gal	Maisashvili et al. (2008)
	ERUBOSIDE B [**79**]			Maisashvili et al. (2008)
	AGINOSIDE [**93**]			Maisashvili et al. (2008)
	YAYOISAPONIN C [**95**]			Maisashvili et al. (2008)
	TRILLIN (ALLIUMOSIDE A) [**130**]			Maisashvili et al. (2008)
	[**309**]	(25*R*)-5α-furostane-2α,3β,22α,26-tetrol	26-*O*-β-d-Glc 3-*O*-β-d-Glc-(1 → 2)-[β-d-Xyl-(1 → 3)]-*O*-β-d-Glc-(1 → 4)-*O*-β-d-Gal	Maisashvili et al. (2012)
*A. sativum* L.	SATIVOSIDE-R2 [**68**]	Tigogenin [**1**]	3-*O*-β-d-Glc-(1 → 3)-*O*-β-d-Glc-(1 → 2)-[β-d-Xyl-(1 → 3)]-*O*-β-d-Glc-(1 → 4)-*O*-β-d-Gal	Matsuura et al. (1989a)
	F-GITONIN [**72**]			Matsuura et al. (1989a)
	[**76**]	β-Chlorogenin [**12**]	3-*O*-β-d-Gal	Matsuura et al. (1988)
	DIDEGLUCOERUBOSIDE B [**77**]	β-Chlorogenin [**12**]	3-*O*-β-d-Glc-(1 → 4)-*O*-β-d-Gal	Matsuura et al. (1988)
	ERUBOSIDE-B [**79**] (obtained by enzymatic hydrolysis of proto-eruboside B)			Matsuura et al. (1988)
	ISO-ERUBOSIDE-B [**310**]	(25*S*)-5α-spirostane-3β,6β-diol	3-*O*-β-d-Glc-(1 → 2)-[β-d-Glc-(1 → 3)]-*O*-β-d-Glc-(1 → 4)-*O*-β-d-Gal	Peng et al. (1996a)
	SATIVOSIDE-B1 [**311**]	(25*R*)-5α-furostane-3β,6β,22,26-tetrol	26-*O*-β-d-Glc 3-*O*-β-d-Glc-(1 → 3)-*O*-β-d-Glc-(1 → 2)-[β-d-Glc-(1 → 3)]-*O*-β-d-Glc-(1 → 4)-*O*-β-d-Gal	Matsuura et al. (1989a)
	SATIVOSIDE-R1 [**312**]	(25*R*)-5α-furostane-3β,22,26-triol	26-*O*-β-d-Glc 3-*O*-β-d-Glc-(1 → 3)-*O*-β-d-Glc-(1 → 2)-[β-d-Xyl-(1 → 3)]-*O*-β-d-Glc-(1 → 4)-*O*-β-d-Gal	Matsuura et al. (1989a)
	PROTO-ERUBOSIDE-B [**313**]	(25*R*)-5α-furostane-3β,6β,22,26-tetrol	26-*O*-β-d-Glc 3-*O*-β-d-Glc-(1 → 2)-[β-d-Glc-(1 → 3)]-*O*-β-d-Glc-(1 → 4)-*O*-β-d-Gal	Matsuura et al. (1988)
	PROTO-ISO-ERUBOSIDE-B [**314**]	(25*S*)-5α-furostane-3β,6β,22,26-tetrol	26-*O*-β-d-Glc 3-*O*-β-d-Glc-(1 → 2)-[β-d-Glc-(1 → 3)]-*O*-β-d-Glc-(1 → 4)-*O*-β-d-Gal	Peng et al. (1996a), Ma et al. (2011)
	PROTO-DESGALACTOTIGONIN [**315**]			Matsuura et al. (1989a)
	[**316**]	(25*S*)-22-methoxy-5α-furostane-3β,6β,26-triol	26-*O*-β-d-Glc 3-*O*-β-d-Glc-(1 → 2)-[β-d-Glc-(1 → 3)]-*O*-β-d-Glc-(1 → 4)-*O*-β-d-Gal	Ma et al. (2011)
	[**317**]	(25*R*)-22-methoxy-5α-furostane-3β,6β,26-triol	26-*O*-β-d-Glc 3-*O*-β-d-Glc-(1 → 3)-*O*-β-d-Glc-(1 → 2)-[β-d-Glc-(1 → 3)]-*O*-β-d-Glc-(1 → 4)-*O*-β-d-Gal	Ma et al. (2011)
	[**318**]	(25*R*)-22-methoxy-5α,6β-furostane-3β,26-diol	26-*O*-β-d-Glc 3-*O*-β-d-Glc-(1 → 2)-[β-d-Xyl-(1 → 3)]-*O*-β-d-Glc-(1 → 4)-*O*-β-d-Gal	Ma et al. (2011)
*A. sativum* *L*. *var*. Voghiera	[**73**]	Gitogenin [**9**]	3-*O*-β-d-Glc-(1 → 2)-[β-d-Glc-(1 → 3)]-*O*-β-d-Glc-(1 → 4)-*O*-β-d-Gal	Lanzotti et al. (2012a)
	AMPELOSIDE Bs_1_ [**90**]			Lanzotti et al. (2012a)
	VOGHIEROSIDE A1 [**319**]	Furostane-2α,3β,5α,22α,26-pentaol	26-*O*-β-d-Glc 3-*O*-β-d-Glc-(1 → 3)-*O*-β-d-Glc-(1 → 2)-*O*-[β-d-Glc-(1 → 3)]-*O*-β-d-Glc-(1 → 4)-*O*-β-d-Gal	Lanzotti et al. (2012a)
	VOGHIEROSIDE A2 [**320**]	Furostane-2α,3β,5α,22β,26-pentaol	26-*O*-β-d-Glc 3-*O*-β-d-Glc-(1 → 3)-*O*-β-d-Glc-(1 → 2)-[β-d-Glc-(1 → 3)]-*O*-β-d-Glc-(1 → 4)-*O*-β-d-Gal	Lanzotti et al. (2012a)
	VOGHIEROSIDE B1 [**321**]	Furostane-2α,3β,5α,22α,26-pentaol	26-*O*-β-d-Glc 3-*O*-β-d-Glc-(1 → 2)-[β-d-Glc-(1 → 3)]-*O*-β-d-Glc-(1 → 4)-*O*-β-d-Gal	Lanzotti et al. (2012a)
	VOGHIEROSIDE B2 [**322**]	Furostane-2α,3β,5α,22β,26-pentaol	26-*O*-β-d-Glc 3-*O*-β-d-Glc-(1 → 2)-[β-d-Glc-(1 → 3)]-*O*-β-d-Glc-(1 → 4)-*O*-β-d-Gal	Lanzotti et al. (2012a)
	VOGHIEROSIDE C1 [**323**]	Furostane-2α,3β,6β,22α,26-pentaol	26-*O*-β-d-Glc 3-*O*-β-d-Glc-(1 → 2)-[β-d-Glc-(1 → 3)]-*O*-β-d-Glc-(1 → 4)-*O*-β-d-Gal	Lanzotti et al. (2012a)
	VOGHIEROSIDE C2 [**324**]	Furostane-2α,3β,6β,22β,26-pentaol	26-*O*-β-d-Glc 3-*O*-β-d-Glc-(1 → 2)-[β-d-Glc-(1 → 3)]-*O*-β-d-Glc-(1 → 4)-*O*-β-d-Gal	Lanzotti et al. (2012a)
	VOGHIEROSIDE D1 [**325**]	Furostane-2α,3β,22α,26-tetrol	26-*O*-α-l-Rha 3-*O*-β-d-Glc-(1 → 3)-*O*-β-d-Glc-(1 → 2)-[β-d-Glc-(1 → 3)]-*O*-β-d-Glc-(1 → 4)-*O*-β-d-Gal	Lanzotti et al. (2012a)
	VOGHIEROSIDE D2 [**326**]	Furostane-2α,3β,22β,26-tetrol	26-*O*-α-l-Rha 3-*O*-β-d-Glc-(1 → 3)-*O*-β-d-Glc-(1 → 2)-[β-d-Glc-(1 → 3)]-*O*-β-d-Glc-(1 → 4)-*O*-β-d-Gal	Lanzotti et al. (2012a)
	VOGHIEROSIDE E1 [**327**]	Furostane-2α,3β,22α,26-tetrol	26-*O*-α-l-Rha 3-*O*-β-d-Glc-(1 → 2)-[β-d-Glc-(1 → 3)]-*O*-β-d-Glc-(1 → 4)-*O*-β-d-Gal	Lanzotti et al. (2012a)
	VOGHIEROSIDE E2 [**328**]	Furostane-2α,3β,22β,26-tetrol	26-*O*-α-l-Rha 3-*O*-β-d-Glc-(1 → 2)-[β-d-Glc-(1 → 3)]-*O*-β-d-Glc-(1 → 4)-*O*-β-d-Gal	Lanzotti et al. (2012a)
*A. schoenoprasum* *L.*	[**83**]	(25*R*)-5α-spirostane-3β,11α-diol [**14**]	3-*O*-β-d-Glc-(1 → 3)-[β-d-Glc-(1 → 4)]-*O*-β-d-Gal	Timité et al. (2013)
	[**131**]	Diosgenin [**4**]	3-*O*-α-l-Rha-(1 → 2)-*O*-β-d-Glc	Timité et al. (2013)
	DELTONIN [**134**]			Timité et al. (2013)
	[**158**]	Laxogenin [**6**]	3-*O*-α-l-Rha-(1 → 2)-*O*-β-d-Glc	Timité et al. (2013)
	[**160**]	Laxogenin [**6**]	3-*O*-α-l-Rha-(1 → 2)-[β-d-Glc-(1 → 4)]-*O*-β-d-Glc	Timité et al. (2013)
	[**182**]	(20*S*,25*S*)-spirost-5(6)-ene-3β,11α,21-triol [**46**]	3-*O*-α-l-Rha-(1 → 2)-*O*-β-d-Glc	Timité et al. (2013)
	[**183**]	(20*S*,25*S*)-spirost-5(6)-ene-3β,12β,21-triol [**47**]	3-*O*-α-l-Rha-(1 → 2)-*O*-β-d-Glc	Timité et al. (2013)
	DELTOSIDE [**306**]			Timité et al. (2013)
*A. schubertii* Zucc.	[**100**, **126**]	(25*R*,*S*)-5α-spirostane-2α,3β,6β-triol [**34**, **36**]	3-*O*-β-d-Glc-(1 → 2)-[4-*O*-benzoyl-β-d-Xyl-(1 → 3)]-*O*-β-d-Glc-(1 → 4)-*O*-β-d-Gal	Kawashima et al. (1993)
	[**99**, **125**]	(25*R*,*S*)-5α-spirostane-2α,3β,6β-triol [**34**, **36**]	3-*O*-β-d-Glc-(1 → 2)-[3-*O*-benzoyl-β-d-Xyl-(1 → 3)]-*O*-β-d-Glc-(1 → 4)-*O*-β-d-Gal	Kawashima et al. (1993)
	[**98**, **124**]	(25*R*,*S*)-5α-spirostane-2α,3β,6β-triol [**34**, **36**]	3-*O*-β-d-Glc-(1 → 2)-[4-*O*-(*S*)-3-hydroxy-3-methylglutaryl-β-d-Xyl-(1 → 3)]-*O*-β-d-Glc-(1 → 4)-*O*-β-d-Gal	Kawashima et al. (1993)
	[**194**, **195**]	(25*R*,*S*)-5α-furostane-2α,3β,6β,22,26-pentaol	26-*O*-β-d-Glc 3-*O*-β-d-Glc-(1 → 2)-[β-d-Xyl-(1 → 3)]-*O*-β-d-Glc-(1 → 4)-*O*-β-d-Gal	Kawashima et al. (1993)
	SCHUBERTOSIDE D (NIGROSIDE C) [**303**]	Alliosterol [**196**]	1-*O*-α-l-Rha 16-*O*-α-l-Rha-(1 → 3)-*O*-β-d-Gal	Kawashima et al. (1991b)
	SCHUBERTOSIDE A [**329**]	(22*S*)-cholest-4(5)-ene-16β,22-diol-3-one	16-*O*-α-l-Rha-(1 → 3)-*O*-β-d-Gal	Kawashima et al. (1991b)
	SCHUBERTOSIDE B [**330**]	(22*S*)-cholest-5(6)-ene-3β,16β,22-triol	16-*O*-α-l-Rha-(1 → 3)-*O*-β-d-Gal	Kawashima et al. (1991b)
	SCHUBERTOSIDE C [**331**]	(22*S*)-cholest-5(6)-ene-3β,16β,22-triol	3-*O*-β-d-Glc 16-*O*-α-l-Rha-(1 → 3)-*O*-β-d-Gal	Kawashima et al. (1991b)
*A. senescens* *L.*		Diosgenin [**4**]		Inoue et al. (1995)
	[**140**]	Diosgenin [**4**]	3-*O*-α-l-Rha-(1 → 2)-[β-d-Glc-(1 → 3)]-*O*-β-d-Glc	Inoue et al. (1995)
	[**141**]	Diosgenin [**4**]	3-*O*-α-l-Rha-(1 → 4)-*O*-α-l-Rha-(1 → 4)-[α-l-Rha-(1 → 2)]-*O*-β-d-Glc	Inoue et al. (1995)
*A. stipitatum* Regel./*A. suvorovii* Regel.		Diosgenin [**4**]	Sapogenins [4,19] obtained from the acid hydrolysis of the purified combined glycosides	Vollerner et al. (1988a)
		Yuccagenin [**19**]		Vollerner et al. (1988a)
		Anzurogenin B [**26**]		Vollerner et al. (1988a, b)
		Anzurogenin D [**41**]		Kravets (1994)
		Anzurogenin A [**48**]		Vollerner et al. (1988a)
		Alliogenin [**49**]		Vollerner et al. (1988a)
		Anzurogenin C [**58**]		Vollerner et al. (1989)
		Alliosterol [**196**]		Vollerner et al. (1991)
		Alliogenone [**359**] [(25*R*)-5α-spirostane-2α,3β,5α-triol-6-one]		Kravets (1994)
	KARATAVIOSIDE A [**151**]			Kravets (1994)
	KARATAVIOSIDE B [**152**]			Kravets (1994)
	ANZUROSIDE [**190**]	Anzurogenin C [**58**]	24-*O*-β-d-Glc	Vollerner et al. (1989)
	ALLIOSIDE A [**333**]	Alliosterol [**196**]	16-*O*-β-d-Gal	Vollerner et al. (1991)
	ALLIOSIDE B [**334**]	Alliosterol [**196**]	1-*O*-β-d-Glc 16-*O*-β-d-Gal	Vollerner et al. (1991)
*A. triquetrum* L.	ASCALONICOSIDES A1/A2 [**217**, **218**]			Corea et al. (2003)
	TRIQUETROSIDE A1 [**335**]	Furost-5(6)-ene-1β,22α-diol	26-O-α-l-Rha-(1 → 2)-*O*-β-d-Glc 3-O-α-l-Rha-(1 → 2)-O-β-d-Glc	Corea et al. (2003)
	TRIQUETROSIDE A2 [**336**]	Furost-5(6)-ene-1β,22β-diol	26-O-α-l-Rha-(1 → 2)-*O*-β-d-Glc 3-O-α-l-Rha-(1 → 2)-O-β-d-Glc	Corea et al. (2003)
	TRIQUETROSIDE B [**337**]	Furost-5(6),20(22)-diene-1β-ol	26-O-α-l-Rha-(1 → 2)-*O*-β-d-Glc 3-O-α-l-Rha-(1 → 2)-O-β-d-Glc	Corea et al. (2003)
	TRIQUETROSIDE C1 [**338**]	Furost-5(6)-ene-1β,22α-diol	26-O-α-l-Rha-(1 → 2)-*O*-β-d-Glc 3-O-β-d-Glc	Corea et al. (2003)
	TRIQUETROSIDE C2 [**339**]	Furost-5(6)-ene-1β,22β-diol	26-O-α-l-Rha-(1 → 2)-*O*-β-d-Glc 3-O-β-d-Glc	Corea et al. (2003)
*A. tuberosum* Rottl. ex Spreng		Neotigogenin [**2**]	Saponins [**165**, **168**, **171**] obtained after enzymatic hydrolysis of furostanol saponin fraction by β-glucosidase	
	TUBEROSIDE J [**102**]	(25*R*)-5α-spirostane-2α,3β,27-triol [**40**]	3-*O*-α-l-Rha-(1 → 2)-*O*-β-d-Glc	Sang et al. (2001a)
	TUBEROSIDE K [**103**]	(25*R*)-5α-spirostane-2α,3β,27-triol [**40**]	3-*O*-α-l-Rha-(1 → 2)-[α-l-Rha-(1 → 4)]-*O*-β-d-Glc	Sang et al. (2001a)
	TUBEROSIDE L [**104**]	(25*R*)-5α-spirostane-2α,3β,27-triol [**40**]	3-*O*-α-l-Rha-(1 → 2)-[α-l-Rha-(1 → 4)]-*O*-β-d-Glc 27-*O*-β-d-Glc	Sang et al. (2001a)
	NICOTIANOSIDE C [**109**]	Neotigogenin [**2**]	3-*O*-α-l-Rha-(1 → 4)-[α-l-Rha-(1 → 2)]-*O*-β-d-Glc	Sang et al. (2000)
	TUBEROSIDE D [**112**]	Neogitogenin [**10**]	3-*O*-α-l-Rha-(1 → 4)-[α-l-Rha-(1 → 2)]-*O*-β-d-Glc	Sang et al. (1999a, b), Ikeda et al. (2000)
	TUBEROSIDE E [**114**]	Neogitogenin [**10**]	3-*O*-β-d-Glc-(1 → 2)-[α-l-Rha-(1 → 4)]-*O*-β-d-Glc	Sang et al. (1999a)
	TUBEROSIDE [**128**]	(25*S*)-5α-spirostane-2α,3β,27-triol [**42**]	3-*O*-α-l-Rha-(1 → 2)-[α-l-Rha-(1 → 4)]-*O*-β-d-Glc	Zou et al. (2001)
	TUBEROSIDE M [**163**]	(25*S*)-5β-spirostane-1β,3β-diol [**8**]	3-*O*-α-l-Rha-(1 → 4)-*O*-β-d-Glc	Sang et al. (2002)
	TUBEROSIDE N [**164**]	(25*S*)-5β-spirostane-2β,3β-diol [**11**]	3-*O*-β-d-Glc-(1 → 2)-[α-l-Rha-(1 → 4)]-*O*-β-d-Glc	Sang et al. (2003)
	[**165**]	25-Epi-ruizgenin [**13**]	3-*O*-α-l-Rha-(1 → 4)-*O*-β-d-Glc	Ikeda et al. (2000)
	TUBEROSIDE *O* [**166**]	(25*S*)-5β-spirostane-2β,3β,5β-triol [**32**]	3-*O*-β-d-Glc	Sang et al. (2003)
	TUBEROSIDE P [**167**]	(25*S*)-5β-spirostane-2β,3β,5β-triol [**32**]	3-*O*-α-l-Rha-(1 → 4)-*O*-β-d-Glc	Sang et al. (2003)
	[**168**]	(25*S*)-spirostane-3β,5β,6α-triol [**43**]	3-*O*-α-l-Rha-(1 → 4)-*O*-β-d-Glc	Ikeda et al. (2000)
	[**171**]	Lilagenin [**20**]	3-*O*-α-l-Rha-(1 → 4)-[α-l-Rha-(1 → 2)]-*O*-β-d-Glc	Ikeda et al. (2000)
	[**188**]	(24*S*,25*S*)-5β-spirostane-2α,3β,5β,24-tetrol [**55**]	3-*O*-α-l-Rha-(1 → 2)-[α-l-Rha-(1 → 4)]-*O*-β-d-Glc	Hu et al. (2014)
	TUBEROSIDE Q [**189**]	(24*S*,25*S*)-5β-spirostane-2β,3β,5β,24-tetrol [**56**]	3-*O*-α-l-Rha-(1 → 4)-*O*-β-d-Glc	Sang et al. (2003)
	[**340**]	(24*S*,25*S*)-5β-spirostane-2β,3β,24-triol	3-*O*-α-l-Rha-(1 → 2)-[α-l-Rha-(1 → 4)]-*O*-β-d-Glc	Hu et al. (2009)
	TUBEROSIDE A [**341**]	(25*S*)-5α-furost-20(22)-ene-2α,3β,26-triol	26-*O*-β-d-Glc 26-*O*-β-d-Glc	Sang et al. (1999b)
	TUBEROSIDE B [**342**]	(25*S*)-5α-furost-20(22)-ene-2α,3β,26-triol	26-*O*-β-d-Glc 3-*O*-α-l-Rha-(1 → 2)-[α-l-Rha-(1 → 4)]-*O*-β-d-Glc	Sang et al. (1999b)
	TUBEROSIDE C [**343**]	(25*S*)-5α-furost-20(22)-ene-2α,3β,26-triol	26-*O*-β-d-Glc 3-*O*-α-l-Rha-(1 → 2)-[β-d-Glc-(1 → 3)]-*O*-β-d-Glc	Sang et al. (1999b)
	TUBEROSIDE R [**344**]	(25*S*)-5β-furost-20(22)-ene-2β,3β,5,26-tetrol	26-*O*-β-d-Glc 3-*O*-β-d-Glc	Sang et al. (2003)
	TUBEROSIDE S [**345**]	(25*S*)-5β-furost-20(22)-ene-3β,26-diol	26-*O*-β-d-Glc 3-*O*-β-d-Glc-(1 → 2)-[α-l-Rha-(1 → 4)]-*O*-β-d-Glc	Sang et al. (2003)
	TUBEROSIDE T [**346**]	(25*S*)-5α-furost-20(22)-ene-3β,26-diol	26-*O*-β-d-Glc 3-*O*-α-l-Rha-(1 → 2)-[α-l-Rha-(1 → 4)]-*O*-β-d-Glc	Sang et al. (2003)
	[**347**]	(25*S*,20*R*)-5α-furost-22(23)-ene-2α,3β,20,26-tetrol	26-*O*-β-d-Glc 3-*O*-α-l-Rha-(1 → 2)-[α-l-Rha-(1 → 4)]-*O*-β-d-Glc	Sang et al. (2001b)
	[**348**]	(25*S*,20*R*)-20-methoxy-5α-furost-22(23)-ene-2α,3β,20,26-tetrol	26-*O*-β-d-Glc 3-*O*-α-l-Rha-(1 → 2)-[α-l-Rha-(1 → 4)]-*O*-β-d-Glc	Sang et al. (2001b)
	[**349**]	(25*S*,20*S*)-5α-furost-22(23)-ene-2α,3β,20,26-tetrol	26-*O*-β-d-Glc 3-*O*-α-l-Rha-(1 → 2)-[α-l-Rha-(1 → 4)]-*O*-β-d-Glc	Sang et al. (2001b)
	[**350**]	(25*S*,20*S*)-5α-furost-22(23)-ene-3β,20,26-triol	26-*O*-β-d-Glc 3-*O*-α-l-Rha-(1 → 2)-[α-l-Rha-(1 → 4)]-*O*-β-d-Glc	Sang et al. (2001b)
	[**351**]	(25*R*)-5α-furostane-3β,22,26-triol	26-*O*-β-d-Glc 3-*O*-α-l-Rha-(1 → 4)-[α-l-Rha-(1 → 2)]-*O*-β-d-Glc	Ikeda et al. (2004)
	[**352**]	(25*S*)-5β-furostane-3β,5β,6α,22,26-pentaol	26-*O*-β-d-Glc 3-*O*-α-l-Rha-(1 → 4)-*O*-β-d-Glc	Ikeda et al. (2004)
	[**267**]	Alliosterol [**196**]	1-*O*-α-l-Rha 16-*O*-β-d-Glc	Sang et al. (2000)
	TUBEROSIDE U [**353**]	(22*S*,25*S*)-cholest-5(6)-ene-3β,16β,22,26-tetrol	3-*O*-α-l-Rha-(1 → 2)-[α-l-Rha-(1 → 4)]-*O*-β-d-Glc 16-*O*-β-d-Glc	Sang et al. (2003)
*A. turcomanicum* Regel		Yuccagenin [**19**]		Pirtskhalava et al. (1977a)
		Neoagigenone [**25**]		Pirtskhalava et al. (1977a)
		Neoagigenin [**36**]		Pirtskhalava et al. (1977a)
		6-*O*-benzoyl neoagigenin [**37**]		Pirtskhalava et al. (1977a)
		Alliogenin [**49**]		Pirtskhalava et al. (1977a)
		Neoalliogenin [**50**]		Pirtskhalava et al. (1977b)
	TUROSIDE A [**122**]	Neoagigenin [**36**]	3-*O*-β-d-Xyl-(1 → 3)-[β-d-Glc-(1 → 2)]-*O*-β-d-Glc-(1 → 4)-*O*-β-d-Gal	Pirtskhalava et al. (1978)
	TUROSIDE A 6-*O*-BENZOATE [**127**]	6-*O*-benzoyl-neoagigenin [**37**]	3-*O*-β-d-Xyl-(1 → 3)-[β-d-Glc-(1 → 2)]-*O*-β-d-Glc-(1 → 4)-*O*-β-d-Gal	Pirtskhalava et al. (1979a)
	TUROSIDE C [**354**]	(25*S*)-5α-furostane-2α,3β,6β,22,26-pentaol	26-*O*-β-d-Glc 3-*O*-β-d-Xyl-(1 → 3)-[β-d-Glc-(1 → 2)-*O*-β-d-Glc-(1 → 2)]-*O*-β-d-Glc-(1 → 4)-*O*-β-d-Gal	Pirtskhalava et al. (1979b)
*A. ursinum* L.	[**141**]	Diosgenin [**4**]	3-*O*-α-l-Rha-(1 → 4)-*O*-α-l-Rha-(1 → 4)-[α-l-Rha-(1 → 2)]-*O*-β-d-Glc	Sobolewska et al. (2006)
	[**156**]	(25*R*)-spirost-5(6),25(27)-diene-3β-ol [**5**]	3-*O*-α-l-Rha-(1 → 4)-*O*-α-l-Rha-(1 → 4)-[α-l-Rha-(1 → 2)]-*O*-β-d-Glc	Sobolewska et al. (2006)
	DICHOTOMIN [**223**]			Sobolewska (2004)
*A. vavilovii* M. Popov	ASCALONICOSIDES A1/A2 [**217**, **218**]			Zolfaghari et al. (2013)
	VAVILOSIDE A1 [**355**]	(25R)-furost-5(6)-ene-1β,3β,22α,26-tetrol	26-O-α-l-Rha 1-O-α-l-Rha-(1 → 2)-O-β-d-Gal	Zolfaghari et al. (2013)
	VAVILOSIDE A2 [**356**]	(25R)-furost-5(6)-ene-1β,3β,22β,26-tetrol	26-O-α-l-Rha 1-O-α-l-Rha-(1 → 2)-O-β-d-Gal	Zolfaghari et al. (2013)
	VAVILOSIDE B1 [**357**]	(25R)-furost-5(6)-ene-1β,3β,22α,26-tetrol	26-O-α-l-Rha 1-O-α-l-Rha-(1 → 2)-O-β-d-Xyl	Zolfaghari et al. (2013)
	VAVILOSIDE B2 [**358**]	(25R)-furost-5(6)-ene-1β,3β,22β,26-tetrol	26-O-α-l-Rha 1-O-α-l-Rha-(1 → 2)-O-β-d-Xyl	Zolfaghari et al. (2013)
*A. victorialis* var. *platyphyllum* L.	F-GITONIN [**72**]			Lee et al. (2001)
*A. vineale* L.		Diosgenin [**4**]		Chen and Snyder (1989)
		Nuatigenin [**27**]		Chen and Snyder (1989)
		Isonuatigenin [**28**]		Chen and Snyder (1989)
	[**131**]	Diosgenin [**4**]	3-*O*-α-l-Rha-(1 → 2)-*O*-β-d-Glc	Chen and Snyder (1989)
	DELTONIN [**134**]			Chen and Snyder (1989)
	[**136**]	Diosgenin [**4**]	3-*O*-β-d-Glc-(1 → 4)-*O*-α-l-Rha-(1 → 4)-*O*-β-d-Glc	Chen and Snyder (1989)
	[**142**]	Diosgenin [**4**]	3-*O*-β-d-Glc-(1 → 4)-*O*-α-l-Rha-(1 → 4)-[α-l-Rha-(1 → 2)]-*O*-β-d-Glc	Chen and Snyder (1989)
	[**144**]	Diosgenin [**4**]	3-*O*-β-d-Glc-(1 → 3)-[β-d-Glc-(1 → 6)]-*O*-β-d-Glc-(1 → 4)-[α-l-Rha-(1 → 2)]-*O*-β-d-Glc	Chen and Snyder (1989)
	[**145**]	Diosgenin [**4**]	3-*O*-β-d-Glc-(1 → 6)-*O*-β-d-Glc-(1 → 4)-*O*-α-l-Rha-(1 → 4)-[α-l-Rha-(1 → 2)]-*O*-β-d-Glc	Chen and Snyder (1989)
	[**146**]	Diosgenin [**4**]	3-*O*-β-d-Glc-(1 → 4)-[β-d-Glc-(1 → 6)]-*O*-β-d-Glc-(1 → 4)-*O*-α-l-Rha-(1 → 4)-[α-l-Rha-(1 → 2)]-*O*-β-d-Glc	Chen and Snyder (1989)
	[**155**]	Isonuatigenin [**28**]	3-*O*-α-l-Rha-(1 → 2)-*O*-β-d-Glc	Chen and Snyder (1989)
	[**176**]	Nuatigenin [**27**]	3-*O*-α-l-Rha-(1 → 2)-*O*-β-d-Glc	Chen and Snyder (1989)
*A. waldsteinii* Don.		Diosgenin [**4**]		Eristavi et al. (1973)
		β-Chlorogenin [**12**]		Eristavi et al. (1973)
	DIDEGLUCOERUBOSIDE B [**77**]			Gugunishvili et al. (2006)
	TRILLIN (ALLIUMOSIDE A) [**130**]			Gugunishvili et al. (2006)

## Biological and pharmacological properties of *Allium* saponins

Saponins are considered responsible for numerous pharmacological properties of many plants, and they are recognized as active constituents of *Allium* species as well. It should be mentioned, however, that *Allium* plants are not rich sources of these compounds. Results from quantitative studies indicate that saponin content is usually very low, for example *A. nigrum* total saponin content in different parts of the plant was determined as: 19.38 mg/g dw in the roots, 15.65 mg/g dw—bulbs, and 10.48 mg/g dw—leaves (Mostafa et al. 2013). Quantitative densitometric determination of diosgenin—the main sapogenin of *A. ursinum*, revealed some differences in its accumulation with respect to the vegetation period, nevertheless its highest percentage observed in the bulbs collected in March did not exceed 0.0029 % of fresh weight (Sobolewska et al. 2009). A significant exception, in terms of saponin content, is *A. nutans*, where the concentration of these compounds in the underground parts was established to be about 4 % of dry matter (Akhov et al. 1999).

It should be emphasized however that the results from many pharmacological in vitro and in vivo studies revealed several interesting activities of *Allium* saponins, for example antifungal, cytotoxic, antispasmodic, hypocholesterolemic, and other.

### Cytotoxic properties

Cytotoxic activity of saponins was discussed in a number of experimental papers on *Allium* species. *In vitro* studies were performed on several human and animal cell cancer lines, including IGR-1—human melanoma cell line; HL-60—promyelotic leukemia cells; HCT-116, HT-29, and SW480—human colorectal cancer cell lines; DLD-1—human colon adenocarcinoma, HA549—lung cancer cell line, NCI-H460—human large-cell lung carcinoma, SF-268—human glioblastoma; MCF-7—human breast adenocarcinoma, HepG2—human hepatocellular liver carcinoma cell line; WEHI 164—murine fibrosarcoma cell line; J-774—murine monocyte/macrophage cell line; P-388 and L-1210—murine leukemia cell lines (Table 4 of ESM). Amongst tested spirostane saponins dioscin [**135**], isolated from *A. ampleloprasum*, seemed to be most potent, with an IC_50_ = 0.092 μg/mL against P388 cell line (Sata et al. 1998). This compound, which is widely distributed in species of the family Dioscoreaceae and Asparagaceae, revealed significant in vitro activity in tests performed on many other cancer cell lines (Podolak et al. 2010). Some authors claim that apart from the type of the cell line, the structure of the oligosaccharide chain, especially the site of interglycosidic linkages, rather than the sapogenin, are the modulating factors of cytotoxic properties (Rezgui et al. 2014). Some evidence that may substantiate such claims comes from the results obtained for a mixture of diosgenin tetrasaccharide and (25*R*)-spirost-5(6),25(27)-diene-3β-ol tetrasaccharide [**141**, **156**] (*A*. *ursinum*) (Sobolewska et al. 2006). The sugar chain of these compounds differs from that of dioscin [**135**] (3-*O*-α-l-Rha-(1 → 2)-[α-l-Rha-(1 → 4)]-*O*-β-d-Glc) by an additional terminal rhamnose moiety. Both exhibited 100 % effect already at the concentration of 2 μg/mL on melanoma B16 and sarcoma XC. Similarly, deltonin [**134**] (diosgenin 3-*O*-β-d-Glc-(1 → 4)-[α-l-Rha-(1 → 2)]-*O*-β-d-Glc) isolated from *A. schoenoprasum* showed significant activity against HCT 116 and HT-29 cell lines with an IC_50_ = 0.40 and 0.75 μM, respectively (Timité et al. 2013). These results corroborate with those obtained by Mimaki et al. (2001), who suggested that an α-l-Rha-(1 → 2)-*O*-β-d-Glc sugar sequence attached to diosgenin is crucial for activity (Mimaki et al. 2001).

The most potent spirostanol glycosides include also eruboside B [**79**], leucospiroside A [**97**], yayoisaponin C [**95**] and aginoside [**93**] isolated from *A. leucanthum*, which showed in vitro cytotoxic activity, with relatively similar IC_50_ values against A549 WS1, and DLD-1 cells (Mskhiladze et al. 2008b). The two latter compounds, that were isolated from *A*. *ampeloprasum*, showed in vitro cytotoxicity against P388 cells at 2.1 μg/mL (Sata et al. 1998). Tigogenin pentasaccharide [**67**] (*A. macleanii*) and diosgenin 3-*O*-α-l-Rha-(1 → 2)-[β-d-Glc-(1 → 3)]-*O*-β-d-Glc [**140**] (*A. senescens*) were cytotoxic towards HeLa cells at the concentration of 50 μg/mL, whereas already at 5 μg/mL they exhibited 64.7 and 11.5 % inhibition, respectively (Inoue et al. 1995). Several spirostanol glycosides, that were isolated from different *Allium* species, revealed fairly high cytotoxic activity in tests on promyelotic leukemia cells HL-60. Yuccagenin tetrasaccharide (karatavioside A [**151**]) from the bulbs of *A. karataviense* exhibited considerable cytostatic activity with an IC_50_ value of 2.4 μg/mL as compared with etoposide (IC_50_ 0.3 μg/mL) (Mimaki et al. 1999c). Tuberoside M [**163**] from the seeds of *A. tuberosum* inhibited the cells growth with IC_50_ = 6.8 μg/mL, while F-gitonin [**72**] isolated from the fresh bulbs of *A. jesdianum*—with an IC_50_ value of 1.5 μg/mL (Sang et al. 2002; Mimaki et al. 1999a). Other compounds isolated from this latter species were considered to be inactive. The authors concluded that the presence of an additional OH group at C-6 in gitogenin skeleton is detrimental to activity, while cholestane glycosides showed no effect. It is probable that the presence of a carbonyl at C-6 in a laxogenin glycoside [**158**] isolated by Timité et al. (2013) from the whole plant *of A. schoenoprasum* could be responsible for the loss of activity against two cancer cell lines HCT 116 and HT-29, an effect similar to that seen by Mimaki et al. when an additional OH group was introduced at C-6 of gitogenin (Timité et al. 2013; Mimaki et al. 1999c). In accordance with the studies of Mimaki et al. (1999a, b, c) were also the results obtained for cholestane glycosides, nigrosides C [**303**] and D [**304**] isolated from the bulbs of *A. nigrum*, which showed no effect (IC_50_ > 100 μM) on the HT-29 and HCT-116 cancer cell lines in the MTT assay (Jabrane et al. 2011). Opposite results were obtained however with two cholestane glycosides isolated from *A. porrum*—alliosterol 1-*O*-α-l-Rha 16-*O*-β-d-Glc [**267**] and alliosterol 1-*O*-β-d-Glc-(1 → 4)-*O*-α-l-Rha 16-*O*-β-d-Gal [**308**], which exhibited in vitro cytotoxic properties (IC_50_ 4.0–5.8 μg/mL) against two murine cell lines: WEHI 164 and J-774 (Fattorusso et al. 2000).

Results of cytotoxicity assays of several spirostanol sapogenins indicated their weak activity or lack of it. Agigenin [**34**], porrigenin A [**38**] and porrigenin B [**23**] identified in *A.* *porrum* tested in vitro for their growth-inhibitory activity on four different cell lines (IGR-1, WEHI 164, J-774, and P-388) exhibited much weaker activity when compared with 6-MP and were virtually inactive (>100 μg/mL) (Carotenuto et al. 1997a). However, some of the steroidal glycosides isolated from the same plant exhibited quite a good activity towards J-744 and WEHI-164 cells, the most active being gitogenin and porrigenin C derivatives (IC_50_ ranging from 1.9 to 5.8 μg/mL) (Fattorusso et al. 2000).

From among tested furostanoles the majority of compounds showed weak activity or lack of it, for example two glycosides isolated from *A. tuberosum* showed no activity at concentrations below 5 μM against PC-12 and HCT-116 (Ikeda et al. 2004). Among numerous furostanoles obtained from *A. macrostemon* which were tested against NCI-H460, SF-268, MCF-7, and HepG2 cell lines, exclusively 26-*O*-β-d-Glc 5α-furost-25(27)-ene-3β,12β,22,26-tetrol 3-*O*-β-d-Glc-(1 → 2)-[β-d-Glc-(1 → 3)]-*O*-β-d-Glc-(1 → 4)-*O*-β-d-Gal [**292**] was found cytotoxic towards SF-268 cell line, while 26-*O*-β-d-Glc 5β-furost-20(22),25(27)-diene-3β,12β,26-triol 3-*O*-β-d-Glc-(1 → 2)-*O*-β-d-Gal [**293**] showed cytotoxicity towards SF-268 and NCI-H460 cell lines (Chen et al. 2009).

The differences in activity between compounds having the same aglycone but differing in sugar chain was observed by Zolfaghari et al. (2013). The equilibrated mixture of furostanols: vavilosides A1/A2–B1/B2 [**355–358**] and ascalonicosides A1/A2 [**217**, **218**] isolated from *A. vavilovii* were tested against cell lines: J-774 and WEHI-164. The activity of all saponins was dose-dependent and varied in the following order: vavilosides B1/B2 > ascalonicosides A1/A2 > vavilosides A1/A2 (Zolfaghari et al. 2013). The substitution of a galactose residue (vavilosides A1/A2) with a xylose unit (vavilosides B1/B2) caused an increase in cytotoxic activity.

### Antifungal activity

Numerous steroidal saponins isolated from different plant sources have been reported to have antifungal/antiyeast activity, particularly against agricultural pathogens. Antifungal saponins require particular attention as there is a constant need for new agents that would be effective against opportunistic fungal infections and could provide an alternative to chemical fungicides used in the fight against plant pathogens. Unfortunately, only a few studies have been performed so far on *Allium* steroidal glycosides.

Antifungal activity of *Allium* saponins was modulated by both the sapogenin type and the number and structure of the sugar residue. Generally saponins with spirostanol skeleton exhibited higher antifungal activity than furostanols. Yu et al. (2013) observed several biochemical changes which could be involved in the possible mechanism of antimicrobial activity of saponins, such as reduced glucose utilization rate, decrease of catalase activity and protein content in microorganisms.

The results from in vitro assays against different plant and human pathogen strains are provided in Table 5 of ESM.

Studies by Barile et al. (2007), Lanzotti et al. (2012a, b), and Sadeghi et al. (2013) provide evidence for significant differences in the potency of saponins belonging to furostane or spirostane groups. Minutosides A-C [**295**, **119**, **296**] (*A. minutiflorum*) showed concentration-dependent antifungal activity against a number of pathogens: *Alternaria alternata*, *A*. *porri*, *B. cinerea*, *Fusarium oxysporum*, *F*. *solani*, *Pythium ultimum,* *R. solani*, *Trichoderma harzianum* P1, *T. harzianum* T39 (Barile et al. 2007). The most pronounced effect was seen with a spirostanol minutoside B [**119**], as compared to both furostanols (minutosides A [**295**] and C [**296**]). Persicosides A [**120**] and B [**121**]—compounds isolated from *A. ampeloprasum* ssp. *persicum*, showed a statistically significant activity against *P*. *italicum*, *A*. *niger* and *T*. *harzianum*, higher than furostanol and cholestane compounds (Sadeghi et al. 2013). The antifungal activity of isolated compounds against *B*. *cinerea* was not significant. Interestingly, all saponins inhibited the growth of *P*. *italicum*. Antifungal properties of persicosides A [**120**] and B [**121**], ceposides A1/A2 [**209**, **210**], tropeosides A1/A2 [**213**, **214**] and B1/B2 [**215**, **216**] were dose dependent. Ceposides A-C [**209**, **232**, **211**] (isolated from *A. cepa*) showed antifungal activity, dependent on their concentration and the fungal species used: soil-borne pathogens (*Fusarium oxysporum* sp. *lycopersici*, *Rhizoctonia solani* and *Sclerotium cepivorum*), air-borne pathogens (*A. alternata*, *A. niger*, *B*. *cinerea*, *Mucor* sp., and *Phomopsis* sp.), antagonistic fungi (*Trichoderma* *atroviride* and *T. harzianum*), and a pathogen specific to the *Allium* genus—*S*. *cepivorum* (Lanzotti et al. 2012b). Their activity varied in the following order: ceposide B > ceposide A ~ ceposide C. The authors observed a significant synergism of action between those three saponins against *B*. *cinerea* and *T. atroviride*. Ceposide B [**232**] showed significant activity against all fungi with the exception of *F. oxysporum* sp. *lycopersici*, *S. cepivorum* and *R. solani*. Ceposides A [**209**] and C [**211**] were active against all fungi with the exception of *A*. *niger*, *S*. *cepivorum* and *F. oxysporum* sp. *lycopersici*. Agigenin 3-*O*-trisaccharide [**90**] and gitogenin 3-*O*-tetrasaccharide [**73**], isolated from the bulbs of *A. sativum* var. Voghiera, were more active against *B. cinerea* and *T. harzianum* than furostanol voghierosides isolated from that plant (Lanzotti et al. 2012a). All the compounds were effective towards *T*. *harzianum* in a dose dependent manner, but only spirostanol saponins and voghieroside C [**323**, **324**]—against *B. cinerea*.

Mskhiladze et al. (2008a) in their studies on anti-yeast effects of saponins from *A. leucanthum* observed that β-chlorogenin as aglycone and the branched oligosaccharide chain substituted by xylose rather than glucose are beneficial for the activity. Yayoisaponin C [**95**], eruboside B [**79**], aginoside [**93**], agigenin 3-*O*-β-d-Glc-(1 → 2)-*O*-β-d-Glc-(1 → 4)-*O*-β-d-Gal [**91**] and β-chlorogenin 3-*O*-β-d-Glc-(1 → 2)-[β-d-Xyl-(1 → 3)]-*O*-β-d-Glc-(1 → 4)-*O*-β-d-Gal [**80**] exhibited antifungal activity on several *Candida* strains, including *C*. *albicans*, *C*. *tropicalis*, *C*. *parapsilosis*, *C*. *glabrata*, *C*. *kefyr*, *C*. *krusei*, *C*. *lusitaniae*, and also on *Cryptococcus neoformans*, however β-chlorogenin glycoside was the most active compound with MFC from ≤6.25 to 25 μg/mL (as compared to amphotericin B 0.78–12.5 μg/mL). In another study the same compound isolated from *A. porrum* showed antifungal activity towards *Fusarium culmorum* (ED_50_ = 30 μg/mL) (Carotenuto et al. 1999). Eruboside B [**79**] (*A.* *sativum*), β-chlorogenin glycoside as well, inhibited in vitro the growth of *C. albicans* (MIC 25 μg/mL) (Matsuura et al. 1988).

Agigenin glycosides: aginoside [**93**] together with yayoisaponins A [**96**] and C [**95**] isolated from *A.* *ampeloprasum* showed antifungal activity against *Mortierella ramanniana* at 10 μg/disc (Sata et al. 1998). None of the saponins was active against *Penicillium chrysogenum* at concentrations up to 100 μg/disc. Ampeloside Bs_1_ [**90**], agigenin 3-*O*-β-d-Glc-(1 → 4)-*O*-β-d-Gal [**87**], and furostane-type ampeloside Bf_1_ [**202**] isolated from the same species did not inhibit the growth of *Aspergillus niger*; spirostanols showed weak activity against *Candida albicans* (Morita et al. 1988). Aginoside [**93**] at 400 ppm completely inhibited the growth of *C. gloeosporioides*, *Fusarium verticillioides*, and *Botrytis squamosa* and partially suppressed *F. oxysporum* f. sp. *cepae* and *F. oxysporum* f. sp. *radicis*-*lycopersici* (Mostafa et al. 2013). The influence of the structure of the sugar chain on the observed anti-fungal activity of compounds bearing the same aglycone was revealed in studies by Teshima et al. (2013).

Alliospirosides A [**169**] and B [**170**] (both (25*S*)-ruscogenin glycosides), which are present mainly in the basal plates and roots of *A. cepa* Aggregatum group, to a different extent inhibited in vitro a wide range of plant pathogenic fungi: *Alternaria* ssp., *Botrytis* ssp., *Colletotrichum* spp., *Curvularia lunata*, *Epicoccum nigrum*, *Fusarium* ssp., *Magnaporthe oryzae,* *S. cepivorum*, and *Thanatephorus cucumeris* (Teshima et al. 2013). Alliospiroside A [**169**] strongly inhibited (>80 % growth inhib.) the growth of *Colletotrichum* spp. isolates. It was also more effective against *M. oryzae* and *S. cepivorum* compared to alliospiroside B [**170**], however, its antifungal activity against *B. cinerea*, *F. oxysporum* and *F. solani* was relatively low.

### Enzyme inhibitory properties

Saponin fraction isolated from the methanol extract of *A.* *chinense* inhibited cAMP PDE (43.5 %) and Na^+^/K^+^ATP-ase (59.3 %) at the concentration of 100 μg/mL (Kuroda et al. 1995). Both enzymes were also inhibited by (25*R*,*S*)-5α-spirostane-3β-ol tetrasaccharide [**65**, **110**] (IC_50_ 7.0 × 10^−5^ and 4.0 × 10^−5^ M respectively). Laxogenin glycosides exhibited significant activity only on cAMP phosphodiesterase, one of which, with an acetyl group in the saccharide moiety, was almost as potent as papaverine used as a positive control (IC_50_ 3.3 × 10^−5^ and 3.0 × 10^−5^ M respectively).

Also, saponins isolated from *A. giganteum* bulbs inhibited cAMP phosphodiesterase (Mimaki et al. 1994) and in concordance with previously cited results, an acetyl derivative—3-*O*-acetyl-(24*S*,25*S*)-5α-spirostane-2α,3β,5α,6β,24-pentaol 2-*O*-β-d-Glc [**193**] exhibited inhibitory activity almost equal to that of papaverine (IC_50_ 4.1 × 10^−5^ and 3.0 × 10^−5^ M respectively). In the same study, furostanol saponins were revealed to be much more potent than the corresponding spirostanol glycosides. The results were in contrast to the previous studies of these authors which showed that furostanol glycosides were less active, exhibiting only weak inhibitory activity or none. The authors concluded that the anti-enzyme activity could be dependent on the number of hydroxyls in the A and B rings as in the present study the tested furostanol saponins contained several OH groups.

Saponins isolated from the fruits of *A. karataviense* and *A. cepa* as well as the products of chemical modifications of karatavioside A, were studied on a highly purified porcine kidney Na^+^/K^+^ATP-ase, in the concentration range from 1 × 10^−4^ to 1 × 10^−7^ M (Mirsalikhova et al. 1993). All the compounds affected the enzyme activity being capable of its inhibition, and/or activation. As was showed, the presence of a hydroxyl group in the F-ring at C-24 led to a decrease in the percentage inhibition of Na^+^/K^+^ATP-ase. At the concentration of 1 × 10^−4^ M the inhibitory effect of karatavioside A [**151**] was 19.8 %, karatavioside B [**152**]**—**32.4 %, karatavioside C [**268**]**—**4.9 %, karatavioside E [**180**]**—**1.7 %, karatavioside F [**181**]**—**7.5 %, alliospiroside A [**169**]**—**99.7 %, alliospiroside B [**170**]**—**76.3 %, alliospiroside D [**179**]**—**67.1 %; while alliospiroside C [**178**] activated the enzyme by 13.4 %. A keto group at C-6 of sapogenin slightly increased the inhibition level of Na^+^/K^+^ATP-ase.

Moreover, it was revealed that alliospirosides A [**169**] and B [**170**] were both uncompetitive enzyme inhibitors, while alliospiroside D [**179**]**—**competitive. Interestingly, alliospiroside C [**178**], although bearing the same aglycone as alliospiroside D**—**cepagenin [**44**], did not inhibit Na^+^/K^+^ATP-ase at all.

Drugs acting via inhibition of the activity of this transport enzyme may be of potential use in the treatment of many diseases of the cardiovascular system, the kidneys, the immune system, which are connected with disturbances in the active transport of ions.

### Cardioprotective activity

Three saponins from *A. chinense* and their aglycones were tested for the protective effects against oxidative stress-induced cardiac damage (Ren et al. 2010). Their activities were evaluated on H_2_O_2_-injured cardiac H9C2 cells. The cytotoxicity was measured using MTT assay while the oxidative damage by determination of MDA and NO contents. All tested compounds protected cultured H9C2 cells from death in the concentration range of 5–20 μΜ. It was shown that glycosides exhibited less protective efficacy than sapogenins. Among these, laxogenin [**6**] and tigogenin [**1**] displayed stronger effects than furostane-type aglycones. The authors concluded that the presence of F ring in spirostanols may enhance their protective activity whereas oxidation in the B ring might be detrimental as laxogenin was less active than tigogenin.

Nine furostane saponins isolated by Lai et al. from *A. fistulosum* were tested for antihypoxic activity against hypoxia/reoxygenation (H/R)-induced human umbilical vein endothelial cell (HUVEC) injury (Lai et al. 2010). Cell viability was determined by MTT assay. It was observed that the saponin treatment significantly improved the survival of H/R-treated HUVEC (*P* < 0.05) in a dose-dependent manner. Fistulosaponin A [**250**] was the most effective compound with a cell viability of 59.5 ± 3.0, 76.3 ± 3.3, 80.1 ± 3.6, 82.7 ± 4.1, 86.3 ± 4.6, and 78.2 ± 2.8 % for the six dose groups (0.5, 1, 5, 10, 50, and 100 μM), respectively.

In animal studies, alloside B [**334**], isolated from fruits of *A. suvorovii* and *A. stipitatum*, exhibited a statistically reliable hypotriglyceridemic activity in experimental hyperlipidemia caused by 1-day starvation, Triton WR-1339 and vitamin D_2_–cholesterol, when compared with lipanthyl (Aizikov et al. 1995).

The hypocholesterolemic activity of saponins was reported in many animal studies.

The cholesterol-lowering effect of garlic is probably partially due to the steroid saponin presence. In a rat model of experimental hyperlipidemia induced by feeding a 0.5 % cholesterol-enriched diet saponin-rich fraction from raw garlic administrated at 10 mg/kg/day led to a decrease of plasma total and LDL cholesterol concentration level without affecting HDL cholesterol levels after 16 weeks (Matsuura 2001). It was claimed that the reduction of concentration of plasma cholesterol concentration is the result of inhibition of cholesterol absorption by saponins in the intestine or a direct effect on cholesterol metabolism.

### Antispasmodic effect

Furostanol saponins hirtifoliosides C1/C2 [**264**, **265**] and a spirostanol glycoside agapanthagenin 3-*O*-Glc [**85**] isolated from *A*. *hirtifolium*, along with four saponins elburzensosides A1/A2 [**238**, **239**] and C1/C2 [**242**, **243**] and the sapogenin agapanthagenin [**31**], from *A*. *elburzense*, were subjected to biological assays on the guinea-pig isolated ileum in order to evaluate their possible antispasmodic activity (Barile et al. 2005). Apart from the agapanthagenin glycoside, all the tested compounds were able to reduce induced contractions, as measured by the reduction of histamine release, in a concentration-dependent manner. Elburzensosides C1/C2 [**242**, **243**] and agapanthagenin [**31**] showed the highest activity with a maximum effect at 10^−5^M (approx. 50 % inhibition).

The authors concluded that the positive effect is associated with the presence of a hydroxyl group at position C-5 and of a glucose unit at position C-26. On the other hand, hydroxylation at C-6 and glucose attachment at C-3 seem to be structural features responsible for the loss of activity. Furostane-type saponins that were isolated from *A. cepa* var*. tropea*, namely tropeosides A1/A2 [**213**, **214**] and B1/B2 [**215**, **216**] were able to dose-dependently relieve acetylocholine- and histamine-induced contractions (50 % inhibition of contractions was seen at the concentration of 10^−5^ M) (Corea et al. 2005). Interestingly, other furostanols identified in this plant, such as ascalonicosides A1/A2 [**217**, **218**], were inactive.

### Other activities

Macrostemonoside A [**65**] inhibited ADP-induced rabbit ertythrocyte aggregation with IC_50_ = 0.065 mM (Peng et al. 1992). An in vitro inhibitory activity of ADP-induced platelet aggregation was also reported for macrostemonosides E [**272**], F [**273**] and G [**274**] (IC_50_ = 0.417; 0.020; 0.871 mM, respectively) (Peng et al. 1993, 1995). 26-*O*-β-d-Glc (25*R*)**-**5α-furostane**-**3β,12β,22,26**-**tetrol 3-*O*-β-d-Glc-(1 → 2)-[β-d-Glc-(1 → 3)]-*O*-β-d-Glc-(1 → 4)-*O*-β-d-Gal [**289**] and 26-*O*-β-d-Glc (25*R*)-5α-furostane-3β,12α,22,26-tetrol 3-*O*-β-d-Glc-(1 → 2)-[β-d-Glc-(1 → 3)]-*O*-β-d-Glc-(1 → 4)-*O*-β-d-Gal [**290**] exhibited significant inhibitory activity on CD40L expression on the membrane of ADP stimulated platelets (Chen et al. 2010).

β-chlorogenin 3-*O*-β-d-Glc-(1 → 2)-[β-d-Glc-(1 → 3)]-*O*-β-d-Gal 6-*O*-β-d-Glc [**78**], isolated from the bulbs of *A. ampeloprasum* var. *porrum*, demonstrated in vivo antiinflammatory and gastroprotective effects in a carrageenan-induced oedema assay and by measuring acute gastric lesions induced by acidified ethanol (Adão et al. 2011a). Saponin administrated orally (100 mg/kg) inhibited oedema formation similar to dexamethasone (25 mg/kg). Cytoprotective activity of β-chlorogenin glycoside resulted in a significant reduction in gastric hyperemia and also in the severity and number of lesions.

Macrostemonoside A [**65**] increased the synthesis and release of visfatin in 3T3-L1 adipocytes and elevated mRNA levels in this cytokine in a dose- and time-dependent mode (Zhou et al. 2007). In a study on C57BL/6 mice fed on a high-fat diet, this saponin when administered at the dose of 4 mg/kg/day for 30 days moderately inhibited glucose level, glycogen hepatic content, total plasma cholesterol level and abdominal adipose tissue (Xie et al. 2008).

In the molluscicidal bioassay with *Biomphalaria pfeifferei* diosgenin 3-*O*-β-d-Glc-(1 → 4)-[β-d-Glc-(1 → 6)]-*O*-β-d-Glc-(1 → 4)-*O*-α-l-Rha-(1 → 4)-[α-l-Rha-(1 → 2)]-*O*-β-d-Glc [**146**], isolated from *A. vineale*, exhibited 100 % effect at 25 ppm in <24 h (Chen and Snyder 1989). The authors observed that the molluscicidal activity of isolated compounds increased with an increasing number of monosaccharides in a sugar moiety.

Aginoside [**93**] was found to be toxic to leek-moth larvae *Acrolepiopsis assectella* (Harmatha et al. 1987). The compound caused mortality and ecdysial failures 56 ± 10 and 19 % respectively in larvae of *A. assectella* reared on semisynthetic diet at a concentration of 0.9 mg/g of diet.

## Conclusions

In this paper steroidal saponins reported in various *Allium* species from early 1970 to March 2014 are reviewed, including their skeletal structures and sugar chains.

Until now, as many as 290 saponins have been identified, including a certain number of methoxyl derivatives originating from furostanol compounds, that should be considered as artifacts resulting from the use of methanol in the extraction/isolation procedures.

*Allium* genus is characterized by a great diversity of structures. Apart from spirostane- and furostane-type compounds, a rare group of open-chain saponins has been identified in several species. *Allium* genus is also a source of unique steroidal sapogenins, such as 25(*S*)-5β-spirostane-1β,3β-diol [**8**] and 2,3-seco-porrigenin [**64**]. Despite a relatively low content of steroidal glycosides in *Allium* species, they are considered to contribute, in addition to sulfur compounds, to the overall biological activity of these plants. Undoubtedly, stability of saponins is their advantage as compared to fairly unstable sulfur compounds, thus, they in fact may be predominant active constituents of *Allium* products. Bearing this aspect in mind it seems highly feasible to develop antifungal *Allium* preparations against animal and plant pathogens. Also, reports on high in vitro cytotoxic activity of steroidal saponins from *Allium* species makes them potential candidates for further development as anti-cancer agents.

## Electronic supplementary material

Below is the link to the electronic supplementary material.

Supplementary material 1 (DOC 410 kb)

Supplementary material 2 (DOC 178 kb)
