# Supplementary material for: Steroidal saponins from the genus Allium
Source: Phytochem Rev. 2014 Oct 8;15(1):1–35. doi: 10.1007/s11101-014-9381-1 (PMC4735241; doi:10.1007/s11101-014-9381-1)
Supplement: Supplementary file 1 — Supplementary material 1 (DOC 410 kb) [file 11101_2014_9381_MOESM1_ESM.doc]

Table 3. Spirostanol compounds from the genus *Allium*.

| **Aglycone** | | | | | | | | | | **Sugar residue** | **Glycoside**  **[No.]** | **References** |
| --- | --- | --- | --- | --- | --- | --- | --- | --- | --- | --- | --- | --- |
|  | **[No.]** | R_1_ | | R_2_ | R_3_ | | R_4_ | | R_5_ |  |  |  |
| **[1]** | H | | H | H | | H | | CH_3_ | 3-O-β-D-Glc-(1→2)-[β-D-Glc-(1→3)]-O-β-D-Glc-(1→4)-O-β-D-Gal | **[65]** | Peng et al. 1992 |
| 3-O-β-D-Glc-(1→2)-[β-D-Glc-(1→3)-(6-O-acetyl-β-D-Glc)]-(1→4)-O-β-D-Gal | **[66]** | Peng et al. 1992; Jiang et al. 1998 |
| 3-O-α-L-Rha-(1→2)-O-β-D-Xyl-(1→2)-[β-D-Xyl-(1→3)]-O-β-D-Glc-(1→4)-O-β-D-Gal | **[67]** | Inoue et al. 1995 |
| 3-O-β-D-Glc-(1→3)-O-β-D-Glc-(1→2)-[β-D-Xyl-(1→3)]-O-β-D-Glc-(1→4)-O-β-D-Gal | **[68]** | Matsuura et al. 1989a |
| **[9]** | α-OH | | H | H | | H | | CH_3_ | 3-O-β-D-Glc-(1→4)-O-β-D-Glc | **[69]** | Barile et al. 2004; Barile et al. 2005 |
| 3-O-β-D-Glc-(1→4)-O-β-D-Gal | **[70]** | Mimaki et al. 1999c |
| 3-O-β-D-Glc-(1→2)-O-β-D-Glc-(1→4)-O-β-D-Gal | **[71]** | Jiang et al. 1998 |
| 3-O-β-D-Glc-(1→2)-[β-D-Xyl-(1→3)]-O-β-D-Glc-(1→4)-O-β-D-Gal | **[72]** | Matsuura et al. 1989a; Mimaki et al. 1993; Mimaki et al. 1999c; Carotenuto et al. 1999; Lee et al. 2001; Tolkacheva et al. 2012 |
| 3-O-β-D-Glc-(1→2)-[β-D-Glc-(1→3)]-O-β-D-Glc-(1→4)-O-β-D-Gal | **[73]** | Kuroda et al. 1995; Lanzotti et al. 2012a |
| 3-O-β-D-Glc-(1→3)-O-β-D-Glc-(1→2)-[β-D-Xyl-(1→3)]-O-β-D-Glc-(1→4)-O-β-D-Gal | **[74]** | Carotenuto et al. 1999; Maisashvili et al. 2012 |
| 3-O-β-D-Glc-(1→2)-[4-O-(*S*)-3-hydroxy-3-methylglutaryl-β-D-Xyl-(1→3)]-O-β-D-Glc-(1→4)-O-β-D-Gal | **[75]** | Mimaki et al. 1999c; Tolkacheva et al. 2012 |
| **[12]** | H | | H | β-OH | | H | | CH_3_ | 3-O-β-D-Gal | **[76]** | Matsuura et al. 1988 |
| 3-O-β-D-Glc-(1→4)-O-β-D-Gal | **[77]** | Matsuura et al. 1988; Gugunishvili et al. 2006; Maisashvili et al. 2008 |
| 3-O-β-D-Glc-(1→2)-[β-D-Glc-(1→3)]-O-β-D-Gal 6-O-β-D-Glc | **[78]** | Adão et al. 2011a |
| 3-O-β-D-Glc-(1→3)-[β-D-Glc-(1→2)]-O-β-D-Glc-(1→4)-O-β-D-Gal | **[79]** | Chincharadze et al. 1979; Matsuura et al. 1988; Kravets et al. 1990; Maisashvili et al. 2008 |
| 3-O-β-D-Glc-(1→2)-[β-D-Xyl-(1→3)]-O-β-D-Glc-(1→4)-O-β-D-Gal | **[80]** | Mskhiladze et al. 2008; Carotenuto 1999 |
| 3-O-β-D-Glc-(1→3)-O-β-D-Glc-(1→2)-[β-D-Glc-(1→3)]-O-β-D-Glc-(1→4)-O-β-D-Gal | **[81]** | Mskhiladze et al. 2008 |
| 3-O-β-D-Glc-(1→3)-O-β-D-Glc-(1→2)-[β-D-Xyl-(1→3)]-O-β-D-Glc-(1→4)-O-β-D-Gal | **[82]** | Carotenuto 1999 |
| **[14]** | H | | H | H | | OH | | CH_3_ | 3-O-β-D-Glc-(1→3)-[β-D-Glc-(1→4)]-O-β-D-Gal | **[83]** | Timité et al. 2013 |
| **[31]** | α-OH | | OH | H | | H | | CH_3_ | 2-O-β-D-Glc | **[84]** | Mimaki et al. 1999c |
| 3-O-β-D-Glc | **[85]** | Barile et al. 2004; Barile et al. 2005 |
| **[33]** | α-OH | | H | α-OH | | H | | CH_3_ | 3-O-β-D-Glc-(1→2)-[β-D-Xyl-(1→3)]-O-β-D-Glc-(1→4)-O-β-D-Gal | **[86]** | Mimaki et al. 1999c |
| **[34]** | α-OH | | H | β-OH | | H | | CH_3_ | 3-O-β-D-Glc-(1→4)-O-β-D-Gal | **[87]** | Morita et al. 1988; Zolfaghari et al. 2006 |
| 2-O-β-D-Glc 3-O-β-D-Gal | **[88]** | Jabrane et al. 2011 |
| 3-O-α-L-Rha-(1→2)-O-β-D-Glc | **[89]** | Jabrane et al. 2011 |
| 3-O-β-D-Glc-(1→3)-O-β-D-Glc-(1→4)-O-β-D-Gal | **[90]** | Morita et al. 1988;  Lanzotti et al. 2012a |
| 3-O-β-D-Glc-(1→2)-O-β-D-Glc-(1→4)-O-β-D-Gal | **[91]** | Mskhiladze et al. 2008b |
| 3-O-β-D-Xyl-(1→3)-O-β-D-Glc-(1→4)-O*-*β-D-Gal | **[92]** | Barile et al. 2005 |
| 3-O-β-D-Glc-(1→2)-[β-D-Xyl-(1→3)]-O-β-D-Glc-(1→4)-O-β-D-Gal | **[93]** | Kelginbaev et al. 1976; Harmatha et al. 1987; Kawashima et al. 1991a; Mimaki et al. 1993; Inoue et al. 1995 Sata et al. 1998; Maisashvili et al. 2008; Mskhiladze et al. 2008b; Jabrane et al. 2011; Mostafa et al. 2013 |
| 3-O-β-D-Glc-(1→2)-[3-O-acetyl-β-D-Xyl-(1→3)]-O-β-D-Glc-(1→4)-O-β-D-Gal | **[94]** | Mimaki et al. 1993 |
| 3-O-β-D-Glc-(1→2)-[β-D-Glc-(1→3)]-O-β-D-Glc-(1→4)-O-β-D-Gal | **[95]** | Sata et al. 1998; Mskhiladze et al. 2008b; Maisashvili et al. 2008 |
| 3-O-β-D-Glc-(1→3)-O-β-D-Glc-(1→2)-[β-D-Xyl-(1→2)]-O-β-D-Glc-(1→4)-O-β-D-Gal | **[96]** | Sata et al. 1998 |
| 3-O-β-D-Glc-(1→3)-O-β-D-Glc-(1→2)-[β-D-Glc-(1→3)]-O-β-D-Glc-(1→4)-O-β-D-Gal | **[97]** | Adão et al. 2011b; Mskhiladze et al. 2008 |
| 3-O-β-D-Glc-(1→2)-[4-O-(*S*)-3-hydroxy-3-methylglutaryl-β-D-Xyl-(1→3)]-O-β-D-Glc-(1→4)-O-β-D-Gal | **[98]** | Kawashima et al. 1993; Mimaki et al. 1994; Inoue et al. 1995; Jabrane et al. 2011 |
| 3-O-β-D-Glc-(1→2)-[3-O-benzoyl-β-D-Xyl-(1→3)]-O-β-D-Glc-(1→4)-O-β-D-Gal | **[99]** | Kawashima et al. 1993 |
| 3-O-β-D-Glc-(1→2)-[4-O-benzoyl-β-D-Xyl-(1→3)]-O-β-D-Glc-(1→4)-O-β-D-Gal | **[100]** | Kawashima et al. 1993; Inoue et al. 1995 |
| **[35]** | (*S*)-HMG | | H | β-OH | | H | | CH_3_ | 3-O-β-D-Glc-(1→2)-[β-D-Xyl-(1→3)]-O-β-D-Glc-(1→4)-O-β-D-Gal | **[101]** | Mimaki et al. 1993 |
| **[38]** | β-OH | | H | β-OH | | H | | CH_3_ | ------------------------ |  | Carotenuto 1997b |
| **[40]** | α-OH | | H | H | | H | | CH_2_OH | 3-O-α-L-Rha-(1→2)-O-β-D-Glc | **[102]** | Sang et al. 2001a |
| 3-O-α-L-Rha-(1→2)-[α-L-Rha-(1→4)]-O-β-D-Glc | **[103]** | Sang et al. 2001a |
| 3-O-α-L-Rha-(1→2)-[α-L-Rha-(1→4)]-O-β-D-Glc  27-O-β-D-Glc | **[104]** | Sang et al. 2001a |
| **[41]** | H | | OH | β-OH | | H | | CH_3_ | ------------------------- |  | Kravets 1994 |
| **[49]** | α-OH | | OH | β-OH | | H | | CH_3_ | 2-O-β-D-Glc | **[105]** | Sashida et al. 1991; Kawashima et al. 1991a; Mimaki et al. 1993; Inoue et al. 1995; Mimaki et al. 1999c |
| 3-O-β-D-Glc | **[106]** | Gorovits et al. 1971; Gorovits et al. 1973; Barile et al. 2004; Barile et al. 2005 |
| 3-O-β-D-Glc-(1→2)-O-[β-D-Xyl-(1→3)]-O-β-D-Glc-(1→4)-O-β-D-Gal | **[107]** | Mimaki et al. 1999c |
| **[57]** | α-OH | | H | β-OH | | H | | CH_2_OH | 3-O-β-D-Glc-(1→4)-O-β-D-Gal | **[108]** | Zolfaghari et al. 2006 |
|  |  | R_1_ | | R_2_ | R_3_ | | R_4_ | |  | |  |  |
| **[2]** | H | | H | H | | CH_3_ | | 3-O-α-L-Rha-(1→4)-[α-L-Rha-(1→2)]-O-β-D-Glc | | **[109]** | Sang et al. 2000 |
| 3-O-β-D-Glc-(1→2)-[β-D-Glc-(1→3)]-O-β-D-Glc-(1→4)-O-β-D-Gal | | **[110]** | Kuroda et al. 1995; Jiang et al. 1998 |
| 3-O-β-D-Glc-(1→2)-[β-D-Glc-(1→3)-(6-O-acetyl-β-D-Glc)]-(1→4)-O-β-D-Gal | | **[111]** | Jiang et al. 1998; Jiang et al. 1999 |
| **[10]** | α-OH | | H | H | | CH_3_ | | 3-O-α-L-Rha-(1→4)-[α-L-Rha-(1→2)]-O-β-D-Glc | | **[112]** | Sang et al. 1999b; Sang et al. 1999a; Ikeda et al. 2000 |
| 3-O-β-D-Glc-(1→2)-O-β-D-Glc-(1→4)-O-β-D-Gal | | **[113]** | Jiang et al. 1998 |
| 3-O-β-D-Glc-(1→2)-[α-L-Rha-(1→4)]-O-β-D-Glc | | **[114]** | Sang et al. 1999a |
| 3-O-β-D-Glc-(1→2)-[β-D-Glc-(1→3)]-O-β-D-Glc-(1→4)-O-β-D-Gal | | **[115]** | Kuroda et al. 1995 |
| **[16]** | H | | H | OH | | CH_3_ | | 3-O-α-L-Ara-(1→6)-O-β-D-Glc 24-O-β-D-Glc | | **[116]** | Jiang et al. 1998 |
| **[36]** | α-OH | | OH | H | | CH_3_ | | 3-O-α-L-Rha-(1→2)-O-β-D-Glc | | **[117]** | Jabrane et al. 2011 |
| 2-O-β-D-Glc 3-O-β-D-Gal | | **[118]** | Jabrane et al. 2011 |
| 3-O-β-D-Xyl-(1→3)-O-β-D-Glc-(1→4)-O-β-D-Gal | | **[119]** | Barile et al. 2007 |
| 3-O-β-D-Glc-(1→3)-[β-D-Xyl-(1→2)]-O-β-D-Glc-(1→4)-O-β-D-Gal | | **[120]** | Sadeghi et al. 2013 |
| 3-O-β-D-Xyl-(1→3)-[α-L-Rha-(1→2)]-O-β-D-Glc-(1→4)-O-β-D-Gal | | **[121]** | Sadeghi et al. 2013 |
| 3-O-β-D-Xyl-(1→3)-[β-D-Glc-(1→2)]-O-β-D-Glc-(1→4)-O-β-D-Gal | | **[122]** | Pirtskhalava et al. 1978; Jabrane et al. 2011 |
| 3-O-β-D-Glc-(1→2)-[3-O-acetyl-β-D-Xyl-(1→3)]-O-β-D-Glc-(1→4)-O-β-D-Gal | | **[123]** | Mimaki et al. 1993 |
| 3-O-β-D-Glc-(1→2)-[4-O-(*S*)-3-hydroxy-3-methylglutaryl-β-D-Xyl-(1→3)]-O-β-D-Glc-(1→4)-O-β-D-Gal | | **[124]** | Kawashima et al. 1993; Jabrane et al. 2011 |
| 3-O-β-D-Glc-(1→2)-[3-O-benzoyl-β-D-Xyl-(1→3)]-O-β-D-Glc-(1→4)-O-β-D-Gal | | **[125]** | Kawashima et al. 1993 |
| 3-O-β-D-Glc-(1→2)-[4-O-benzoyl-β-D-Xyl-(1→3)]-O-β-D-Glc-(1→4)-O-β-D-Gal | | **[126]** | Kawashima et al. 1993 |
| **[37]** | α-OH | | benzoyl | H | | CH_3_ | | 3-O-β-D-Xyl-(1→3)-[β-D-Glc-(1→2)]-O-β-D-Glc-(1→4)-O-β-D-Gal | | **[127]** | Pirtskhalava et al. 1979a |
| **[39]** | β-OH | | OH | H | | CH_3_ | | ------------------------------- | |  | Carotenuto 1997b |
| **[42]** | α-OH | | H | H | | CH_2_OH | | 3-O-α-L-Rha-(1→2)-[α-L-Rha-(1→4)]-O-β-D-Glc | | **[128]** | Zou et al. 2001 |
| **[50]** | α-OH | | OH | H | | CH_3_ | | ------------------------------- | |  | Pirtskhalava et al. 1977b |
|  |  | R_1_ | |  | | | | | | |  |  |
| **[3]** | H | | ------------------------------------- | | | | | | |  | Okanishi et al.1975 |
| **[15]** | OH | | 3-O-β-D-Glc-(1→2)-O-β-D-Gal | | | | | | | **[129]** | Cheng et al. 2013 |
|  |  | R_1_ | | R_2_ | R_3_ | |  | | | |  |  |
| **[4]** | H | | H | H | | 3-O-β-D-Glc | | | | **[130]** | Krokhmalyuk and Kintya 1976b; Maisashvili et al. 2008; Gugunishvili et al. 2006 |
| 3-O-α-L-Rha-(1→2)-O-β-D-Glc | | | | **[131]** | Chen and Snyder 1989; Timité et al. 2013 |
| 3-O-α-L-Rha-(1→2)-O-α-L-Ara | | | | **[132]** | Kravets et al. 1990 |
| 3-O-β-D-Gal-(1→4)-O-α-L-Rha-(1→2)-O-α-L-Ara | | | | **[133]** | Kravets et al. 1990 |
| 3-O-α-L-Rha-(1→2)-[β-D-Glc-(1→4)]-O-β-D-Glc | | | | **[134]** | Chen and Snyder 1989; Mimaki et al. 1996; Timité et al. 2013 |
| 3-O-α-L-Rha-(1→2)-[α-L-Rha-(1→4)]-O-β-D-Glc | | | | **[135]** | Jung et al. 1993; Sata et al. 1998 |
| 3-O-β-D-Glc-(1→4)-O-α-L-Rha-(1→4)-O-β-D-Glc | | | | **[136]** | Chen and Snyder 1989 |
| 3-O-α-L-Rha-(1→4)-[β-D-Glc-(1→2)]-O-β-D-Glc | | | | **[137]** | Rezugi et al. 2014 |
| 3-O-α-L-Rha-(1→2)-[β-D-Glc-(1→4)]-O-β-D-Gal | | | | **[138]** | Akhov et al. 1999 |
| 3-O-α-L-Rha-(1→2)-[β-D-Xyl-(1→4)]-O-β-D-Glc | | | | **[139]** | Mimaki et al. 1996 |
| 3-O-α-L-Rha-(1→2)-[β-D-Glc-(1→3)]-O-β-D-Glc | | | | **[140]** | Inoue et al. 1995 |
| 3-O-α-L-Rha-(1→4)-O-α-L-Rha-(1→4)-[α-L-Rha-(1→2)]-O-β-D-Glc | | | | **[141]** | Inoue et al. 1995; Mimaki et al. 1996; Jung et al. 1993; Sobolewska et al. 2006 |
| 3-O-β-D-Glc-(1→4)-O-α-L-Rha-(1→4)-[α-L-Rha-(1→2)]-O-β-D-Glc | | | | **[142]** | Chen and Snyder 1989 |
| 3-O-β-D-Glc-(1→2)-[β-D-Glc-(1→3)]-O-β-D-Gal-(1→4)-O-α-L-Rha-(1→2)-O-α-L-Ara | | | | **[143]** | Kintya and Degtyareva 1989 |
| 3-O-β-D-Glc-(1→3)-[β-D-Glc-(1→6)]-O-β-D-Glc-(1→4)-[α-L-Rha-(1→2)]-O-β-D-Glc | | | | **[144]** | Chen and Snyder 1989 |
| 3-O-β-D-Glc-(1→6)-O-β-D-Glc-(1→4)-O-α-L-Rha-(1→4)-[α-L-Rha-(1→2)]-O-β-D-Glc | | | | **[145]** | Chen and Snyder 1989 |
| 3-O-β-D-Glc-(1→4)-[β-D-Glc-(1→6)]-O-β-D-Glc-(1→4)-O-α-L-Rha-(1→4)-[α-L-Rha-(1→2)]-O-β-D-Glc | | | | **[146]** | Chen and Snyder 1989 |
| **[17]** | β-OH | | H | H | | 1-O-β-D-Gal | | | | **[147]** | Akhov et al. 1999 |
| **[19]** | H | | OH | H | | 3-O-α-L-Rha-(1→2)-O-β-D-Gal | | | | **[148]** | Do et al. 1992 |
| 3-O-α-L-Rha-(1→2)-[β-D-Glc-(1→3)]-O-β-D-Gal | | | | **[149]** | Do et al. 1992 |
| 3-O-β-D-Glc-(1→3)-[β-D-Glc-(1→4)]-O-β-D-Gal | | | | **[150]** | Do et al. 1992 |
| 3-O-β-D-Glc-(1→2)-[β-D-Xyl-(1→3)]-O-β-D-Glc-(1→4)-O-β-D-Gal | | | | **[151]** | Vollerner et al. 1978; Kravets 1994; Mimaki et al. 1999c; Uchida et al. 2009 |
| 3-O-β-D-Glc-(1→2)-[4-O-β-hydroxy-β-methylglutaryl-β-D-Xyl-(1→3)]-O-β-D-Glc-(1→4)-O-β-D-Gal | | | | **[152]** | Vollerner et al. 1983a; Kravets 1994 |
| 3-O-β-D-Xyl-(1→3)-[β-D-Gal-(1→2)]-O*-*β-D-Gal-(1→4)-O-β-D-Gal | | | | **[153]** | Rezugi et al. 2014 |
| 3-O-β-D-Xyl-(1→3)-[β-D-Glc-(1→2)]-O*-*β-D-Gal-(1→4)-O-β-D-Gal | | | | **[154]** | Rezugi et al. 2014 |
| **[28]** | H | | H | OH | | 3-O-α-L-Rha-(1→2)-O-β-D-Glc | | | | **[155]** | Chen and Snyder 1989 |
|  | **[5]** | 3-O-α-L-Rha-(1→4)-O-α-L-Rha-(1→4)-[α-L-Rha-(1→2)]-O-β-D-Glc | | | | | | | | | **[156]** | Sobolewska et al. 2006 |
|  | **[6]** | 3-O-α-L-Ara-(1→6)-O-β-D-Glc | | | | | | | | | **[157]** | Kuroda et al. 1995; Peng et al. 1996b; Baba et al. 2000 |
| 3-O-α-L-Rha-(1→2)-O-β-D-Glc | | | | | | | | | **[158]** | Timité et al. 2013 |
| 3-O-(2-O-acetyl-α-L-Ara)-(1→6)-O-β-D-Glc | | | | | | | | | **[159]** | Kuroda et al. 1995 |
| 3-O-α-L-Rha-(1→2)-[β-D-Glc-(1→4)]-O-β-D-Glc | | | | | | | | | **[160]** | Timité et al. 2013 |
| 3-O-β-D-Xyl-(1→4)-[α-L-Ara-(1→6)]-O-β-D-Glc | | | | | | | | | **[161]** | Peng et al. 1995; Peng et al. 1996b; Baba et al. 2000 |
|  |  | R_1_ | |  | | | | | | |  |  |
| **[7]** | H | | -------------------------------------- | | | | | | |  | Kravets et al. 1990;  Maisashvili et al. 2007 |
| **[29]** | OH | | 3-O-β-D-Glc-(1→2)-[β-D-Xyl-(1→3)]-O-β-D-Glc-(1→4)-O-β-D-Gal | | | | | | | **[162]** | Fattorusso et al. 2000 |
|  |  | R_1_ | | R_2_ | R_3_ | | R_4_ |  | | |  |  |
| **[8]** | OH | | H | OH | | H | 3-O-α-L-Rha-(1→4)-O-β-D-Glc | | | **[163]** | Sang et al. 2002 |
| **[11]** | H | | OH | OH | | H | 3-O-β-D-Glc-(1→2)-[α-L-Rha-(1→4)]-O-β-D-Glc | | | **[164]** | Sang et al. 2003 |
| **[13]** | H | | H | H | | OH | 3-O-α-L-Rha-(1→4)-O-β-D-Glc | | | **[165]** | Ikeda et al. 2000 |
| **[32]** | H | | OH | OH | | H | 3-O-β-D-Glc | | | **[166]** | Sang et al. 2003 |
| 3-O-α-L-Rha-(1→4)-O-β-D-Glc | | | **[167]** | Sang et al. 2003 |
| **[43]** | H | | H | OH | | OH | 3-O-α-L-Rha-(1→4)-O-β-D-Glc | | | **[168]** | Ikeda et al. 2000 |
|  |  | R_1_ | | R_2_ |  | | | | | |  |  |
| **[18]** | OH | | H | 1-O-α-L-Rha-(1→2)-O-α-L-Ara | | | | | | **[169]** | Kravets et al. 1986a; Teshima et al. 2013 |
| 1-O-α-L-Rha-(1→2)-O-α-D-Gal | | | | | | **[170]** | Kravets et al. 1986b; Teshima et al. 2013 |
| **[20]** | H | | OH | 3-O-α-L-Rha-(1→4)-[α-L-Rha-(1→2)]-O-β-D-Glc | | | | | | **[171]** | Ikeda et al. 2000 |
|  |  | R_1_ | | R_2_ |  | | | | | |  |  |
| **[21]** | OH | | H | 3-O-β-D-Glc-(1→2)-O-β-D-Gal | | | | | | **[172]** | He et al. 2002; Cheng et al. 2013 |
| **[22]** | H | | OH | 3-O-β-D-Glc-(1→2)-O-β-D-Gal | | | | | | **[173]** | Cheng et al. 2013 |
|  | **[23]** | 3-O-β-D-Glc-(1→3)-O-β-D-Glc-(1→2)-[β-D-Xyl-(1→3)]-O-β-D-Glc-(1→4)-O-β-D-Gal | | | | | | | | | **[174]** | Sata et al. 1998 |
| 3-O-β-D-Glc-(1→3)-O-β-D-Glc-(1→2)-[β-D-Glc-(1→3)]-O-β-D-Glc-(1→4)-O-β-D-Gal | | | | | | | | | **[175]** | Adão et al. 2012 |
|  | **[24]** | ------------------------------------------------ | | | | | | | | |  | Carotenuto et al. 1997b |
|  | **[25]** | ------------------------------------------------ | | | | | | | | |  | Pirtskhalava et al. 1977a |
|  | **[26]** | ------------------------------------------------- | | | | | | | | |  | Vollerner et al. 1988a; Vollerner et al. 1988b |
|  | **[27]** | 3-O-α-L-Rha-(1→2)-O-β-D-Glc | | | | | | | | | **[176]** | Chen and Snyder 1989 |
|  | **[30]** | 3-O-β-D-Glc-(1→2)-[β-D-Xyl-(1→3)]-O-β-D-Glc-(1→4)-O-β-D-Gal | | | | | | | | | **[177]** | Fattorusso et al. 2000 |
|  | **[44]** | 1-O-α-L-Rha-(1→2)-O-α-L-Ara | | | | | | | | | **[178]** | Kravets et al. 1987 |
| 1-O-α-L-Rha-(1→2)-O-α-D-Gal | | | | | | | | | **[179]** | Kravets et al. 1987 |
|  | **[45]** | 3-O-β-D-Xyl-(1→3)-[β-D-Glc-(1→2)]-O-β-D-Glc-(1→4)-O-β-D-Gal | | | | | | | | | **[180]** | Vollerner et al. 1984 |
| 3-O-β-D-Xyl-(1→3)-[β-D-Glc-(1→2)]-O-β-D-Glc-(1→4)-O-β-D-Gal 24-O-β-D-Glc | | | | | | | | | **[181]** | Vollerner et al. 1984 |
|  |  | R_1_ | R_2_ | | |  | | | | |  |  |
| **[46]** | OH | H | | | 3-O-α-L-Rha-(1→2)-O-β-D-Glc | | | | | **[182]** | Timité et al. 2013 |
| **[47]** | H | OH | | | 3-O-α-L-Rha-(1→ 2)-O-β-D-Glc | | | | | **[183]** | Timité et al. 2013 |
|  | **[48]** | ------------------------------------------------- | | | | | | | | |  | Vollerner et al. 1988a |
|  |  | R_1_ | |  | | | | | | |  |  |
| **[51]** | acetyl | | 2-O-β-D-Glc | | | | | | | **[184]** | Sashida et al. 1991; Mimaki et al. 1993; Mimaki et al. 1999c |
| **[54]** | 2-hydroxybutyryl | | 2-O-β-D-Glc | | | | | | | **[185]** | Mimaki et al. 1999c |
|  | **[52]** | 2-O-β-D-Glc | | | | | | | | | **[186]** | Inoue et al. 1995; Mimaki et al. 1999c; Sashida et al. 1991 |
|  | **[53]** | 3-O-β-D-Glc | | | | | | | | | **[187]** | Khristulas et al. 1974 |
|  |  | R_1_ |  | | | | | | | |  |  |
| **[55]** | α-OH | 3-O-α-L-Rha-(1→2)-[α-L-Rha-(1→4)]-O-β-D-Glc | | | | | | | | **[188]** | Hu et al. 2014 |
| **[56]** | β-OH | 3-O-α-L-Rha-(1→4)-O-β-D-Glc | | | | | | | | **[189]** | Sang et al. 2003 |
|  | **[58]** | 24-O-β-D-Glc | | | | | | | | | **[190]** | Vollerner et al. 1989 |
|  | **[59]** | ------------------------------------------------- | | | | | | | | |  | Kravets et al. 1990 |
|  | **[60]** | 24-O-β-D-Glc | | | | | | | | | **[191]** | Kawashima et al. 1991a |
|  | **[61]** | 2-O-β-D-Glc 24-O-β-D-Glc-(1→2)-O-β-D-Glc | | | | | | | | | **[192]** | Mimaki et al. 1999c |
|  |  | R_1_ |  | | | | | | | |  |  |
| **[62]** | acetyl | 2-O-β-D-Glc | | | | | | | | **[193]** | Mimaki et al. 1994 |
| **[63]** | benzoyl | 2-O-β-D-Glc | | | | | | | | **[194]** | Mimaki et al. 1999c |
| 2-O-β-D-Glc 24-O-β-D-Glc | | | | | | | | **[195]** | Mimaki et al. 1999c |
|  | **[64]** | ------------------------------------------------- | | | | | | | | |  | Carotenuto et al. 1997 |
