# Supplementary material for: Steroidal saponins from the genus Allium
Source: Phytochem Rev. 2014 Oct 8;15(1):1–35. doi: 10.1007/s11101-014-9381-1 (PMC4735241; doi:10.1007/s11101-014-9381-1)
Supplement: Supplementary file 2 — Supplementary material 2 (DOC 178 kb) [file 11101_2014_9381_MOESM2_ESM.doc]

Table 4. Cytotoxic activity of *Allium* saponins/sapogenins.

| **Species** | **Compound [No.]** | **Cell line** | **Concentration (IC_50_)** | **References** |
| --- | --- | --- | --- | --- |
| ***A. ampeloprasum*** L. | aginoside **[93]** | P388 | 2.1 μg/mL | Sata et al. 1998 |
|  | yayoisaponins A-C **[96,174,95]** | P388 | 2.1 μg/mL | Sata et al. 1998 |
|  | dioscin **[135]** | P388 | 0.092 μg/mL | Sata et al. 1998 |
| ***A. flavum*** L. | yuccagenin 3-O-β-D-Xyl-(1→3)-[β-D-Gal-(1→2)]-O*-*β-D-Gal-(1→4)-O-β-D-Gal **[153]** | SW480 | 14.3 μM | Rezgui et al. 2014 |
|  | yuccagenin 3-O-β-D-Xyl-(1→3)-[β-D-Glc-(1→2)]-O*-*β-D-Gal-(1→4)-O-β-D-Gal **[154]** | SW480 | 14.0 μM | Rezgui et al. 2014 |
|  | diosgenin 3-O-α-L-Rha-(1→4)-[β-D-Glc-(1→2)]-O-β-D-Glc **[137]** | SW480 | 18.1 μM | Rezgui et al. 2014 |
|  | Doxorubicin - control | SW480 | 1.47 μM | Rezgui et al. 2014 |
| ***A. jesdianum*** Boiss. | F-gitonin **[72]** | HL-60 | 1.5 μg/mL | Mimaki et al. 1999a |
|  | gantogenin 3-O-β-D-Glc-(1→2)-[β-D-Xyl-(1→3)]-O-β-D-Glc-(1→4)-O-β-D-Gal **[86]** | HL-60 | > 10 μg/mL | Mimaki et al. 1999a |
|  | Etoposide -control | HL-60 | 0.3 μg/mL | Mimaki et al. 1999a |
| ***A. karataviense*** Rgl. | karatavioside A **[151]** | HL-60 | 2.4 μg/mL | Mimaki et al. 1999c |
|  | Etoposide - control |  | 0.3 μg/mL | Mimaki et al. 1999c |
| ***A. leucanthum*** C. Koch | yayoisaponin C **[95]** | A549  DLD-1  WS1 | 3.7 ± 0.7 μM  5.6 ± 0.2 μM  3.0 ± 0.2 μM | Mskhiladze et al. 2008b |
|  | eruboside B **[79]** | A549  DLD-1  WS1 | 5.3 ± 0.5 μM  8.2 ± 0.4 μM  3.6 ± 0.2 μM | Mskhiladze et al. 2008b |
|  | aginoside **[93]** | A549  DLD-1  WS1 | 5.8 ± 0.9 μM  7.9 ± 0.5 μM  3.6 ± 0.2 μM | Mskhiladze et al. 2008b |
|  | β-chlorogenin 3-O-β-D-Glc-(1→2)-[β-D-Xyl-(1→3)]-O-β-D-Glc-(1→4)-O-β-D-Gal **[80]** | A549  DLD-1  WS1 | 9 ± 1 μM  13 ± 1 μM  3.1 ± 0.1 μM | Mskhiladze et al. 2008b |
|  | leucospiroside A **[97]** | A549  DLD-1  WS1 | 5.0 ± 0.1 μM  7.2 ± 0.1 μM  4.55 ± 0.07 μM | Mskhiladze et al. 2008b |
|  | agigenin 3-O-β-D-Glc-(1→2)-O-β-D-Glc-(1→4)-O-β-D-Gal **[91]** | A549  DLD-1  WS1 | 22 ± 2 μM  22 ± 2 μM  14.5 ± 0.5 μM | Mskhiladze et al. 2008b |
|  | β-chlorogenin 3-O-β-D-Glc-(1→3)-O-β-D-Glc-(1→2)-[β-D-Glc-(1→3)]-O-β-D-Glc-(1→4)-O-β-D-Gal **[81]** | A549  DLD-1  WS1 | 7.8 ± 0.4 μM  8.9 ± 0.2 μM  7.7 ± 0.2 μM | Mskhiladze et al. 2008b |
|  | Etoposide -control | A549  DLD-1  WS1 | 1.1 ± 0.1 μM  4.8 ± 0.8 μM  n.d. | Mskhiladze et al. 2008b |
|  | 5-fluorouracil - control | A549  DLD-1  WS1 | 48 ± 18 μM  11 ± 2 μM  20 ± 2 μM | Mskhiladze et al. 2008b |
| ***A. macleanii***  Baker | tigogenin 3-O-α-L-Rha-(1→2)-O-β-D-Xyl-(1→2)-[β-D-Xyl-(1→3)]-O-β-D-Glc-(1→4)-O-β-D-Gal **[67]** | HeLa | Cytotoxic at 50 μg/mL | Inoue et al. 1995 |
| ***A. macrostemon*** Bunge | 26-O-β-D-Glc 5α-furost-25(27)-ene-3β,12β,22,26-tetrol 3-O-β-D-Glc-(1→2)-[β-D-Glc-(1→3)]-O-β-D-Glc-(1→4)-O-β-D-Gal **[292]** | NCI-H460  SF-268  MCF-7  HepG2 | >100 μM  35.2 ± 1.02 μM  >100 μM  >100 μM | Chen et al. 2009 |
|  | 26-O-β-D-Glc 5β-furost-20(22),25(27) -diene-3β,12β,26-triol 3-O-β-D-Glc-(1→2)-O-β-D-Gal **[293]** | NCI-H460  SF-268  MCF-7  Hep2 | 25.7 ± 0.62 μM  35.4 ± 0.71 μM  >100 μM  >100 μM | Chen et al. 2009 |
| ***A. nigrum*** L. | nigrosides A1/A2 **[89,117]** | HCT-116  HT-29 | 47.8 μM  70.8 μM | Jabrane et al. 2011 |
|  | nigrosides B1/B2 **[88,118]** | HCT-116  HT-29 | >100 μM  >100 μM | Jabrane et al. 2011 |
|  | nigroside C **[303]** | HCT-116  HT-29 | >100 μM  >100 μM | Jabrane et al. 2011 |
|  | nigroside D **[304]** | HCT-116  HT-29 | >100 μM  >100 μM | Jabrane et al. 2011 |
|  | aginoside/turoside A **[93,122]** | HCT-116  HT-29 | 1.59 μM  1.09 μM | Jabrane et al. 2011 |
|  | **(25*R*,*S*)-5α-spirostane-2α,3β,6β-triol 3-O-β-D-Glc-(1→2)-[4-O-(*S*)-3-hydroxy-3-methylglutaryl-β-D-Xyl-(1→3)]-O-β-D-Glc-(1→4)-O-β-D-Gal [98,124]** | HCT-116  HT-29 | 3.45 μM  2.82 μM | Jabrane et al. 2011 |
|  | Paclitaxel | HCT-116  HT-29 | 0.00321 μM  0.00140 μM | Jabrane et al. 2011 |
| ***A. porrum*** L. | porrigenin B **[23]** | IGR-1  J-774  WHEI 164  P-388 | (72 h) 45.0 ± 13.0 μg/mL  (72 h) 51.0 ± 21.0 μg/mL  (72 h) 92.0 ± 19.0 μg/mL  (72 h) 74.0 ± 22.0 μg/mL | Carotenuto et al. 1997a |
|  | F-gitonin **[72]** | J-774  WHEI 164 | 3.7 μg/mL  4.8 μg/mL | Fattorusso et al. 2000 |
|  | gitogenin 3-O-β-D-Glc-(1→3)-O-β-D-Glc-(1→2)-[β-D-Xyl-(1→3)]-O-β-D-Glc-(1→4)-O-β-D-Gal **[74]** | J-774  WHEI 164 | 2.1 μg/mL  1.9 μg/mL | Fattorusso et al. 2000 |
|  | β-chlorogenin 3-O-β-D-Glc-(1→2)-[β-D-Xyl-(1→3)]-O-β-D-Glc-(1→4)-O-β-D-Gal **[80]** | J-774  WHEI 164 | 5.7 μg/mL  6.5 μg/mL | Fattorusso et al. 2000 |
|  | β-chlorogenin 3-O-β-D-Glc-(1→3)-O-β-D-Glc-(1→2)-[β-D-Xyl-(1→3)]-O-β-D-Glc-(1→4)-O-β-D-Gal **[82]** | J-774  WHEI 164 | 7.6 μg/mL  10.0 μg/mL | Fattorusso et al. 2000 |
|  | porrigenin C 3-O-β-D-Glc-(1→2)-[β-D-Xyl-(1→3)]-O-β-D-Glc-(1→4)-O-β-D-Gal **[177]** | J-774  WHEI 164 | 27.9 μg/mL  21.1 μg/mL | Fattorusso et al. 2000 |
|  | 12-ketoporrigenin 3-O-β-D-Glc-(1→2)-[β-D-Xyl-(1→3)]-O-β-D-Glc-(1→4)-O-β-D-Gal **[162]** | J-774  WHEI 164 | 5.8 μg/mL  4.3 μg/mL | Fattorusso et al. 2000 |
|  | alliosterol 1-O-α-L- Rha 16-O-β-D-Glc **[267]** | J-774  WHEI 164 | 4.6 μg/mL  5.8 μg/mL | Fattorusso et al. 2000 |
|  | alliosterol 1-O-β-D-Glc-(1→4)-O-α-L- Rha 16-O-β-D-Gal **[308]** | J-774  WHEI 164 | 4.0 μg/mL  5.4 μg/mL | Fattorusso et al. 2000 |
|  | 6-MP - control | IGR-1  J-774  WHEI 164  P-388 | (24 h) 45.0 ± 13.0 μg/mL  (48 h) 51.0 ± 21.0 μg/mL  (72 h) 92.0 ± 19.0 μg/mL  (24 h) 45.0 ± 13.0 μg/mL  (48 h) 51.0 ± 21.0 μg/mL  (72 h) 92.0 ± 19.0 μg/mL  (24 h) 74.0 ± 22.0 μg/mL  (48 h) 45.0 ± 13.0 μg/mL  (72 h) 51.0 ± 21.0 μg/mL  (24 h) 92.0 ± 19.0 μg/mL  (48 h) 74.0 ± 22.0 μg/mL  (72 h) 45.0 ± 13.0 μg/mL | Fattorusso et al. 2000 |
| ***A. schoenoprasum*** L. | (25*R*)-5α-spirostane-3β,11α-diol 3-O-β-D-Glc-(1→3)-[β-D-Glc-(1→4)]-O-β-D-Gal **[83]** | HCT 116  HT-29 | 8.45 μM  8.64 μM | Timité et al. 2013 |
|  | laxogenin 3-O-α-L-Rha-(1→2)-O-β-D-Glc **[158]** | HCT 116  HT-29 | >100 μM  >100 μM | Timité et al. 2013 |
|  | deltonin **[134]** | HCT 116  HT-29 | 0.40 μM  0.75 μM | Timité et al. 2013 |
|  | deltoside **[306]** | HCT 116  HT-29 | 1.58 μM  1.56 μM | Timité et al. 2013 |
|  | Paclitaxel - control | HCT 116  HT-29 | 0.00275 μM  0.00206 μM | Timité et al. 2013 |
| ***A. senescens*** L. | diosgenin 3-O-α-L-Rha-(1→2)-[β-D-Glc-(1→3)]-O-β-D-Glc **[140]** | HeLa | Cytotoxic at 50 μg/mL | Inoue et al. 1995 |
| ***A. tuberosum*** Rottl. | 26-O-β-D-Glc (25*R*)-5α-furostane-3β,22,26-triol 3-O-α-L-Rha-(1→4)-[α-L-Rha-(1→2)]-O-β-D-Glc **[351]** | PC-12  HCT-116 | No activity at less than 5 μM | Ikeda et al. 2004 |
|  | 26-O-β-D-Glc (25*S*)-5β-furostane-3β,5β,6α,22,26-pentaol 3-O-α-L-Rha-(1→4)-O-β-D-Glc **[352]** | PC-12  HCT-116 | No activity at less than 5 μM | Ikeda et al. 2004 |
|  | tuberoside M **[163]** | HL-60 | 6.8 μg/mL | Sang et al. 2002 |
| ***A. ursinum*** L. | a mixture of diosgenin 3-O-α-L-Rha-(1→4)-O-α-L-Rha-(1→4)-[α-L-Rha-(1→2)]-O-β-D-Glc and (25*R*)-spirost-5(6),25(27)-diene-3β-ol 3-O-α-L-Rha-(1→4)-O-α-L-Rha-(1→4)-[α-L-Rha-(1→2)]-O-β-D-Glc **[141,156]** | melanoma B16  sarcoma XC | 100 % effect at 2 µg/mL  100 % effect at 2 µg/mL | Sobolewska et al. 2006 |
| ***A. vavilovii*** M. Pop. & Vved. | vavilosides B1/B2 **[357,358]** | J-774  WEHI-164 | 3.5 μg/mL  3.1 μg/mL | Zolfaghari et al. 2013 |
|  | ascalonicosides A1/A2 **[217,218]** | J-774  WEHI-164 | 4.0 μg/mL  3.7 μg/mL | Zolfaghari et al. 2013 |
|  | vavilosides A1/A2 **[355,356]** | J-774  WEHI-164 | 5.1 μg/mL  4.7 μg/mL | Zolfaghari et al. 2013 |
| ***A. victorialis*** var. ***platyphyllum*** L. | F-gitonin **[72]** | HepC-2  Vero-P128  P-388  L-1210 | 17.9 μg/mL  14.6 μg/mL  36.5 μg/mL  6.5 μg/mL | Lee et al. 2001  Lee et al. 2001  Lee et al. 2001  Lee et al. 2001 |

Table 5. *In vitro* antifungal properties of active saponins from different *Allium* species.

| **Species** | **Compound** | **Fungal strain** | **Activity** | **References** |
| --- | --- | --- | --- | --- |
| ***A. ampeloprasum*** L. | aginoside **[93]** | *Mortierella ramanniana* | Inhibition zone  27 mm (100 μg/disc)  17 mm (10 μg/disc) | Sata et al. 1998 |
|  | yayoisaponin A **[96]** | *Mortierella ramanniana* | 23 mm (100 μg/disc)  12 mm (10 μg/disc) | Sata et al. 1998 |
|  | yayoisaponin B **[174]** | *Mortierella ramanniana* | 20 mm (100 μg/disc)  0 mm (10 μg/disc) | Sata et al. 1998 |
|  | yayoisaponin C **[95]** | *Mortierella ramanniana* | 26 mm (100 μg/disc)  13 mm (10 μg/disc) | Sata et al. 1998 |
| ***A. ampeloprasum*** L. *ssp.* ***persicum*** | persicoside A **[120]** | *Penicilium italicum*  *Aspergillus niger*  *Botrytis cinerea*  *Trichoderma harzianum* | Fungal growth compared to control (= 100 %)  ~40 % (at 100 and 1000 ppm)  ~20 % (at 100 and 1000 ppm)  Not active  ~40 % (at 100 and 1000 ppm) | Sadeghi et al. 2013 |
|  | persicoside B **[121]** | *Penicilium italicum*  *Aspergillus niger*  *Botrytis cinerea*  *Trichoderma harzianum* | ~40 % (at 100 and 1000 ppm)  ~40 % (at 100 and 1000 ppm)  Not active  ~40 % (at 100 and 1000 ppm) | Sadeghi et al. 2013 |
|  | persicosides C1/C2 **[205,206]** | *Penicilium italicum*  *Aspergillus niger*  *Botrytis cinerea*  *Trichoderma harzianum* | Not acive  Not active  Not active  >80 % (at 1000 ppm) | Sadeghi et al. 2013 |
|  | persicoside E **[219]** | *Penicilium italicum*  *Aspergillus niger*  *Botrytis cinerea*  *Trichoderma harzianum* | ~50 % (at 100 ppm)  Not active  Not active  ~70 % (at 1000 ppm) | Sadeghi et al. 2013 |
|  | ceposides A1/A2 **[209,210]** | *Penicilium italicum*  *Aspergillus niger*  *Botrytis cinerea*  *Trichoderma harzianum* | ~40 % (at 1000 ppm)  ~30 % (at 1000 ppm)  ~60 % (at 1000 ppm)  ~40 % (at 1000 ppm) | Sadeghi et al. 2013 |
|  | ceposides C1/C2 **[211,212]** | *Penicilium italicum*  *Aspergillus niger*  *Botrytis cinerea*  *Trichoderma harzianum* | ~50 % (at 10 ppm)  >80 % (at 10, 100, 1000 ppm)  Not active  ~80 % (at 10, 100, 1000 ppm) | Sadeghi et al. 2013 |
|  | tropeosides A1/A2 **[213,214]** | *Penicilium italicum*  *Aspergillus niger*  *Botrytis cinerea*  *Trichoderma harzianum* | ~50 % (at 1000 ppm)  ~70 % (at 1000 ppm)  Not active  ~40 % (at 1000 ppm) | Sadeghi et al. 2013 |
|  | tropeosides B1/B2 **[215,216]** | *Penicilium italicum*  *Aspergillus niger*  *Botrytis cinerea*  *Trichoderma harzianum* | ~40 % (at 1000 ppm)  ~50 % (at 1000 ppm)  Not active  ~50 % (at 1000 ppm) | Sadeghi et al. 2013 |
| ***A. cepa*** L. *var.* ***aggregatum*** | alliospiroside A **[169]** | *Alternaria tenuissima*  *Botrytis cinerea*  *B. squamosa*  *Colletotrichum acutatum*  *C. destructivum*  *C. gloeosporioides*  *Curvularia lunata*  *Epicoccum nigrum*  *Fusarium oxysporum* f.sp*. melonis*  *F. solani*  *F. proliferatum*  *Magnaporthe oryzae*  *Sclerotium cepivorum* | % growth inhibition (at 100 ppm)  ~84 %  ~55 %  ~35 %  ~96 %  ~84 %  ~100 %  ~73 %  ~96 %  ~37 %  ~38 %  ~77 %  ~96 %  ~93 % | Teshima et al. 2013 |
|  | alliospiroside B **[170]** | *Alternaria tenuissima*  *Botrytis cinerea*  *B. squamosa*  *Colletotrichum acutatum*  *C. destructivum*  *C. gloeosporioides*  *Curvularia lunata*  *Epicoccum nigrum*  *Fusarium oxysporum* f.sp*. melonis*  *F. solani*  *F. proliferatum*  *Magnaporthe oryzae*  *Sclerotium cepivorum* | ~38 %  ~20 %  ~18 %  ~57 %  ~63 %  ~71 %  ~63 %  ~70 %  ~15 %  ~20 %  ~53 %  ~58 %  ~56 % | Teshima et al. 2013 |
| ***A. fistulosum*** L. | fistuloside A **[148]** | *Candida albicans* ATCC 10231  *Saccharomyces cerevisiae* IFO 0233 | MIC (MFC)  25 μg/mL (25 μg/mL)  50 μg/mL (100 μg/mL) | Sohn et al. 2006 |
|  | fistuloside B **[149]** | *Candida albicans* ATCC 10231  *Saccharomyces cerevisiae* IFO 0233 | 50 μg/mL (50 μg/mL)  50 μg/mL (50 μg/mL) | Sohn et al. 2006 |
|  | fistuloside C **[150]** | *Candida albicans* ATCC 10231  *Saccharomyces cerevisiae* IFO 0233 | 6.2 μg/mL (6.2 μg/mL)  3.1 μg/mL (3.1 μg/mL) | Sohn et al. 2006 |
|  | Miconazole - control | *Candida albicans* ATCC 10231  *Saccharomyces cerevisiae* IFO 0233 | 1.5 μg/mL (1.5 μg/mL)  1.5 μg/mL (1.5 μg/mL) | Sohn et al. 2006 |
| ***A. leucanthum*** C. Koch | yayoisaponin C **[95]** | *Candida albicans* ATCC 90029  *C. albicans* ATCC 38248  *C. albicans* Y0109  *C. tropicalis I*P 1275-8  *C. parapsilosis* ATCC 22019  *C. glabrata* ATCC 90030  *C. kefyr* Y0601  *C. krusei* ATCC 6258  *C. lusitaniae* CBS 6936  *Cryptococcus neoformans* | MFC  50 μg/mL  50 μg/mL  50 μg/mL  >100 μg/mL  50 μg/mL  25 μg/mL  100 μg/mL  50 μg/mL  >100 μg/mL  12.5 μg/mL | Mskhiladze et al. 2008a |
|  | eruboside B **[79]** | *Candida albicans* ATCC 90029  *C. albicans* ATCC 38248  *C. albicans* Y0109  *C. tropicalis* IP 1275-8  *C. parapsilosis* ATCC 22019  *C. glabrata* ATCC 90030  *C. kefyr* Y0601  *C. krusei* ATCC 6258  *C. lusitaniae* CBS 6936  *Cryptococcus neoformans* | 25 μg/mL  12.5 μg/mL  25 μg/mL  50 μg/mL  12.5 μg/mL  12.5 μg/mL  25 μg/mL  25 μg/mL  50 μg/mL  6.25 | Mskhiladze et al. 2008a |
|  | aginoside **[93]** | *Candida albicans* ATCC 90029  *C. albicans* ATCC 38248  *C. albicans* Y0109  *C. tropicalis* IP 1275-8  *C. parapsilosis* ATCC 22019  *C. glabrata* ATCC 90030  *C. kefyr* Y0601  *C. krusei* ATCC 6258  *C. lusitaniae* CBS 6936  *Cryptococcus neoformans* | 25 μg/mL  12.5 μg/mL  12.5 μg/mL  50 μg/mL  6.25 μg/mL  6.25 μg/mL  12.5 μg/mL  12.5 μg/mL  50 μg/mL  6.25 μg/mL | Mskhiladze et al. 2008a |
|  | β-chlorogenin 3-O-β-D-Glc-(1→2)-[β-D-Xyl-(1→3)]-O-β-D-Glc-(1→4)-O-β-D-Gal **[80]** | *Candida albicans* ATCC 90029  *C. albicans* ATCC 38248  *C. albicans* Y0109  *C. tropicalis* IP 1275-8  *C. parapsilosis* ATCC 22019  *C. glabrata* ATCC 90030  *C. kefyr* Y0601  *C. krusei* ATCC 6258  *C. lusitaniae* CBS 6936  *Cryptococcus neoformans* | 12.5 μg/mL  12.5 μg/mL  12.5 μg/mL  25 μg/mL    ≤ 6.25 μg/mL    ≤ 6.25 μg/mL    6.25 μg/mL    ≤ 6.25 μg/mL  25 μg/mL  ≤ 6.25 μg/mL | Mskhiladze et al. 2008a |
|  | agigenin 3-O-β-D-Glc-(1→2)-O-β-D-Glc-(1→4)-O-β-D-Gal **[91]** | *Candida albicans* ATCC 90029  *C. albicans* ATCC 38248  *C. albicans* Y0109  *C. tropicalis* IP 1275-8  *C. parapsilosis* ATCC 22019  *C. glabrata* ATCC 90030  *C. kefyr* Y0601  *C. krusei* ATCC 6258  *C. lusitaniae* CBS 6936  *Cryptococcus neoformans* | 25 μg/mL  25 μg/mL  25 μg/mL  100 μg/mL  12.5 μg/mL  12.5 μg/mL  25 μg/mL  25 μg/mL  100 μg/mL  6.25 μg/mL | Mskhiladze et al. 2008a |
|  | Amphotericin -control | *Candida albicans* ATCC 90029  *C. albicans* ATCC 38248  *C. albicans* Y0109  *C. tropicalis* IP 1275-8  *C. parapsilosis* ATCC 22019  *C. glabrata* ATCC 90030  *C. kefyr* Y0601  *C. krusei* ATCC 6258  *C. lusitaniae* CBS 6936  *Cryptococcus neoformans* | 1.56 μg/mL  1.56 μg/mL  12.5 μg/mL  3.12 μg/mL  0.78 μg/mL  0.78 μg/mL  3.12 μg/mL  1.56 μg/mL  0.78 μg/mL | Mskhiladze et al. 2008a |
| ***A. minutiflorum*** Regel | minutoside A **[295]** | *Alternaria alternata*  *A. porri*  *Botrytis cinerea*  *Fusarium oxysporum*  *F. oxysporum ssp. lycopersici*  *F. solani*  *Pythium ultimum*  *Rhizoctonia solani*  *Trichoderma harzianum* P1  *T. harzianum* T39 | Fungal growth comp. to control (=100 %)  98 ± 7.8 % (at 100 ppm)  97 ± 8.2 % (at 10 ppm)  90 ± 9.8 % (at 100 ppm)  94 ± 6.3 % (at 10 ppm)  40 ± 6.8 % (at 100 ppm)  55 ± 9.3 % (at 10 ppm)  78 ± 10.2 % (at 100 ppm)  82 ± 6.9 % (at 10 ppm)  82 ± 10.9 % (at 100 ppm)  98 ± 22.3 % (at 10 ppm)  74 ± 9.7 % (at 100 ppm)  78 ± 6.8 % (at 10 ppm)  81 ± 0.2 % (at 100 ppm)  100 ± 0.0 % (at 10 ppm)  75 ± 0.0 % (at 100 ppm)  77 ± 0.0 % (at 10 ppm)  104 ± 5.9 % (at 100 ppm)  104 ± 7.3 % (at 10 ppm)  96 ± 1.8 % (at 100 ppm)  94 ± 1.1 % (at 10 ppm) | Barile et al. 2007 |
|  | minutoside B **[119]** | *Alternaria alternata*  *A. porri*  *Botrytis cinerea*  *Fusarium oxysporum*  *F. oxysporum ssp. lycopersici*  *F. solani*  *Pythium ultimum*  *Rhizoctonia solani*  *Trichoderma harzianum* P1  *T. harzianum* T39 | 62 ± 10.2 % (at 100 ppm)  89 ± 13.4 % (at 10 ppm)  77 ± 5.0 % (at 100 ppm)  85 ± 5.1 % (at 10 ppm)  21 ± 2.8 % (at 100 ppm)  42 ± 10.6 % (at 10 ppm)  71 ± 6.4 % (at 100 ppm)  68 ± 5.3 % (at 10 ppm)  73 ± 7.0 % (at 100 ppm)  76 ± 6.2 % (at 10 ppm)  12 ± 2.8 % (at 100 ppm)  51 ± 4.9 % (at 10 ppm)  89 ± 0.3 % (at 100 ppm)  98 ± 0.1% (at 10ppm)  49 ± 0.0% (at 100ppm)  70 ± 0.0% (at 10ppm)  0 % (at 100 ppm)  0 % (at 10 ppm)  0 % (at 100 ppm)  55 ± 0.0 % (at 10 ppm) | Barile et al. 2007 |
|  | minutoside C **[296]** | *Alternaria alternata*  *A. porri*  *Botrytis cinerea*  *Fusarium oxysporum*  *F. oxysporum ssp. lycopersici*  *F. solani*  *Pythium ultimum*  *Rhizoctonia solani*  *Trichoderma harzianum* P1  *T. harzianum* T39 | 74 ± 5.2 % (at 100 ppm)  115 ± 6.9 % (at 10 ppm)  63 ± 5.5 % (at 100 ppm)  98 ± 4.8 % (at 10 ppm)  98 ± 4.8 % (at 100 ppm)  73 ± 8.7 % (at 10 ppm)  79 ± 8.6 % (at 100 ppm)  87 ± 9.2 % (at 10 ppm)  62 ± 6.1 % (at 100 ppm)  84 ± 9.0 % (at 10 ppm)  44 ± 5.6 % (at 100 ppm)  48 ± 5.8 % (at 10 ppm)  100 ± 0.0 % (at 100 ppm)  100 ± 0.0 % (at 10 ppm)  54 ± 0.0 % (at 100 ppm)  106 ± 0.1 % (at 10 ppm)  0 % (at 100 ppm)  0 % (at 10 ppm)  77 ± 1.4 % (at 100 ppm)  100 ± 2.7 % (at 10 ppm) | Barile et al. 2007 |
|  | alliogenin **[49]** | *Alternaria alternata*  *A. porri*  *Botrytis cinerea*  *Fusarium oxysporum*  *F. oxysporum ssp. lycopersici*  *F. solani*  *Pythium ultimum*  *Rhizoctonia solani*  *Trichoderma harzianum* P1  *T. harzianum* T39 | 86 ± 7.7 % (at 100 ppm)  90 ± 7.7 % (at 10 ppm)  83 ± 6.5 % (at 100 ppm)  89 ± 6.4 % (at 10 ppm)  52 ± 7.0 % (at 100 ppm)  59 ± 15.8 % (at 10 ppm)  90 ± 7.3 % (at 100 ppm)  87 ± 8.6 % (at 10 ppm)  80 ± 10.0 % (at 100 ppm)  85 ± 11.3 % (at 10 ppm)  64 ± 9.9 % (at 100 ppm)  65 ± 6.3 % (at 10 ppm)  100 ± 0.0 % (at 100ppm)  100 ± 0.0 % (at 10 ppm)  60 ± 0.0 % (at 100 ppm)  70 ± 0.0 % (at 10 ppm)  58 ± 5.4 % (at 100 ppm)  103 ± 8.3 % (at 10 ppm)  94 ± 2.0 % (at 100 ppm)  96 ± 1.8 % (at 10 ppm) | Barile et al. 2007 |
|  | neoagigenin **[36]** | *Alternaria alternata*  *A. porri*  *Botrytis cinerea*  *Fusarium oxysporum*  *F. oxysporum ssp. lycopersici*  *F. solani*  *Pythium ultimum*  *Rhizoctonia solani*  *Trichoderma harzianum* P1  *T. harzianum* T39 | 100 ± 68 % (at 100 ppm)  95 ± 7.8 % (at 10 ppm)  93 ± 5.5 % (at 100 ppm)  93 ± 5.2 % (at 10 ppm)  31 ± 9.0 % (at 100 ppm)  66 ± 12.1 % (at 10 ppm)  56 ± 6.5 % (at 100 ppm)  73 ± 7.6 % (at 10 ppm)  81 ± 6.4 % (at 100 ppm)  97 ± 9.3 % (at 10 ppm)  28 ± 6.6 % (at 100 ppm)  26 ± 4.8 % (at 10 ppm)  100 ± 0.0 % (at 100 ppm)  100 ± 0.0 % (at 10 ppm)  43 ± 0.1 % (at 100 ppm)  54 ± 0.1 % (at 10 ppm)  0 % (at 100 ppm)  24 ± 1.2 % (at 10 ppm)  0 ± 0 % (at 100 ppm)  0 ± 0 % (at 10 ppm) | Barile et al. 2007 |
| ***A. nigrum*** L. | aginoside **[93]** | *Fusarium oxysporum f. sp. cepae*  *F. oxysporum f. sp. radicis lycopersici*  *F. veriticillioides*  *Botrytis squamosa*  *Colletotrichum gloeosporioides* | % growth inhibition compared to control (= 0)  ~35 % (at 100 ppm)  ~55 % (at 200 ppm)  ~68 % (at 400 ppm)  ~18 % (at 100 ppm)  ~30 % (at 200 ppm)  ~42 % (at 400 ppm)  ~35 % (at 100 ppm)  ~75 % (at 200 ppm)  ~100 % (at 400 ppm)  ~45 % (at 100 ppm)  ~81 % (at 200 ppm)  ~100 % (at 400 ppm)  ~73 % (at 100 ppm)  ~100 % (at 200 ppm)  ~98 % (at 400 ppm) | Mostafa et al. 2013 |
| ***A. porrum ***L. | β-chlorogenin 3-O-β-D-Glc-(1→2)-[β-D-Xyl-(1→3)]-O-β-D-Glc-(1→4)-O-β-D-Gal **[80]** | *Fusarium culmorum* | ED_50_s 30-35 μg/mL | Carotenuto et al. 1999 |
|  | β-chlorogenin 3-O-β-D-Glc-(1→3)-O-β-D-Glc-(1→2)-[β-D-Xyl-(1→3)]-O-β-D-Glc-(1→4)-O-β-D-Gal **[82]** |  |  |  |
|  | F-gitonin **[72]** |  |  |  |
|  | gitogenin 3-O-β-D-Glc-(1→3)-O-β-D-Glc-(1→2)-[β-D-Xyl-(1→3)]-O-β-D-Glc-(1→4)-O-β-D-Gal **[74]** |  |  |  |
| ***A. sativum*** L. | eruboside B **[79]** | *Candida albicans* | MIC  25 μg/mL | Matsuura et al. 1988 |
| ***A. sativum*** L. *var.* ***Voghiera*** | voghieroside A **[319,320]** | *Trichoderma harzianum*  *Botrytis cinerea* | Mycelium growth comp. to control (=100 %)  > 100 % (at 10 ppm)  > 100 % (at 100 ppm)  ~80 % (at 1000 ppm)  ~95 % (at 10 ppm)  ~98 % (at 100 ppm)  ~100 % (at 1000 ppm) | Lanzotti 2012a |
|  | voghieroside B **[321,322]** | *Trichoderma harzianum*  *Botrytis cinerea* | ~93 % (at 10 ppm)  > 100 % (at 100 ppm)  ~30 % (at 1000 ppm)  100 % (at 10 ppm)  > 100 % (at 100 ppm)  ~88 % (at 1000 ppm) | Lanzotti 2012a |
|  | voghieroside C **[323,324]** | *Trichoderma harzianum*  *Botrytis cinerea* | 100 % (at 10 ppm)  ~85 % (at 100 ppm)  ~15 % (at 1000 ppm)  ~87 % (at 10 ppm)  ~80 % (at 100 ppm)  ~44 % (at 1000 ppm) | Lanzotti 2012a |
|  | gitogenin 3-O-β-D-Glc-(1→2)-O-[β-D-Glc-(1→3)]-O-β-D-Glc-(1→4)-O-β-D-Gal **[73]** | *Trichoderma harzianum*  *Botrytis cinerea* | ~73 % (at 10 ppm)  ~57 % (at 100 ppm)  0 % (at 1000 ppm)  ~78 % (at 10 ppm)  ~68 % (at 100 ppm)  ~34 % (at 1000 ppm) | Lanzotti 2012a |
|  | ampeloside Bs_1_ **[90]** | *Trichoderma harzianum*  *Botrytis cinerea* | 0 % (at 10 ppm)  0 % (at 100 ppm)  0 % (at 1000 ppm)  100 % (at 10 ppm)  ~80 % (at 100 ppm)  ~34 % (at 1000 ppm) | Lanzotti 2012a |
| ***A. ursinum*** L. | a mixture of diosgenin 3-O-α-L-Rha-(1→4)-O-α-L-Rha-(1→4)-[α-L-Rha-(1→2)]-O-β-D-Glc and (25*R*)-spirost-5(6),25(27)-diene-3β-ol 3-O-α-L-Rha-(1→4)-O-α-L-Rha-(1→4)-[α-L-Rha-(1→2)] -O-β-D-Glc **[141,156]** | *Candida albicans*  *C. parapsilosis*  *Trichophyton mentagrophytes*  *Microsporum canis* | MIC  200 μg/mL  250 μg/mL  400 μg/mL  400 μg/mL | Sobolewska et al. 2003  Sobolewska et al. 2003  Sobolewska et al. 2006  Sobolewska et al. 2006 |
